# Targeted sequence capture of coxsackievirus A6 using nanopore sequencing directly from clinical specimens

**DOI:** 10.1128/spectrum.03246-25

**Published:** 2026-06-04

**Authors:** Ziqi Lin, Fenglan He, Han Mo, Lingfeng Mao, Xingyu Xu, Liu Yi, Ke Qian, Xiansheng Ni, Tielong Xu, Xianfeng Zhou, Hui Li

**Affiliations:** 1 Jiangxi Provincial Health Commission Key Laboratory of Pathogenic Diagnosis and Genomics of Emerging Infectious Diseases, Nanchang Center for Disease Control and Prevention 675466, Nanchang, China; 2 Evidence-based Medicine Research Center, Jiangxi University of Chinese Medicine 666011 , Nanchang, China; 3 Mass Spectrometry Diagnostics and Chronic Disease Rehabilitation Research Center, Jiangxi University of Chinese Medicine, Nanchang, China; 4 Hangzhou Baiyi Biotechnology Co., Ltd., Hangzhou, China; Arizona State University College of Health Solutions, Phoenix, Arizona, USA; Arizona State University Biodesign Institute, Tempe, Arizona, USA; Corewell Health, Royal Oak, Michigan, USA

**Keywords:** coxsackievirus A6, nanopore sequencing, clinical samples, genomic surveillance

## Abstract

Coxsackievirus A6 (CVA6) has emerged as a predominant causative agent of hand, foot, and mouth disease worldwide. Efficient genomic surveillance is crucial for understanding its evolution and spread. Nanopore sequencing presents a promising platform for rapid and cost-effective viral genome sequencing directly from clinical samples. We developed a tiling amplicon-based nanopore sequencing protocol for CVA6. Eight clinical CVA6-positive samples, spanning a wide range of Ct values (15.54–25.28), were each serially diluted to three concentrations (undiluted, 10⁻¹, and 10⁻²), generating a total of 24 libraries. These libraries were sequenced using a PromethION flow cell and a MinION flow cell on PromethION and GridION platforms, respectively. Sensitivity was assessed across the dilution series, and accuracy was benchmarked against Illumina sequencing. The protocol demonstrated high sensitivity and speed, achieving >10× average sequencing depth within 5 mins using a PromethION flow cell. High genome coverage (≥96.5%) was consistently achieved across all samples, including serial dilutions, with minimal genetic divergence: only one to five single-nucleotide variants were observed between different dilution levels of the same specimen. Complete assembly was obtained from all test samples in the 10^−2^ dilutions (Ct: 21.73–32.7). Comparative genomic analysis revealed 99.81%–99.98% identity between Nanopore- and Illumina-derived consensus sequences, with sequences from the same sample clustering together phylogenetically. This study establishes a sensitive, accurate, and cost-effective nanopore sequencing workflow for CVA6 genomic surveillance directly from clinical specimens. The workflow delivers clear, data-informed implementation guidance, making it a practical tool for outbreak response and evolutionary tracking.

**IMPORTANCE:**

Coxsackievirus A6 (CVA6) has become a predominant cause of hand, foot, and mouth disease worldwide, necessitating rapid and accessible genomic surveillance. This study establishes a tiling amplicon-based nanopore sequencing protocol that delivers complete CVA6 genomes directly from clinical samples within hours. The workflow demonstrates high sensitivity across a wide range of Ct values, achieves >99.8% consensus accuracy compared to Illumina sequencing, and offers clear, data-driven guidelines for implementation. By enabling same-day, cost-effective genomic analysis, this approach equips public health laboratories with a practical tool for real-time outbreak response and tracking of viral evolution.

## INTRODUCTION

**Fig 1:**
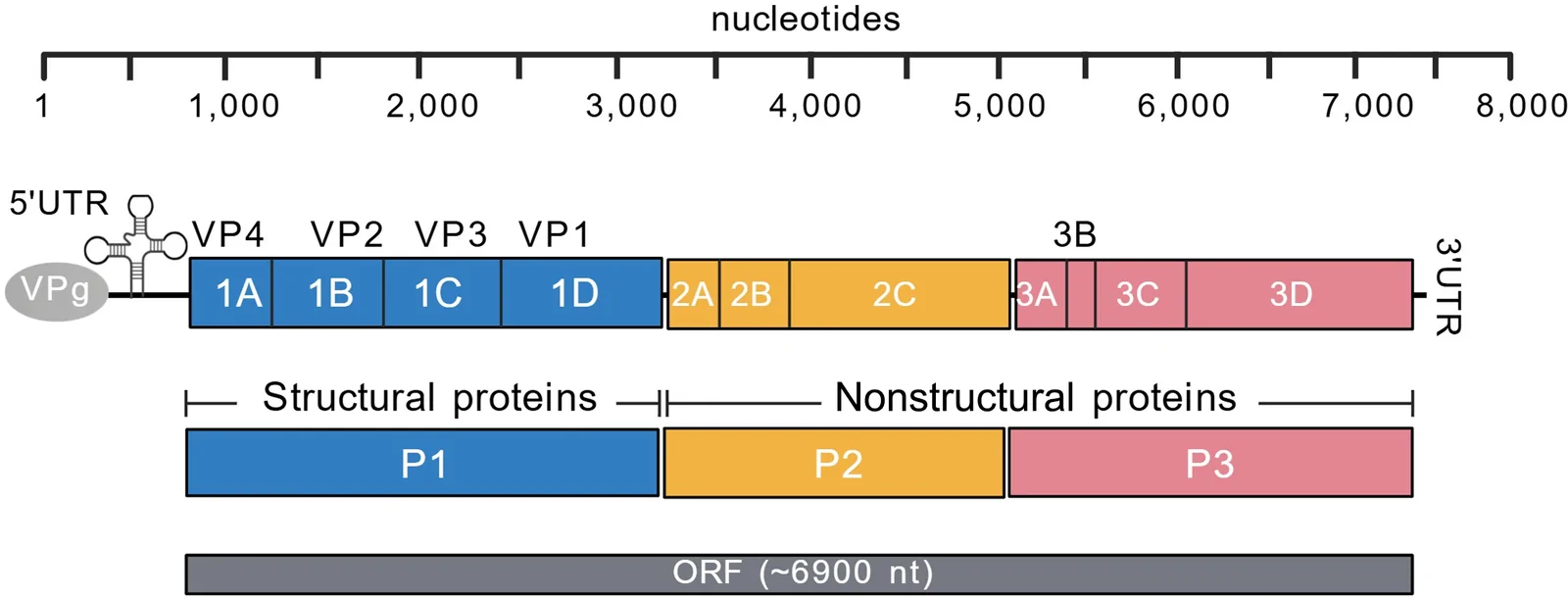
Diagram of genome organization of enteroviruses.

Hand, foot, and mouth disease (HFMD), which is caused by non-polio enteroviruses (EVs), has been prevalent worldwide for decades (1–3). In general, HFMD mainly occurs in children younger than 5 years old and only demonstrates benign symptoms such as rashes on the hands, feet, and in the mouth (4–8). The genus *Enterovirus* consists of 15 species: *Enterovirus alphacoxsackie*, *Enterovirus betacoxsackie*, *Enterovirus coxsackiepol*, *Enterovirus deconjuncti*, *Enterovirus eibovi*, *Enterovirus fitauri*, *Enterovirus geswini*, *Enterovirus hesimi*, *Enterovirus idromi*, *Enterovirus jesimi*, *Enterovirus krodeni*, *Enterovirus lesimi*, *Enterovirus alpharhino*, *Enterovirus betarhino*, and *Enterovirus cerhino.* These were formerly named *Enterovirus A-L* and *Rhinovirus A-C,* respectively (9). The enterovirus genome is approximately 7,500 nucleotides (nt) in length and consists of a positive, single-stranded RNA. It contains two untranslated regions (5′ and 3′-UTR) flanking a large open reading frame that encodes a polyprotein, which is subsequently cleaved into structural (1A–1D: VP4, VP2, VP3, and VP1) and non-structural proteins (2A–2C and 3A–3D) (Fig. 1) (4). The complete VP1 sequence effectively distinguishes human enterovirus serotypes, with intraserotypic divergence generally below 25% nucleotide difference, making it a valuable molecular basis for typing and taxonomy (10). HFMD is mainly associated with serotypes from *Enterovirus A* (EV-A) and *Enterovirus B* (EV-B) that contain 25 and 63 serotypes, respectively (6). There are various pathogens in EV-A associated with HFMD outbreaks, particularly EV-A71 and coxsackievirus A16 (CVA16) prior to 2012 (2, 11). Since then, CVA6, another serotype in EV-A, was responsible for a significant proportion of HFMD cases and outbreaks and turned out to be predominant over the past decade (12–14).

Prior to the first documented outbreak of atypical HFMD (aHFMD) in Finland in 2008 (15), CVA6 was of negligible clinical importance since most infections remained subclinical. Subsequently, the incidence of aHFMD, such as onychomadesis (nail shedding), extensive rashes, and ulceration, caused by CVA6 has escalated rapidly, with outbreaks occurring in France, Spain, Hungary, Japan, Singapore, the USA, the UK, and New Zealand (16–21). The rise of CVA6 has been particularly noticeable in China, according to previous reports (22–28). Genomic surveillance of CVA6 provides valuable information on its evolutionary dynamics, recombination, and spatiotemporal spreading (22). The development of the next-generation sequencing technique, to a great extent, promotes the genomic epidemiology of EVs over the past 20 years (29, 30). In recent years, the third-generation sequencing developed using the nanopore sequencing technique provides an alternative approach for genomic study (31).

The nanopore sequencing technology from Oxford Nanopore Technologies represents a significant advancement in portable sequencing technology, with applications that extend from diagnosis to public health surveillance and outbreak investigations (32). A large number of single nanopores are arranged on individual synthetic polymer membranes within a single flow cell. The migration of nucleic acid molecules loaded onto the flow cell is facilitated by their movement through nanopores, which follows an electric potential (33). It has been demonstrated that for amplicon sequencing applications, the cost of sequencing can be reduced by multiplexing several samples together by adding barcodes specifically to libraries of each sample (34). The utilization of nanopore sequencing in the context of viral amplicon sequencing has been previously documented (35, 36). Standard flow cells have been shown to achieve a total output of 10–20 Gb of data within 48 h. Furthermore, these flow cells are capable of attaining sufficient sequencing depth within a brief period of time, typically a few minutes, thereby producing a consensus sequence with a high degree of accuracy, exceeding 99% nucleotide identity to its Sanger-sequenced counterpart (37).

The goal of this study was to establish an amplicon sequencing protocol for CVA6 directly from clinical samples of HFMD cases using nanopore sequencing platforms. The sensitivity was determined by 10-fold serial dilution of CVA6-positive clinical samples, and its accuracy was compared to short-read (Illumina) sequencing. Furthermore, the established turnover time, data volume, and cost of the assays are to be considered in order to provide preliminary insights on the potential clinical applications of nanopore sequencing in the context of genotyping and genomic epidemiology of EVs.

## MATERIALS AND METHODS

### Molecular surveillance of enteroviruses and sample selection

Clinical samples (throat swabs or feces) collected from HFMD sentinel hospitals were sent to Nanchang Center for Disease Control and Prevention (NCCDC) for enterovirus diagnostics according to the molecular surveillance protocol (13). Swabs were stored in a dedicated Universal Transport Medium (Yocon, Beijing, China) for transport. Feces were diluted to a 10% suspension using minimum essential medias. After thorough mixing, 200 μL of each clinical sample was used for RNA extraction with the QIAamp Viral RNA Mini Kit (Qiagen, CA, USA) according to the manufacturer’s instructions. Genotypic identification was conducted using commercial real-time RT-PCR kits as previously reported (38). The major circulating serotypes CVA6, CVA10, CVA16, and EV-A71 were confirmed using commercial real-time RT-PCR kits (A2613YH-50T and A2622YH-50T, XABT-biotech, Beijing, China). Semi-quantitative data (cycle threshold [Ct]) were generated by the CVA6-specific real-time RT-PCR kit (A2651, XABT-biotech, Beijing, China). Eight CVA6-positive samples (five throat swabs and three feces) identified during 2020–2023 were randomly selected for serial dilution and the following experiments.

### cDNA synthesis

A total of 200 μL of clinical samples was used to extract RNA using the QIAamp Viral RNA Mini Kit (Qiagen, CA, USA). SuperScript III First-Strand Synthesis SuperMix (Invitrogen, USA) was used for cDNA synthesis. Specifically, all components were mixed and briefly centrifuged prior to use, and the thermal cycler was preheated to 65°C. The following components were combined in a 0.2 mL thin-walled PCR tube on ice: up to 5 μg of total RNA, 1 μL of 50 μM oligo(dT), 1 μL of annealing buffer, and RNase/DNase-free water to a final volume of 8 μL. The mixture was incubated in a thermal cycler at 65°C for 5 mins and then immediately placed on ice for at least 1 min. The contents of the tube were collected by brief centrifugation. Subsequently, 10 μL of 2× First-Strand Reaction Mix and 2 μL of SuperScript III/RNaseOUT Enzyme Mix were added to the tube on ice. Incubation was carried out as follows: 5–10 mins at 25°C, followed by 50 mins at 50°C. The reactions were terminated by incubation at 85°C for 5 mins.

### Design of tiling primers

Fifty complete CVA6 genomes from strains circulating in China between 2015 and 2023 were retrieved from the NCBI database, with genome lengths ranging from 7,358 to 7,476 nucleotides (Table S1). The sequence alignment was performed using MAFFT v7.400 (39), followed by initial primer design with PrimalScheme v3.2.3 (https://primalscheme.com/). Subsequently, each primer was manually reviewed and optimized to achieve >90% coverage. The primer pool ratios were optimized following sequencing to achieve balanced amplification, aiming for a maximum fivefold difference in coverage depth across amplicons. The tiling primers were distributed into two pools for targeted sequence capture of CVA6, and the finalized primer ratios are presented in Table S2.

### Nanopore library preparation and sequencing

Whole-genome sequencing of the CVA6 isolates was carried out using next-generation sequencing on the Nanopore GridION and PromethION platform with the established workflow (Fig. S1). The whole-genome amplification of CVA6 was performed using the Target Capture Kit for EV Whole Genome (BK-EV024, Baiyi Technology, Hangzhou, China) in Veriti Thermal Cycler (Thermo Fisher Scientific, USA). The purification of the DNA was then further refined through the use of Agencourt AMPure XP beads (Beckman Coulter, Brea, CA, USA). The Qubit DNA High Sensitivity (HS) kit was utilized to quantify the eluted DNA in the Qubit 4 fluorometer (Thermo Fisher Scientific, USA). The construction of the DNA library was performed with the LSK114.24 kit (Oxford Nanopore Technologies, UK) and the Multiple Samples DNA Library Prep Kit for Ligation Sequencing (BK-AUX024, Baiyi Technology, Hangzhou, China). First, DNA underwent end-repair and A-tailing. This was followed by barcode ligation using TA ligase, and finally, sequencing adapters were ligated using a rapid DNA ligase. Multiplex sequencing libraries were prepared using 250 ng of DNA from the 24 samples as input to the SQK-LSK110 kit and barcoded individually using the EXP-NBD104 Native barcodes (Oxford Nanopore Technologies, UK) and a modified One-pot protocol (40). All the libraries were divided into two equal parts and subjected to sequencing using MinION Flow Cell (R10.4.1) and PromethION flow cell (R10.4.1) on the GridION and PromethION 2 Solo device, respectively (Oxford Nanopore Technologies). The MinION Flow Cell on the GridION platform can produce 300 Mb data per hour, with measurements taken at every 5-min intervals. The PromethION flow cell on the PromethION platform can obtain seven times as much as that of the MinION flow cell on the GridION platform under the same conditions. We compared the two sequencing strategies to measure how quickly each could generate the data volume required to achieve 100% genome coverage of CVA6.

### Illumina sequencing

Nucleic acid enrichment and capture PCR were conducted for each cDNA sample using a Tarich Enterovirus Genome Enrichment Kit (BioGerm, China) in strict accordance with the manufacturer’s instructions. The amplified products were purified using the QIAquick PCR Purification Kit (Qiagen, Germany). The amplification products were mixed at equal concentrations and subsequently sheared into fragments of approximately 200 bp using a Covaris instrument (Covaris Inc., USA). The construction of the DNA library was carried out in strict accordance with the protocols outlined in the Nextera XT DNA Library Preparation Kit (Illumina, USA). Each DNA library was labeled with a distinct barcode to differentiate the samples utilized for Illumina sequencing. The DNA libraries were measured using Qubit DNA High Sensitivity (HS) kit in Qubit 4 fluorometer (Thermo Fisher Scientific, USA), and each library was mixed together at the same concentration. The obtained DNA libraries were then subjected to qualitative assessment using an Agilent 2100 Bioanalyzer (Agilent, USA). This instrument employs microfluidic electrophoresis to analyze the fragment size distribution and quality of DNA libraries and was used here to verify that the library fragment sizes were as expected and to assess overall library quality. Following this assessment, the libraries were loaded onto the Illumina NovaSeq System (Illumina, USA).

### Bioinformatic analysis of nanopore sequencing data

The base calling and demultiplexing of nanopore reads were performed using Guppy with default parameters (Oxford Nanopore Technologies, Oxford, UK). The output fastq files were passed through Porechop v0.2.3 (https://github.com/rrwick/Porechop) to remove the cross-linked sequences using the parameter “discard_middle.” The BAIYI MicroGeno Platform (v5.0, Hangzhou Baiyi Technology Co., Ltd., http://www.baiyi-tech.cn/) was utilized for integrated analysis. Raw data were filtered and trimmed by fastp (v1.0) (41) and quality controlled by fastQC (v0.12.0) (42). The Kraken2 (v2.1.2) (43) was utilized to ascertain the presence of the target virus sequence and to calculate its sequence count and proportion. Furthermore, the aligned target enterovirus sequence information was extracted by using samtools (v1.15, https://github.com/samtools/samtools). The *de novo* assembly was performed by Flye (v2.9.6) (44) and Raven (v1.8) (45), and then the contigs were blasted against the NCBI database (core_nt) to identify other members of *Enterovirus A*. The clean data were aligned against enterovirus reference genomes obtained from the NCBI website (https://www.ncbi.nlm.nih.gov/) using Minimap2 (v2.30) (46) for genome coverage and sequencing depth comparison. The reference genome with the highest genome coverage and sequencing depth of the aligned results was considered the optimal reference genome, and the complete genome sequence of CVA6 was subsequently assembled using Bcftools (v1.12) based on the optimal reference genome (47). The sequence alignments were performed using MUSCLE (v5.3) (48). A neighbor-joining (NJ) (49) phylogeny was generated for genome sequences of 24 libraries and 50 references using Mega 12 (50), in which the Tamura-Nei method (51) of evolutionary distances and a gamma-distributed rate variation among sites were applied.

### Bioinformatic analysis of Illumina sequencing data

Raw sequencing data were first assessed using FastQC (v0.12.0) and then processed with fastp (v1.0) to remove adapters and low-quality reads. Cleaned reads were aligned to the reference genome (Table S5) using BWA (v0.7.17), and sorted/indexed BAM files were generated with SAMtools (v1.16.1). Consensus sequences were called using iVar (v1.3.1) (52), and mean sequencing depth was calculated (Table S5). In cases where genomic regions remained uncovered during assembly or where sequence discrepancies were exceptionally high, primers were redesigned to target these gaps or the complete genome, followed by Sanger sequencing to validate and complete the genome sequences, thereby ensuring both completeness and accuracy of the final assemblies. The genomes obtained through second-generation and third-generation sequencing were pairwise aligned and calculated using the tool distance in Mega 12.

## RESULTS

### Sample preparation and serial dilution

**Fig 2:**
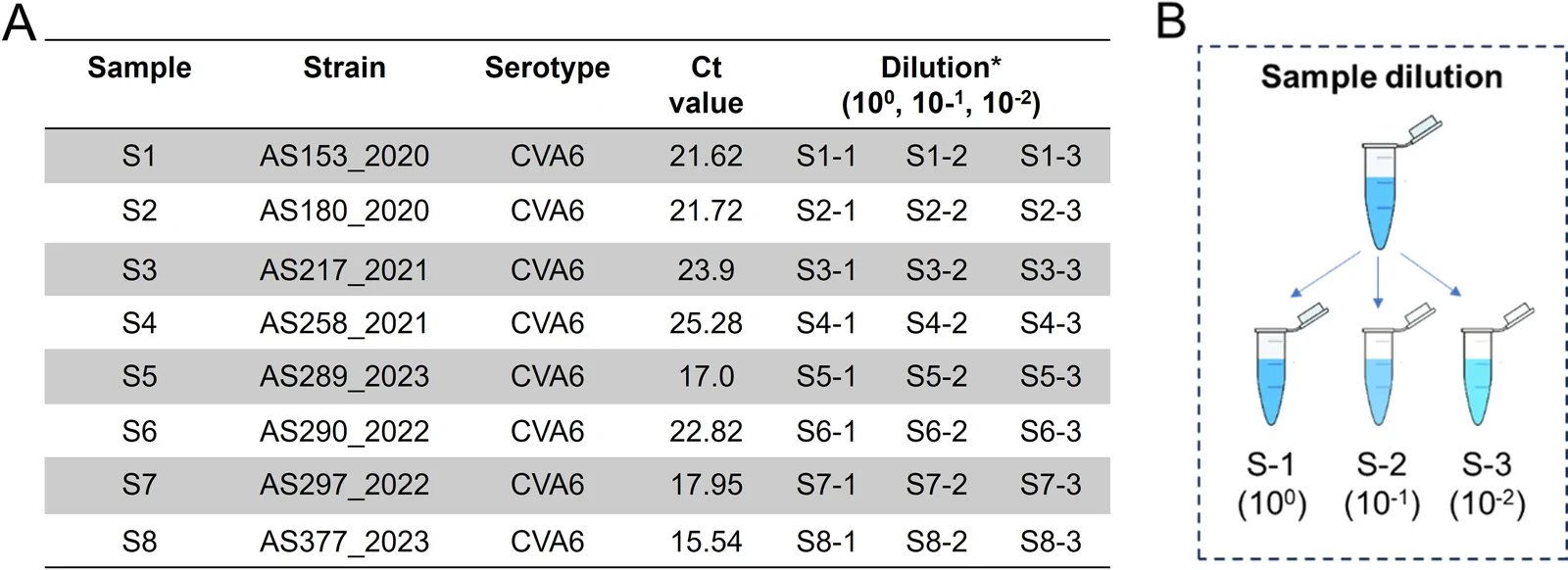
Serial dilution of CVA6-positive clinical samples. (**A**) Ct values of eight (S1–S8) CVA6-positive clinical specimens. (**B**) Flowchart of serial dilution. *A serial 10-fold dilution series was prepared from each original clinical specimen. Briefly, 500 µL of the thoroughly vortexed original sample was aliquoted into a new microcentrifuge tube to prepare the 10^0^ (undiluted) sample. To prepare the 10^−1^ dilution, 100 µL of this aliquot was transferred into 900 µL of phosphate-buffered saline (PBS) and mixed thoroughly. Subsequently, 100 µL of the resulting 10^−1^ dilution was transferred into a fresh tube containing 900 µL of PBS to yield the 10^−2^ dilution. This process generated three concentration levels (10^0^, 10^−1^, and 10^−2^) for each of the eight CVA6-positive samples, resulting in a total of 24 libraries for sequencing analysis.

As detailed in Materials and Methods, eight CVA6-positive samples were randomly selected from HFMD cases reported between 2020 and 2023. Viral RNA was semi-quantified using real-time RT-PCR, yielding Ct values ranging from 15.54 to 25.28 (Fig. 2A). Each of these samples was subjected to a serial 10-fold dilution series (undiluted, 10⁻¹, and 10⁻²), resulting in a total of 24 libraries (Fig. 2B). These libraries were used to establish and validate the CVA6 nanopore sequencing protocol.

### Library construction and quantification

**Fig 3:**
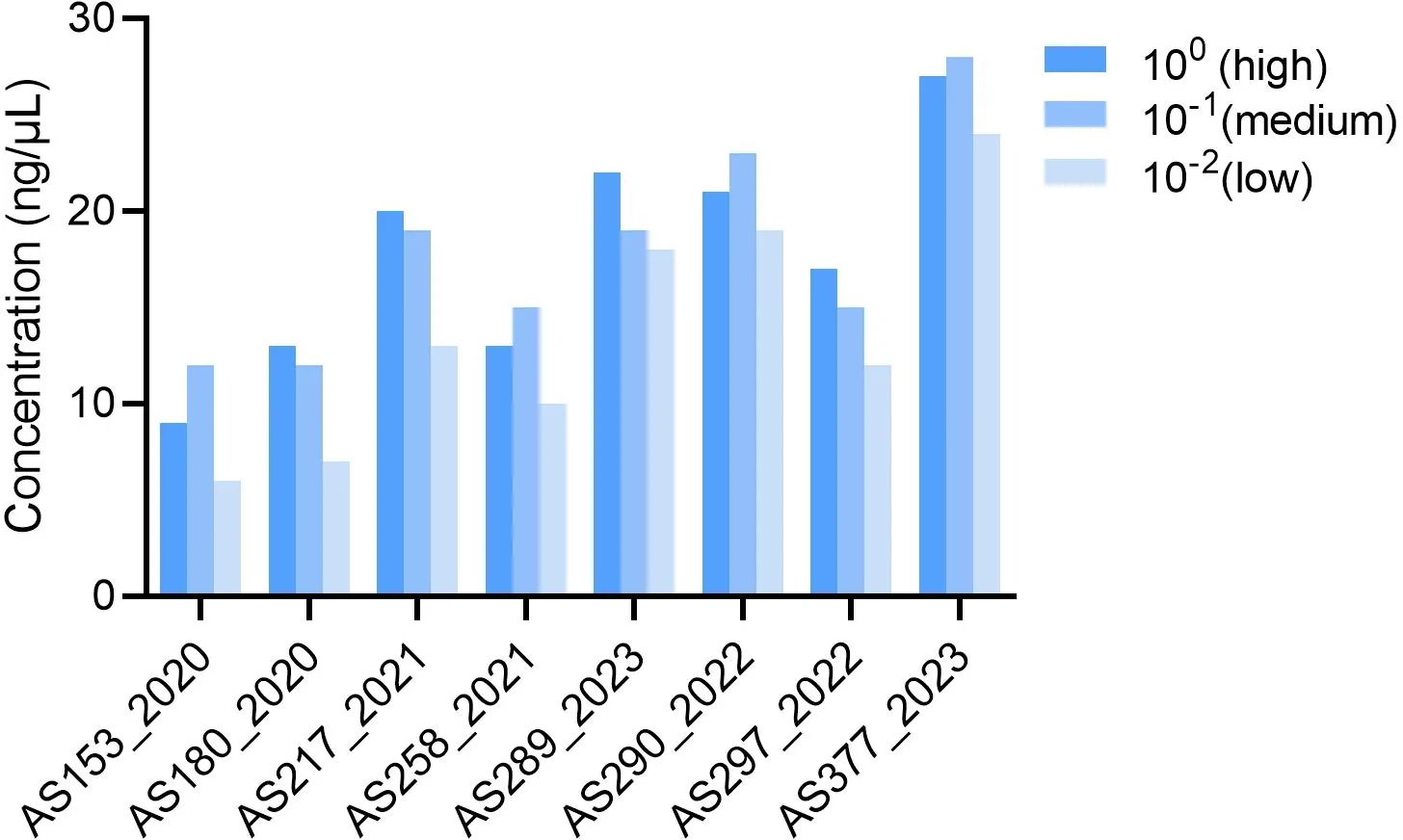
The concentrations of the pooled PCR products from the 24 samples.

A set of 11 overlapping primer pairs, designed to target conserved regions across 50 CVA6 strains circulating in China (2015–2023), was divided into two multiplex pools (Table S2). For each of the 24 libraries, cDNA—synthesized from viral RNA as described in Materials and Methods—was independently amplified in two parallel PCRs using primer pool 1 and primer pool 2, respectively (Tables S3 and S4), following the workflow in Fig. S1. The amplicons generated from both pools for each library were then combined equimolarly. The concentration of each pooled PCR product was quantified using a Qubit fluorometer (Fig. 3). Prior to sequencing, unique barcodes were ligated to individual libraries to enable multiplexed sample identification during data analysis.

### Depth of genome coverage

**Fig 4:**
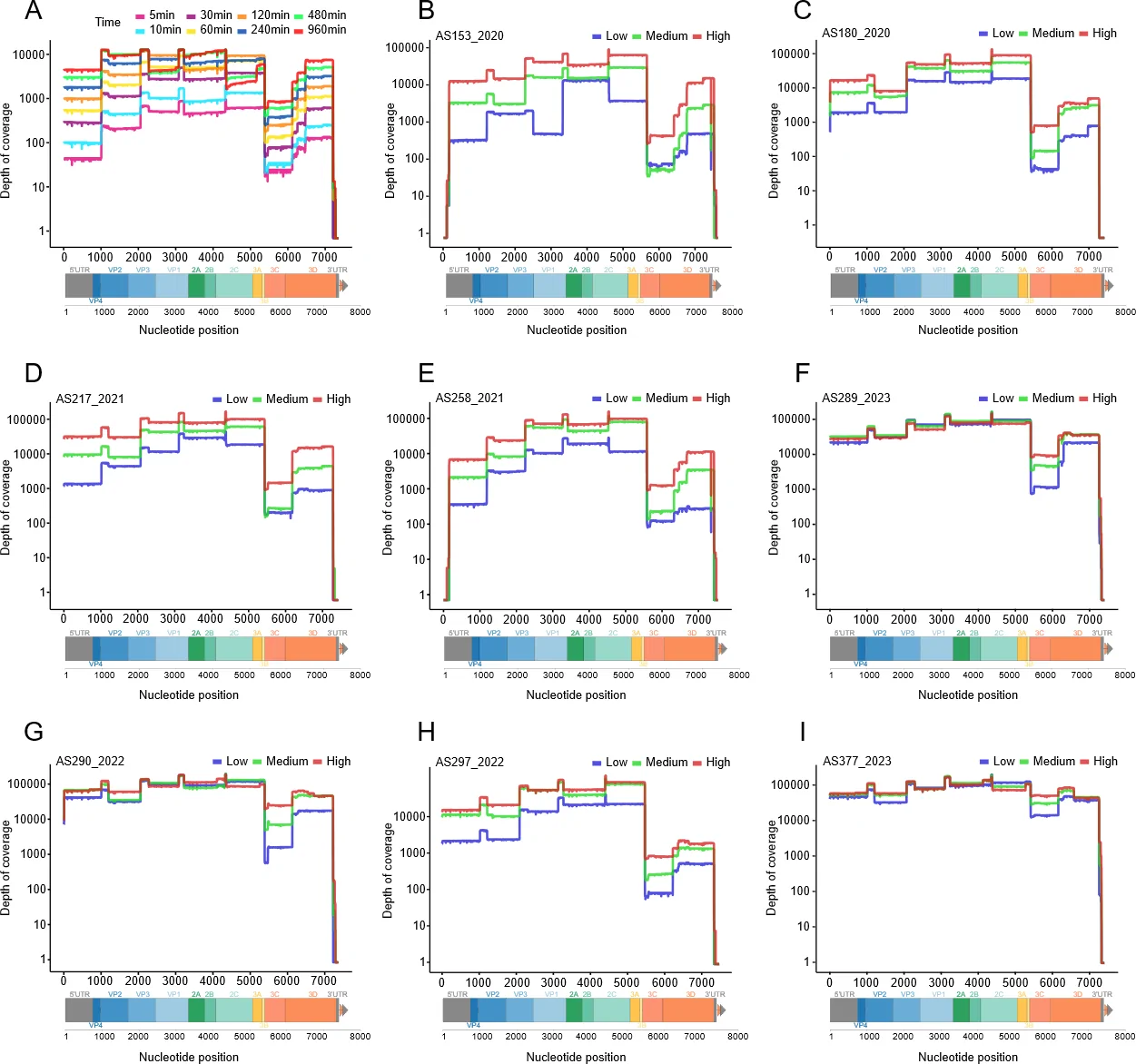
The depth of genomic coverage for CVA6 with time, across concentrations, and among samples using PromethION flow cell. Panel **A** shows the trends of the average depth data of genomic coverage for all samples in 16 h. Panels **B–I** show the entire depth data of genomic coverage for the eight clinical specimens with three serial dilutions (low, 10^−2^; medium, 10^−1^; and high, 10^0^). 5′ UTR, translation and replication control (1–743 nt); VP4, mediates viral uncoating (744–950 nt); VP2, maintains capsid stability (951–1,709 nt); VP3, assists cell attachment (1,710–2,441 nt); VP1, attachment and neutralizing antigen (2,442–3,335 nt); 2A, cleavage and translation hijack (3,336–3,779 nt); 2B, modulates membrane permeability (3,780–4,079 nt); 2C, RNA helicase and replication (4,080–5,072 nt); 3A, replication membrane scaffold (5,073–5,336 nt); 3B (VPg), replication primer (5,337–5,408 nt); 3C, polyprotein processing (5,409–5,957 nt); 3D (RdRp), RNA-dependent RNA polymerase (5,958–7,340 nt); and 3′ UTR, RNA stability and replication (7,341–7,411 nt).

The 24 libraries, derived from serial dilutions of eight CVA6-positive samples, covered a wide range of Ct values (15.54–32.7) (Table 1). All libraries were sequenced on the PromethION flow cell using the established tiling amplicon workflow. The protocol demonstrated high efficiency and speed, achieving an average sequencing depth of >10× within 5 mins of run initiation (Fig. 4A). High genome coverage (≥95%) was consistently attained across all samples and dilution levels, with most libraries reaching ≥98.1% coverage (Table 1; Fig. 4B through I). Notably, for the majority of samples (6/8), the highest dilution (10^−2^) yielded coverage comparable to or, in several cases, slightly higher than that of the undiluted sample, confirming the robustness of the capture protocol across a wide range of template concentrations.

**TABLE 1:** Outline of the sequencing results of the 24 samples in PromethION

Sample no.	Dilution	Ct value	Reads	Yield (Mb)	CVA6 (Mb)	CVA6/ yield (%)	Genome coverage (%)
AS153_2020	10^−2^	28.7	229,672	137.1	13.5	9.8	96.5
	10^−1^	25.61	179,199	127.6	49.5	38.8	95.5
	10^0^	21.62	256,718	208.4	143.2	68.7	98.1
AS180_2020	10^−2^	28.09	214,741	143.3	42.3	29.5	98.1
	10^−1^	25.05	226,524	188.8	119.0	63.0	98.1
	10^0^	21.72	298,330	259.2	194.3	75.0	98.1
AS217_2021	10^−2^	30.42	233,299	156.2	37.9	24.2	99.1
	10^−1^	27.39	185,553	159.6	103.0	64.5	98.1
	10^0^	23.9	274,764	261.7	212.5	81.2	98.1
AS258_2021	10^−2^	32.7	150,718	102.7	20.7	20.1	96.5
	10^−1^	29.11	246,035	191.0	98.9	51.8	96.5
	10^0^	25.28	205,093	187.9	145.2	77.3	98.1
AS289_2023	10^−2^	24.21	201,595	200.0	168.1	84.0	98.2
	10^−1^	21.39	194,874	205.5	187.7	91.3	98.7
	10^0^	17.95	183,253	187.3	165.0	88.1	98.1
AS290_2022	10^−2^	23.69	200,791	202.6	174.6	86.2	98.1
	10^−1^	20.52	217,772	225.2	207.1	91.9	100.0
	10^0^	17.0	204,679	226.3	214.8	94.9	98.7
AS297_2022	10^−2^	29.77	179,801	117.6	22.6	19.2	96.5
	10^−1^	26.29	215,034	177.5	75.7	42.7	98.1
	10^0^	22.82	198,867	176.6	92.0	52.1	98.1
AS377_2023	10^−2^	21.73	167,042	176.5	161.4	91.4	98.7
	10^−1^	18.52	174,260	192.8	180.4	93.6	98.9
	10^0^	15.54	184,640	200.5	181.6	90.6	98.9

### Data volume and target reads ratio

**Fig 5:**
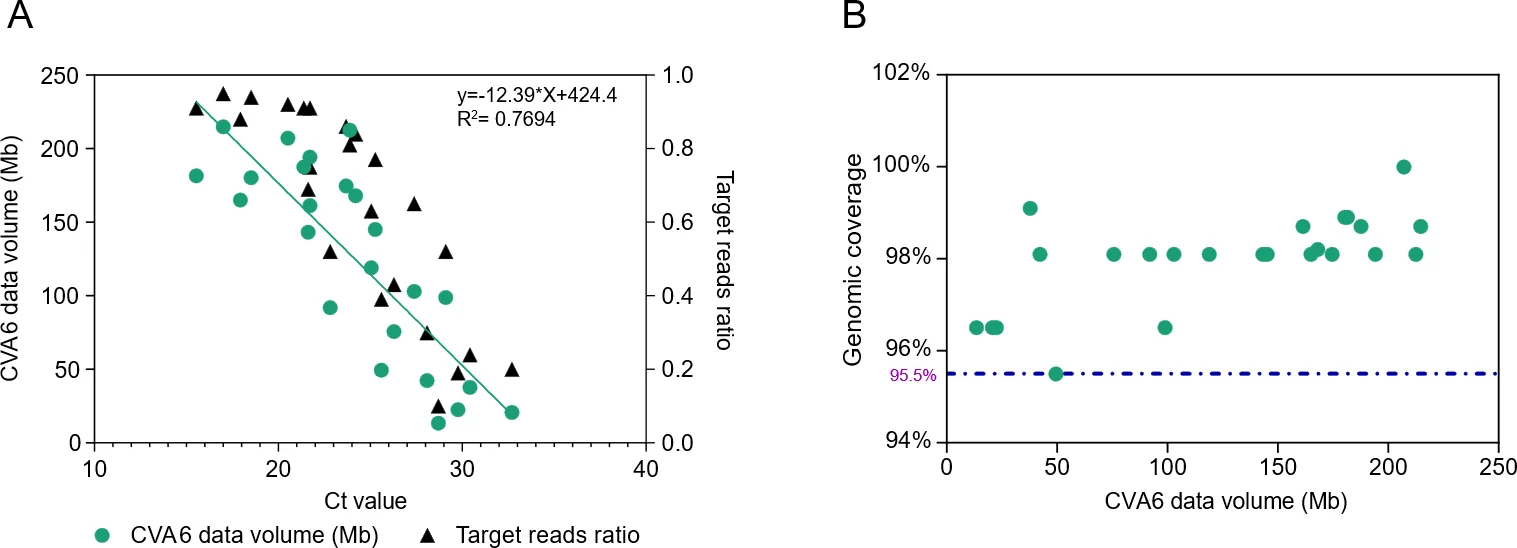
The relevance between data volume, target reads ratio, genomic coverage, and Ct value of the CVA6 genome. Panel **A** shows the plot of Ct values fitted to the CVA6 data volume and the target reads ratio. Panel **B** shows the scatter plot of CVA6 data volume versus genomic coverage. The blue dotted line represents the lowest genomic coverage in this study.

The relationship between Ct value, CVA6 data volume, target reads ratio, and genomic coverage was analyzed. As shown in Fig. 5A, a lower Ct value, suggesting higher viral load, was associated with a trend of higher CVA6 data volume yield and a higher target reads ratio. Despite this variation in enrichment efficiency, achieving complete genomic coverage depended primarily on obtaining a sufficient absolute yield of CVA6-specific reads. Although genome coverage reached 95.5%–100% with a wide range of CVA6 data volumes (Fig. 5B), most of the highest coverage levels were observed at higher CVA6 data volumes, suggesting a trend between CVA6 data volumes and genomic coverage. In this data set, a CVA6-specific data volume of approximately 10 Mb was already sufficient to achieve high genome coverage (>95%) for most samples (Fig. 5B). These empirical observations from our experiments indicate that the capture protocol is highly efficient, and that even relatively modest CVA6 yields can produce near-complete genomes.

### Comparative performance of PromethION and MinION flow cells

To evaluate the performance of different nanopore platforms for this targeted assay, we sequenced the same set of 24 CVA6 libraries (derived from eight samples at three dilution levels) using both MinION (in GridION sequencer) and PromethION flow cells. The PromethION flow cell demonstrated a higher overall sequencing throughput and yielded a greater total output within a comparable time frame (Fig. S2). While both platforms successfully generated complete CVA6 genomes, the higher throughput of the PromethION flow cell makes it more suitable for laboratories aiming to maximize sample multiplexing in a single run. The choice between MinION and PromethION flow cells for this assay can be guided by the laboratory’s throughput requirements and existing instrumentation.

### High concordance of CVA6 consensus genomes generated by Illumina and nanopore sequencing

**Fig 6:**
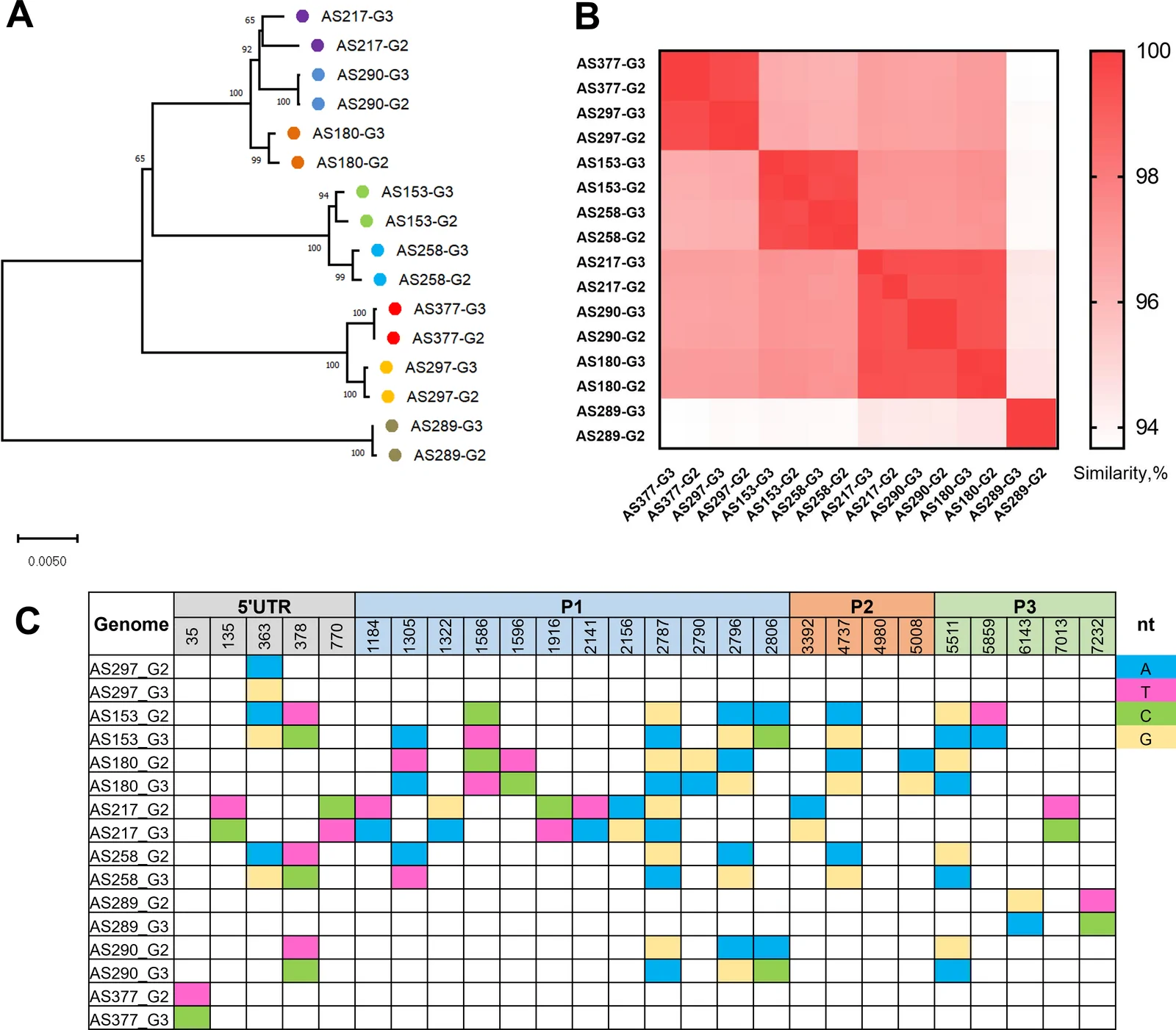
Comparative analysis of CVA6 genomes obtained using Nanopore and Illumina sequencing techniques. (**A**) NJ tree of CVA6 strains of this study. (**B**) Pairwise analysis of CVA6 genome sequences using Nanopore and Illumina sequencing techniques. (**C**) Single nucleotide polymorphism of consensus sequences of both sequencing techniques. G2, Illumina sequencing; G3, Nanopore sequencing of the undiluted clinical samples. Scale bar, nucleotide substitutions/site.

To evaluate the accuracy of our nanopore sequencing workflow, we sequenced eight undiluted CVA6-positive clinical samples on both Illumina and Nanopore platforms. The resulting consensus genomes were compared using pairwise alignment and phylogenetic analysis (Fig. 6A and B). A neighbor-joining tree was constructed based on the 16 consensus sequences (8 samples × 2 platforms) to assess intra-sample clustering (Fig. 6A). The tree clearly shows that sequences from the same sample consistently grouped together, regardless of sequencing platform. This indicates that the assay preserves true phylogenetic relationships. A strikingly low genome identity was observed for strain AS289 compared to the other seven strains. Recombination analysis using Simplot (v3.5.1) was conducted, and no recombination signal was detected. Moreover, phylogenetic trees built using P1, P2, P3, and the genome supported the finding (Fig. S3). The sequence discrepancies between AS289 and the other seven samples were predominantly concentrated in P3 regions, as the phylogenetic trees indicated, highlighting the necessity of constant genomic surveillance of CVA6.

Alignment of the consensus sequences identified a small number of nucleotide differences between platforms. These discrepancies were primarily concentrated in VP1–VP3 regions (nucleotides 951–3,335) (Fig. 6C). Pairwise identity between consensus genomes obtained from the same sample by Nanopore and Illumina sequencing ranged from 99.81% to 99.98% (Fig. 6B), suggesting the reliability and accuracy of application of nanopore sequencing directly from clinical samples of HFMD cases.

### Consistency of consensus sequences across dilution series

**Fig 7:**
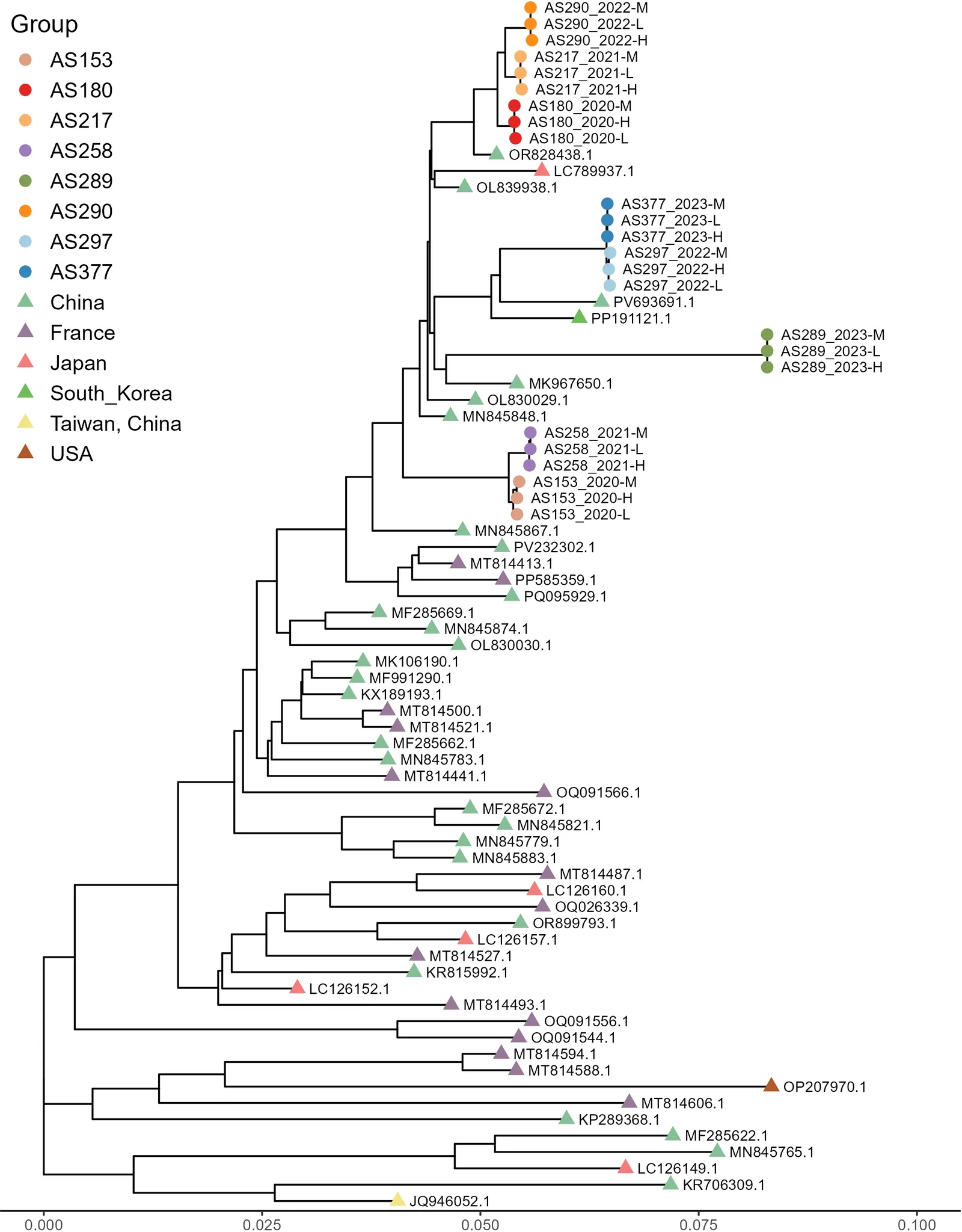
Near full-length genome-based phylogenetic analysis. The neighbor-joining tree was built using genome sequences of 24 libraries (●) and 50 references (▲). Scale bar, nucleotide substitutions/site; L, low (10^−2^); M, medium (10^−1^); and H, high (10^0^, undiluted).

The robustness of the sequencing protocol was further evaluated by examining genetic consistency across serial dilutions of the same sample. Consensus sequences derived from different dilution levels of each specimen showed remarkably high concordance. For all samples except AS153, only one single nucleotide polymorphism (SNP) was detected between the three dilution gradients. Sample AS153 exhibited slightly greater variation, with five SNPs identified across its dilution series. Overall, pairwise sequence identities within samples ranged from 99.96% to 100%, and sequences from the same sample consistently clustered together in the phylogenetic tree (Fig. 7). These results indicate that the sequencing protocol yields reproducible and accurate consensus genomes across a wide range of Ct values, with negligible dilution-induced sequence variation.

## DISCUSSION

The global emergence of CVA6 as a predominant and clinically significant cause of HFMD outbreaks highlights an urgent and growing need for accessible, rapid, and accurate genomic surveillance tools that can be deployed directly in public health laboratories (4, 22). This study establishes and validates a targeted amplicon-based nanopore sequencing workflow for complete CVA6 genome sequencing, demonstrating high sensitivity, accuracy, and operational flexibility directly from clinical specimens. Our results demonstrate that this protocol combines high sensitivity, operational speed, and analytical accuracy, presenting a practical and robust solution for outbreak response and ongoing evolutionary tracking.

A fundamental aspect of our methodology entailed the formulation of a tiling primer scheme based on the circulating CVA6 strains in China. This scheme facilitated the effective amplification of near-full-length CVA6 genomes across a substantial spectrum of viral RNA concentrations. The protocol was found to be remarkably rapid, attaining >10× sequencing depth in 5 mins on a PromethION flow cell (Fig. 4A). A correlation was observed between Ct values and the absolute yield of CVA6-specific data (Fig. 5). Furthermore, a trend toward higher genome coverage was noted with increasing CVA6 data volumes. It may be possible to determine minimum sequencing data volume requirements by using Ct values alone or in combination with high-throughput sequencing metrics.

The accuracy of the consensus genomes generated by our nanopore workflow was rigorously benchmarked against the gold-standard Illumina short-read sequencing. Comparative analysis revealed a very high degree of concordance, with identity between platform-derived consensus sequences ranging from 99.81% to 99.98% (Fig. 6B). Most importantly, phylogenetic analysis demonstrated that sequences from the same clinical sample consistently formed monophyletic clusters, irrespective of the sequencing technology used (Fig. 6A). This finding provides strong evidence that the assay preserves the true phylogenetic signal, a prerequisite for reliable genotyping, outbreak linkage, and evolutionary studies. The few observed nucleotide discrepancies were predominantly located within the VP1–VP3 regions (Fig. 6C), corresponding to known mutation hotspots in the capsid proteins of enteroviruses (53). Therefore, the minor inconsistencies are more likely attributable to the combined challenges of amplifying and unambiguously mapping this highly variable region with a fixed primer set, rather than to a systematic error of the nanopore platform.

Our evaluation of sequencing platforms confirms the assay’s flexibility. Both MinION (operated on a GridION device) and PromethION flow cells (500 versus 3,000 pores) successfully generated complete CVA6 genomes from all multiplexed libraries. The primary practical difference lies in throughput; the PromethION flow cell, with its greater number of pores, provides higher total data output per run (Fig. S2), making it more suitable for laboratories aiming to maximize sample multiplexing. The choice between platforms can therefore be guided by the existing laboratory infrastructure and the desired scale of operation, enhancing the method’s potential for widespread adoption.

Despite its strengths, several limitations of the current workflow should be acknowledged to guide future improvements. First, while the primer set was effective against the tested strains, its design, based on historical and regionally focused sequences, may not fully capture the global genetic diversity of CVA6, particularly novel recombinant variants. This is an inherent challenge in amplicon-based sequencing that necessitates a commitment to periodic primer scheme updates informed by contemporary surveillance data (54). In this study, we observed relatively low sequencing depth between nucleotides 5,000 and 7,500 (the 3A–3D region) (Fig. 4), a genomic region where recombination events occur frequently (16). This finding highlights the need for primer optimization for this specific region in future studies. Second, although PCR amplification is an indispensable step to achieve adequate analytical sensitivity for viral target detection in clinical specimens (with high background nucleic acid and low viral load), it introduces inherent technical limitations that fundamentally compromise the reliable detection and quantitative characterization of low-frequency minority variants in viral quasispecies (55, 56). Specifically, PCR amplification bias, stochastic sampling error in early amplification cycles, template competition between dominant and minor variants, and polymerase-introduced artifactual mutations can all lead to the loss of true low-frequency variants, false-positive variant calls, and severe distortion of variant allele frequency quantification (57, 58). For applications requiring haplotype resolution, metagenomic or direct RNA sequencing approaches remain necessary. Finally, while our sample size was sufficient for robust protocol validation, expanding evaluation to larger, geographically diverse cohorts will further establish the generalizability and performance boundaries of the assay.

In this study, we focused exclusively on CVA6-positive clinical samples. However, our primer design was based on conserved regions among multiple CVA6 strains, and sequence alignment suggests that several primer binding sites may also be conserved across other members of the *Enterovirus* A species. During library preparation, we occasionally detected low-abundance reads mapping to other EV-A serotypes such as EV-A71 (data not shown), indicating co-infection or suboptimal amplification of related viruses. While the current protocol was not formally validated for these targets, this incidental cross-reactivity suggests that the assay may be moderately promiscuous within EV-A. With minor primer adjustments or dedicated validation, the same tiling amplicon approach could be expanded to enable simultaneous or broad-spectrum genomic surveillance of multiple EV-A serotypes directly from clinical specimens.

In conclusion, this study establishes a sensitive, accurate, and operationally feasible nanopore sequencing workflow for the genomic surveillance of CVA6 directly from clinical samples. By delivering high-quality, complete genomes within a single day and providing clear implementation guidelines, this approach significantly lowers the technical and logistical barriers to real-time genomic epidemiology. The workflow’s demonstrated accuracy for consensus-level analysis and its adaptability across sequencing platforms make it a valuable tool for public health laboratories. Furthermore, the primer design strategy and methodological framework presented here could be readily adapted for the genomic surveillance of other clinically important enteroviruses and RNA viruses.

## Supplementary Material

Reviewer comments

## Data Availability

The genome sequences of CVA6 strains in this study were submitted to GenBank with accession numbers PQ458982, PQ458983, PQ458988, PQ458990, PQ458993, PQ458994, PQ458998, and PQ459000; the raw sequencing reads generated in this study have been deposited to SRA with BioProject accession number PRJNA1398593 (SRX31713286–SRX31713309).

## SUPPLEMENTAL MATERIAL

Supplemental material spectrum.03246-25-s0001.docx Fig. S1 to S3; Tables S1 to S5.

OPEN PEER REVIEW reviewer-comments.pdf An accounting of the reviewer comments and feedback.

ASM does not own the copyrights to Supplemental Material that may be linked to, or accessed through, an article. The authors have granted ASM a non-exclusive, world-wide license to publish the Supplemental Material files. Please contact the corresponding author directly for reuse.
