# Supplementary material for: Targeted sequence capture of coxsackievirus A6 using nanopore sequencing directly from clinical specimens
Source: Microbiol Spectr. 2026 Jun 4;14(7):e03246-25. doi: 10.1128/spectrum.03246-25 (PMC13340022; doi:10.1128/spectrum.03246-25)
Supplement: Supplemental material — Fig. S1 to S3; Tables S1 to S5. [file spectrum.03246-25-s0001.docx]

**Targeted sequence capture of coxsackievirus A6 using Nanopore sequencing directly from clinical specimens**

Ziqi Lin^1,2,3#^, Fenglan He^1#^, Han Mo^2,3#^, Lingfeng Mao^4^, Xingyu Xu^4^, Liu Yi^1^, Ke Qian^1^, Xiansheng Ni^1^, Tielong Xu^2^, Xianfeng Zhou^2, 3^*, Hui Li^1^*

1 Jiangxi Provincial Health Commission Key Laboratory of Pathogenic Diagnosis and Genomics of Emerging Infectious Diseases, Nanchang Center for Disease Control and Prevention, Nanchang 330008, China

2 Evidence-based Medicine Research Center, Jiangxi University of Chinese Medicine, Nanchang 330004, China

3 Mass Spectrometry Diagnostics and Chronic Disease Rehabilitation Research Center, Jiangxi University of Chinese Medicine, Nanchang 330004, China

4 Hangzhou Baiyi Biotechnology Co., Ltd., Hangzhou 310052, China.

# These authors contribute equally

*Correspondence to: X. Zhou (zhouxianfeng@jxutcm.edu.cn) and H. Li (nccdclih@163.com)

**Supplementary Figure S1 Workflow of Nanopore sequencing of CVA6 directly from clinical samples.** Clinical sample collection (yellow), genotypic identification (green), sample processing and sequencing using Oxford Nanopore Technologies (blue), and processing of sequence data (gray)


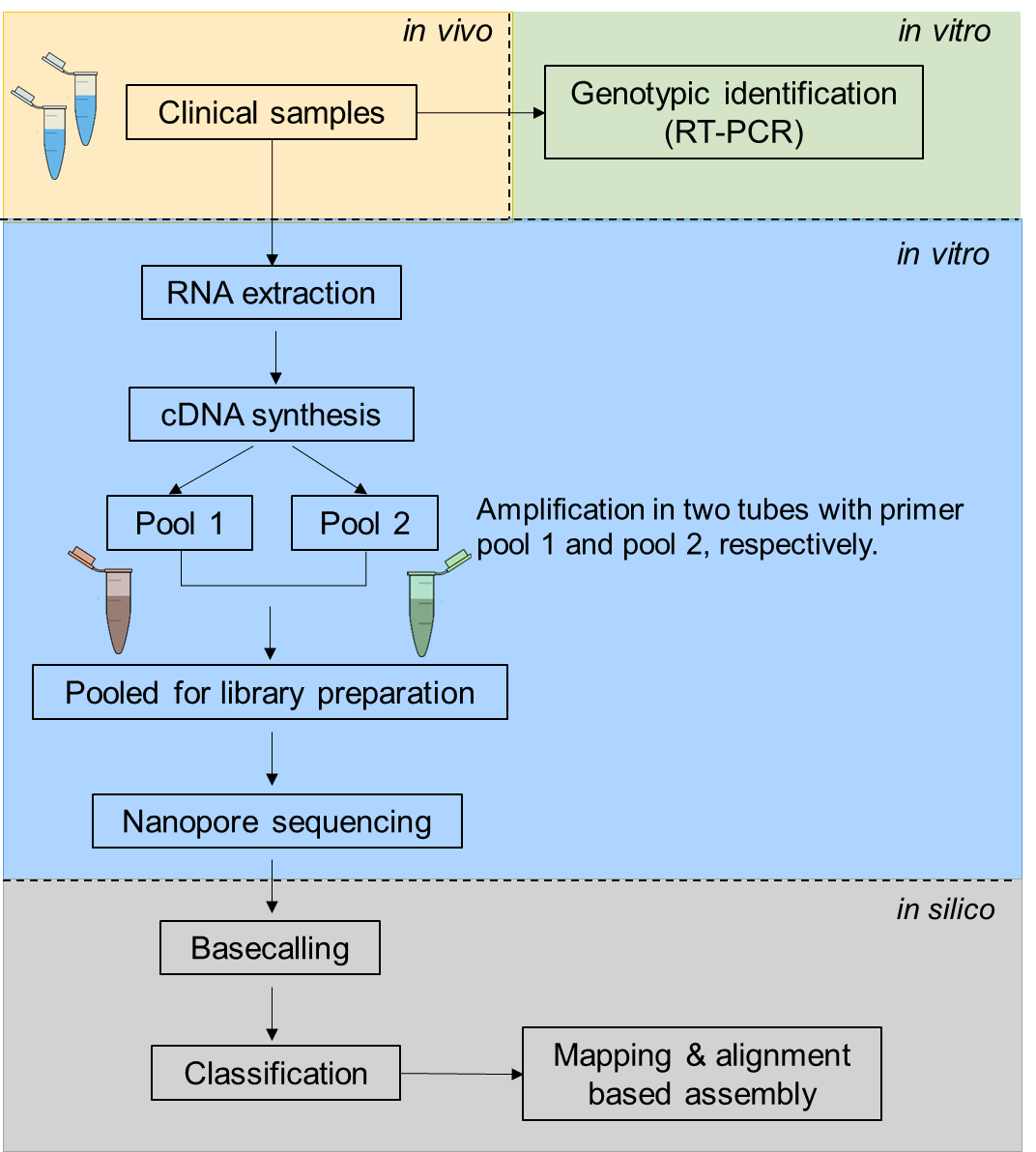


**Protocol of Nanopore sequencing**

**Step 1 Genotypic identification from clinical samples of HFMD cases**

Clinical samples (throat/anal swabs or feces) collected from HFMD sentinel hospitals were sent to molecular surveillance network laboratory for enterovirus diagnostics. Swabs were stored in dedicated Universal Transport Medium (UTM) (Yocon, Beijing, China) for transport. Feces were diluted to a 10% suspension using MEMs. The circulating viruses CVA6, CVA10, CVA16 and EV-A71 were confirmed using commercial real time RT-PCR kits (A2613YH-50T and A2613YH-50T, XABT-biotech, Beijing, China). Quantitative data (cycle threshold [Ct]) were generated by the CVA6-specific TaqMan qRT-PCR kit (A2651, XABT-biotech, Beijing, China).

**Step 2 Library preparation and sequencing**

The selected CVA6-positive samples were subjected to nanopore sequencing. First, A total of 200 μl of clinical samples were used to extract RNA using the QIAamp Viral RNA Mini Kit (Qiagen, CA). SuperScript™ III First-Strand Synthesis SuperMix (Invitrogen, USA) was used for cDNA synthesis. Then the whole genome amplification of CVA6 was performed using the Target Capture Kit for EV Whole Genome (BK-EV024, Baiyi Technology, Hangzhou, China) in Veriti Thermal Cycler (Thermo Fisher Scientific, USA. The tiling primers for targeted sequence capture of CVA6 were divided into two pools (Supplementary Table S2). And the PCR reaction component and program were detailed in Supplementary Table S3 and S4. The PCR products were pooled for library preparation. Multiplex sequencing libraries were prepared using 250 ng of DNA from the 24 samples as input to the SQK-LSK110 kit and barcoded individually using the EXP-NBD104 Native barcodes (Oxford Nanopore Technologies, UK). The libraries were subjected to sequencing using MinION flow cells and PromethION flow cells on the GridION and PromethION 2 Solo device, respectively (Oxford Nanopore Technologies, UK).

**Step 3 Basecalling and bioinformatic analysis**

Guppy was used for base calling and initial demultiplexing of nanopore reads. Demultiplexing was refined using Porechop. The BAIYI MicroGeno Platform, fastp, and fastQC performed integrated analysis, filtering, and quality control. Kraken2 identified target virus sequences, and samtools extracted aligned enterovirus reads. De-novo assembly was conducted with Flye and Raven, with contigs blasted against the NCBI database. Minimap2 compared data to NCBI enterovirus references to select an optimal genome. Freebayes and Bcftools were used to identify and assemble the complete genome. MUSCLE performed sequence alignment, and RAxML generated a maximum likelihood phylogeny from the near full-length genome.

**Supplementary Figure S2 The cumulative sequencing yield (total data generated) for all 24 samples over time on each platform.** A. the scatters plot of the data volumes against time on PromethION sequencer using PromethION flow cell. B. the scatters plot of data volumes against time on GridION sequencer using MinION flow cell.


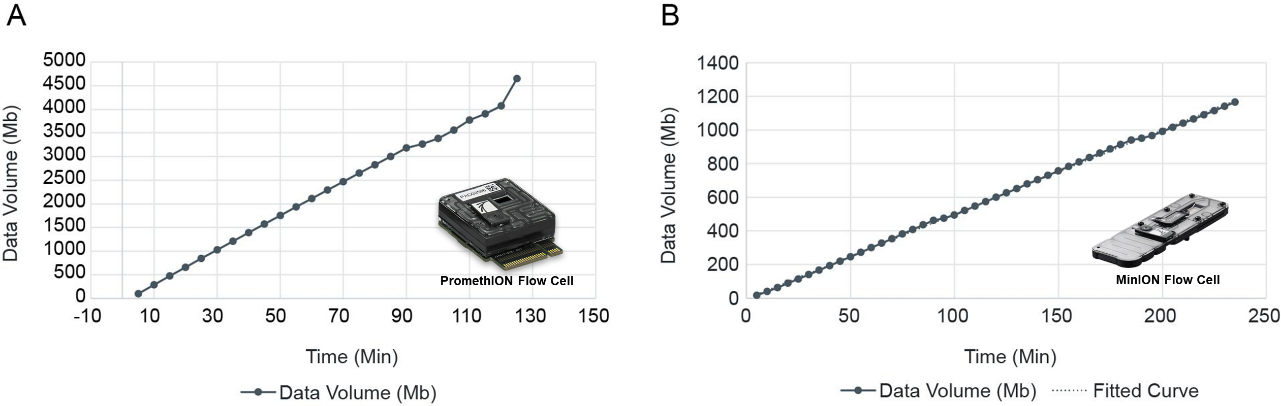


**Supplementary Figure S3 Phylogenetic trees (Neighbor joining) were built using CVA6 Genome, P1, P2 and P3 region, respectively.** Red dot: Nanopore sequences; Green dot: Illumina sequences; Black dot: prototype strains of CVA6; Black triangle: representative protype strains of *Enterovirus alphacoxsackie*.

**
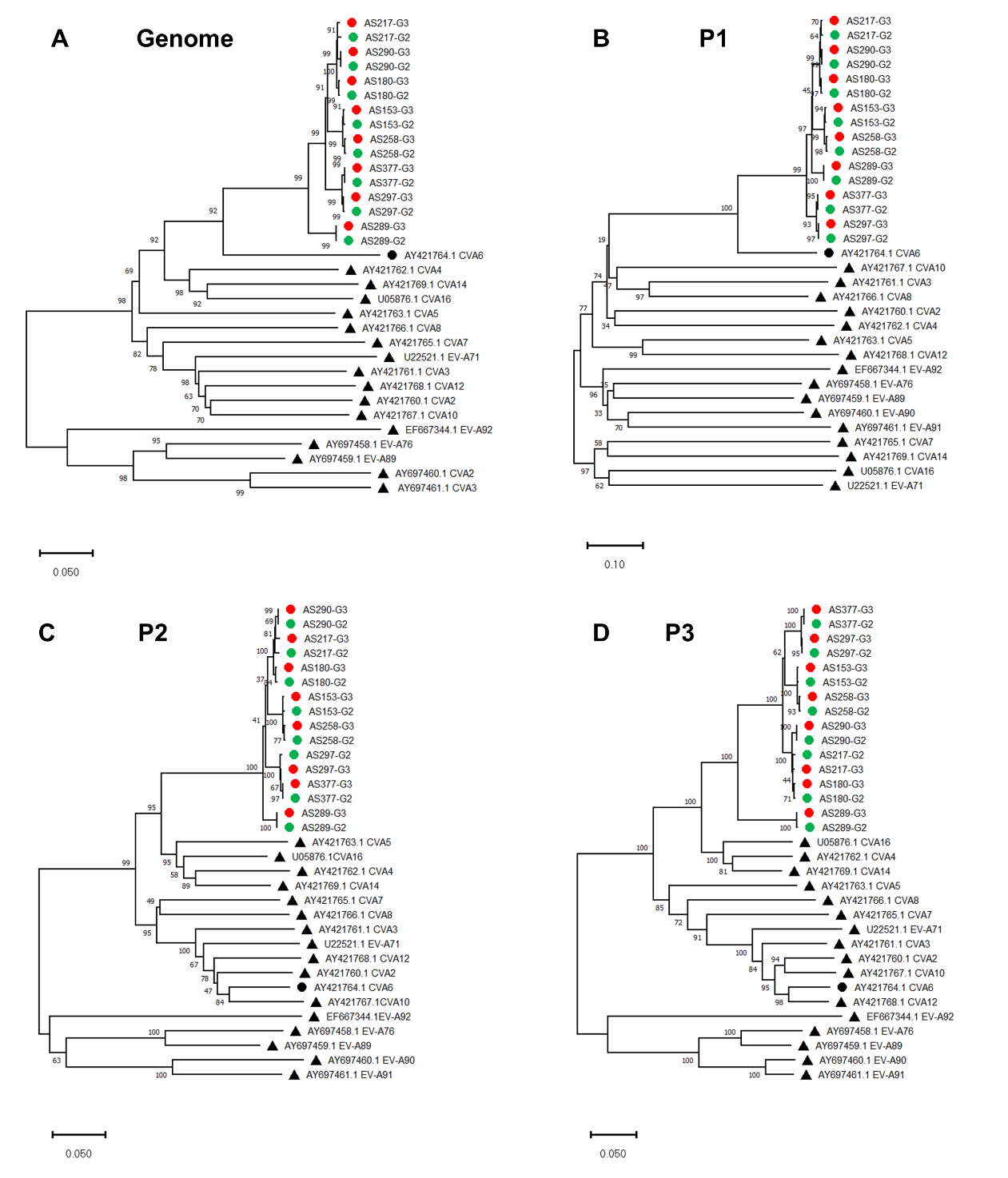
**

**Supplementary Table S1** Fifty CVA6 strains’ genome sequences downloaded from GenBank for primer design

| **Accession** | **Length** | **Completeness** | **Location** | **Host** | **Collection_Date** |
| --- | --- | --- | --- | --- | --- |
| MN845873 | 7435 | complete | China | Homo sapiens | 2017 |
| MN845874 | 7434 | complete | China | Homo sapiens | 2017 |
| MN845845 | 7435 | complete | China | Homo sapiens | 2018 |
| PV807107 | 7434 | complete | China | Homo sapiens | 2023 |
| MF285655 | 7434 | complete | China | Homo sapiens | 2015 |
| MN845824 | 7434 | complete | China | Homo sapiens | 2018 |
| PV807135 | 7434 | complete | China | Homo sapiens | 2023 |
| MN845852 | 7435 | complete | China | Homo sapiens | 2018 |
| MF285684 | 7434 | complete | China | Homo sapiens | 2016 |
| MF285660 | 7434 | complete | China | Homo sapiens | 2015 |
| OL830027 | 7434 | complete | China | Homo sapiens | 2019 |
| MN845816 | 7434 | complete | China | Homo sapiens | 2018 |
| MF285658 | 7434 | complete | China | Homo sapiens | 2015 |
| MK106204 | 7434 | complete | China | Homo sapiens | 2015 |
| PV807110 | 7434 | complete | China | Homo sapiens | 2023 |
| MN845828 | 7426 | complete | China | Homo sapiens | 2017 |
| MN845875 | 7434 | complete | China | Homo sapiens | 2017 |
| MF285673 | 7434 | complete | China | Homo sapiens | 2016 |
| MN845830 | 7434 | complete | China | Homo sapiens | 2017 |
| OL519577 | 7358 | complete | China | Homo sapiens | 2018 |
| PV807121 | 7434 | complete | China | Homo sapiens | 2023 |
| PV807115 | 7434 | complete | China | Homo sapiens | 2023 |
| MN845835 | 7435 | complete | China | Homo sapiens | 2017 |
| MN845864 | 7435 | complete | China | Homo sapiens | 2018 |
| MN845884 | 7434 | complete | China | Homo sapiens | 2017 |
| MN845831 | 7434 | complete | China | Homo sapiens | 2018 |
| MF285681 | 7434 | complete | China | Homo sapiens | 2016 |
| MN845796 | 7435 | complete | China | Homo sapiens | 2016 |
| MN845839 | 7434 | complete | China | Homo sapiens | 2017 |
| OR828445 | 7476 | complete | China | Homo sapiens | 2019 |
| MN845788 | 7434 | complete | China | Homo sapiens | 2016 |
| MN845850 | 7435 | complete | China | Homo sapiens | 2017 |
| OL830030 | 7434 | complete | China | Homo sapiens | 2019 |
| MH049747 | 7451 | complete | China | Homo sapiens | 2016 |
| MN845811 | 7436 | complete | China | Homo sapiens | 2018 |
| MN845799 | 7434 | complete | China | Homo sapiens | 2016 |
| PV807112 | 7434 | complete | China | Homo sapiens | 2023 |
| PV807111 | 7434 | complete | China | Homo sapiens | 2023 |
| MN845812 | 7435 | complete | China | Homo sapiens | 2017 |
| PV807131 | 7434 | complete | China | Homo sapiens | 2023 |
| OR828441 | 7456 | complete | China | Homo sapiens | 2019 |
| MN845838 | 7434 | complete | China | Homo sapiens | 2017 |
| MF285680 | 7435 | complete | China | Homo sapiens | 2016 |
| PV807133 | 7434 | complete | China | Homo sapiens | 2023 |
| PV807142 | 7434 | complete | China | Homo sapiens | 2023 |
| KX064297 | 7434 | complete | China | Homo sapiens | 2015 |
| MN845814 | 7435 | complete | China | Homo sapiens | 2017 |
| OL839937 | 7413 | complete | China | Homo sapiens | 2018 |
| MN845834 | 7436 | complete | China | Homo sapiens | 2017 |
| MN845792 | 7434 | complete | China | Homo sapiens | 2016 |

**Supplementary Table S2** Tiling primers for targeted sequence capture of coxsackievirus A6

| **Primers** | **Sequence (5’-3’)** | **Position(nt)** | **Pools** | **Ratios** |
| --- | --- | --- | --- | --- |
| CVA6_1_F1 | TTAAAACAGCCTGTGGGTTGYAC | 1-23 | Pool 1 | 1.0 |
| CVA6_1_F2 | CGCTAGCACACTGATTCTAYGGAA | 45-68 | Pool 1 | 1.0 |
| CVA6_1_R1 | TRCACTGAAYGTGCATGCAGAA | 1270-1291 | Pool 1 | 1.0 |
| CVA6_1_R2 | TACAYTGAACGTGCATGCARAA | 1270-1291 | Pool 1 | 1.0 |
| CVA6_2_F | GCTGTGGACAARCCYACTCG | 1099-1118 | Pool 2 | 1.5 |
| CVA6_2_R | TGTGCYGCTCCAAGAGCTAT | 2359-2378 | Pool 2 | 1.5 |
| CVA6_3_F | AAGCAGCAATGCTTGGGACT | 2156-2175 | Pool 1 | 1.0 |
| CVA6_3_R | CCTTCATAATCCGTRGTGGTTATGC | 3302-3326 | Pool 1 | 1.0 |
| CVA6_4_F | AGAATTAAGCACGTGAGARCTTGG | 3187-3210 | Pool 2 | 1.0 |
| CVA6_4_R | CAATACGGTGTTTGCTCTTGAACTG | 4441-4465 | Pool 2 | 1.0 |
| CVA6_5_F | TACCAGCAGCYAGAGAGAAGGT | 4212-4235 | Pool 1 | 1.0 |
| CVA6_5_R | CCCARCATAGTGAAGTGYCCTTG | 5479-5501 | Pool 1 | 1.0 |
| CVA6_6_F | GCTCCTAARCAAGTGCTCAAGA | 5365-5386 | Pool 2 | 1.0 |
| CVA6_6_R1 | GGTTTGCATGGAAAGYTTCATAGAG | 6544-6569 | Pool 2 | 1.0 |
| CVA6_6_R2 | GGTTTGCATGGAAGGYTTCATAGAG | 6544-6569 | Pool 2 | 1.0 |
| CVA6_7_F | TGAAGTTYTACATGGAYAAGTATGGYCT | 6386-6413 | Pool 1 | 1.0 |
| CVA6_7_R1 | CCACCAGTYATATTCMYGACCAGA | 7384-7407 | Pool 1 | 1.0 |
| CVA6_7_R2 | CCACCAGTCGTATTCAYKACCAGA | 7384-7407 | Pool 1 | 1.0 |
| CVA6_7_R3 | AATTCGAGCCAATTRCGTCTCAG | 7324-7346 | Pool 1 | 1.0 |

**Supplementary Table S3** PCR components per reaction

| **Reagent** | | **Pool1** | **Pool2** |
| --- | --- | --- | --- |
| cDNA | | 2.5 µL | 2.5 µL |
| 2×Q5 Hotstart Hifi Mix | | 12.5 µL | 12.5 µL |
| Primer | Primer Pool 1 | 4 µL | - |
|  | Primer Pool 2 | - | 4 µL |
| Nuclease-Free Water | | 6 µL | 6 µL |
| Total | | 25 µL | 25 µL |

**Supplementary Table S4** PCR program

| **PCR step** | **Temp** | **time** | **cycle** |
| --- | --- | --- | --- |
| 1 | 98℃ | 30 s | 1 |
| 2 | 98℃ | 15 s | 5 |
|  | 63℃ | 5 min |  |
| 3 | 98℃ | 15 s | 25 |
|  | 65℃ | 4 min |  |
| 4 | 72℃ | 2 min | 1 |
| 5 | 4℃ | Hold |  |

**Supplementary Table S5** Statistical summary of data filtering, sequence blast, and sample assembly from Illumina sequencing

| **SampleID** | **AS153** | **AS180** | **AS217** | **AS258** | **AS29** | **AS297** | **AS289** | **AS377** |
| --- | --- | --- | --- | --- | --- | --- | --- | --- |
| **Raw_reads** | 8196110 | 7966226 | 8006558 | 8008890 | 4664190 | 6035406 | 5515162 | 5408372 |
| **Clean_reads** | 8059050 | 7806246 | 7826804 | 7842406 | 4572634 | 5929214 | 5398938 | 5295876 |
| **Raw_bases** | 1.23E+09 | 1.19 E+09 | 1.2 E+09 | 1.2E+09 | 7E+08 | 9.05E+08 | 8.27 E+08 | 8.11E+08 |
| **Clean_bases** | 1.14E+09 | 1.1 E+09 | 1.1 E+09 | 1.11E+09 | 6.58E+08 | 8.5E+08 | 7.7 E+08 | 7.6E+08 |
| **GC (%)** | 46.26 | 46.62 | 47.07 | 46.56 | 46.43 | 46.28 | 46.09 | 46.01 |
| **Q20** | 97.23 | 97.38 | 97.35 | 97.27 | 97.06 | 97.13 | 97.13 | 97.25 |
| **Q30** | 94.15 | 94.43 | 94.37 | 94.22 | 93.78 | 93.91 | 93.95 | 94.16 |
| **Reference** | MK967650 | MK967650 | MK967650 | MK967650 | MK967650 | PV693693 | PV296183^#^ | PV693693 |
| **mapped_reads** | 7552290 | 7382148 | 7539822 | 7446800 | 4403144 | 5663546 | 4916794 | 5150155 |
| **mapping_rate** | 93.71 | 94.57 | 96.33 | 94.96 | 96.29 | 95.52 | 91.07 | 97.25 |
| **mean_depth** | 134829.4 | 132753.821 | 135591.818 | 133782.8 | 79261.14 | 102664.4 | 88628.601 | 92998.45 |
| **coverage** | 99.24 | 99.18 | 98.85 | 99.24 | 99.22 | 99.95 | 99.96 | 99.96 |
| **Length(bp)** | 7425 | 7425 | 7425 | 7425 | 7425 | 7425 | 7425 | 7425 |
| **GC (%)** | 46.96 | 47.04 | 47.18 | 46.99 | 47.04 | 47.07 | 47.58 | 47 |
| **N** | 0 | 0 | 0 | 0 | 0 | 0 | 0 | 0 |

^#^ De novo assembly and sanger sequencing were conducted
