# Supplementary material for: Targeted sequence capture of coxsackievirus A6 using nanopore sequencing directly from clinical specimens
Source: Microbiol Spectr. 2026 Jun 4;14(7):e03246-25. doi: 10.1128/spectrum.03246-25 (PMC13340022; doi:10.1128/spectrum.03246-25)
Supplement: Reviewer comments [file reviewer-comments.pdf]

AMERICAN SOCIETY FOR MICROBIOLOGY | Microbiology Spectrum

Targeted sequence capture of coxsackievirus A6 using Nanopore sequencing directly from clinical specimens

Ziqi Lin, Fenglan He, Han Mo, Lingfeng Mao, Xingyu Xu, Liu Yi, Ke Qian, Xiansheng Ni, Tielong Xu, Xianfeng Zhou, and Hui Li

Corresponding Author(s): Hui Li, Nanchang City Center for Disease Control and Prevention

Review Timeline:

Submission Date:	October 10, 2025
Editorial Decision:	December 19, 2025
Revision Received:	January 28, 2026
Editorial Decision:	February 15, 2026
Revision Received:	March 30, 2026
Editorial Decision:	April 26, 2026
Revision Received:	May 5, 2026
Accepted:	May 10, 2026

Editor: My Phan

Reviewer(s): Disclosure of reviewer identity is with reference to reviewer comments included in decision letter(s). The following individuals involved in review of your submission have agreed to reveal their identity: Temitope OC Faleye (Reviewer #1); Benjamin von Bredow (Reviewer #2)

Transaction Report:

(Note: With the exception of the correction of typographical or spelling errors that could be a source of ambiguity, letters and reports are not edited. The original formatting of letters and referee reports may not be reflected in this compilation.)

DOI: https://doi.org/10.1128/spectrum.03246-25

Re: Spectrum03246-25 (Targeted sequence capture of coxsackievirus A6 using Nanopore sequencing directly from clinical specimens)

Dear Dr. Xianfeng Zhou:

Thank you for submitting your work to Spectrum.

Your manuscript has been reviewed by three experts in the field, who raised several concerns, especially with some conclusions that are not supported by the data presented. The reviewers have provided substantial constructive feedback to improve the reading flow and the accuracy when describing the data. I encourage you to take into account these suggestions to modify your manuscript. We welcome your resubmission of the revised manuscript after addressing all the reviewers' comments and suggestions.

Below you will find my comments, instructions from the Spectrum editorial office, and the reviewer comments.

In addition to the reviewers' comments, please make sure that you specify the concentration of each sequencing primer used in the reaction and describe the bioinformatic tools that were used to design the primers. All the sequencing reads generated must be deposited to ENA or SRA and make publicly available and describe in the "Data Availability" section.

There were concerns that the English language usage in the manuscript might make it difficult to properly evaluate the science. The ASM Journals webpage provides links to various language editing services (https://journals.asm.org/writing-your-paper#language-editing-services). You may consider using these services when revising your manuscript. The use of these services will have no direct bearing on the editorial decision. ASM has no affiliation with these companies.

Please return the manuscript within 60 days; if you cannot complete the modification within this time period, please contact me. If you do not wish to modify the manuscript and prefer to submit it to another journal, notify me immediately so that the manuscript may be formally withdrawn from consideration by Spectrum.

Revision Guidelines
To submit your modified manuscript, log into the submission site at https://spectrum.msubmit.net/cgi-bin/main.plex. Go to Author Tasks and click the appropriate manuscript title to begin. The information you entered when you first submitted the paper will be displayed; update this as necessary. Note the following requirements:

• Upload point-by-point responses to the issues raised by the reviewers in a file named "Response to Reviewers," NOT in your cover letter.
• Upload a compare copy of the manuscript (without figures) as a "Marked-Up Manuscript" file.
• Upload a clean .DOC/.DOCX version of the revised manuscript and remove the previous version.
• Each figure must be uploaded as a separate, editable, high-resolution file (TIFF or EPS preferred), and any multipanel figures must be assembled into one file.
• Any supplemental material intended for posting by ASM should be uploaded with their legends separate from the main manuscript. You can combine all supplemental material into one file (preferred) or split it into a maximum of 10 files with all associated legends included.

For complete guidelines on revision requirements, see our Submission and Review Process webpage. Submission of a paper that does not conform to guidelines may delay acceptance of your manuscript.

Data availability: ASM policy requires that data be available to the public upon online posting of the article, so please verify all links to sequence records, if present, and make sure that each number retrieves the full record of the data. If a new accession number is not linked or a link is broken, provide Spectrum production staff with the correct URL for the record. If the accession numbers for new data are not publicly accessible before the expected online posting of the article, publication may be delayed; please contact production staff (Spectrum@asmusa.org) immediately with the expected release date.

Publication Fees: For information on publication fees and which article types are subject to charges, visit our website. If your manuscript is accepted for publication and any fees apply, you will be contacted separately about payment during the production process; please follow the instructions in that e-mail. Arrangements for payment must be made before your article is published.

ASM Membership: Corresponding authors may join or renew ASM membership to obtain discounts on publication fees. Need to upgrade your membership level? Please contact Customer Service at Service@asmusa.org.

The ASM Journals program strives for constant improvement in our submission and publication process. Please tell us how we can improve your experience by taking this quick Author Survey.

Thank you for submitting your paper to Spectrum.

Sincerely,
My Phan
Editor
Microbiology Spectrum

Reviewer #1 (Public repository details (Required)):

Raw reads should be submitted to the SRA alongside the remaining assembled genomes.

Reviewer #1 (Comments for the Author):

General Comments
Introduction
1. Line 52: There are 15 EV species not four. Please see the link below for more information https://www.picornaviridae.com/ensavirinae/enterovirus/enterovirus.htm
2. It will benefit the readers if the authors provide more information on enterovirus classification and genome organization in the introduction section. That way, readers will better understand what is meant by 2A-3C region in the results and discussion sections. Also clearly mention the EV species to which CVA6 belongs.
3. Line 54: Please clearly provide information on genetic criteria for typing EVs. This was not detailed anywhere in the manuscript. However, it is essential to confirm that the real time RT-PCR assay worked as expected.
4. Please read through the manuscript for slight grammar corrections especially with the use of tenses and articles
Methods
5. Overall, the methods section requires careful rework to ensure the sequential flow of the protocol is clear to all the readers. Furthermore, in its current form, there is some circularity in the way the method is presented in addition to several important steps that are not detailed.
6. Line 93: Please detail what type of samples were collected, what year they were collected and if any kind of processing was done prior to RNA extraction.
7. Lines 96-97: Please specify the kit used.
8. Lines 99-100: Please detail the transition from 8 samples to 24 here, so that it is very clear to the readers that the 24 samples are derived from the 8.
9. Line 100 says 24 samples were sequenced while line 285 says 10. Which is the correct one? Please carefully rework the methods section for consistency.
10. Lines 111 - 119: Multiple methods are detailed for different steps in the cDNA synthesis workflow. Please be precise. Which of them was used in this study. If both were used, then please detail which was used for what purpose.
11. Line 123: This suggests that one genome was used for primer design for a virus documented to undergo recombination very often. Line 351 said a 'limited set' was used. Therefore, please harmonize for accuracy.
12. Co-infection is very common with EVs and there is no effort documented in this study detailing how the authors confirmed the 8 samples did not have another EV present, especially another member of EV-A species. This is essential because recombination is very common in the nonstructural genomic region (NSGR) of EVs. If indeed, one genome isolated before 2004 was used for primer design, the possibility of co-infection was not ruled out, genomes were assembled using template guided assembly (Minimap), and there is variation in success of the assays especially assays 6 and 7 in the tile (Figure 3), without showing via de novo assembly that CVA6 is the only EV amplified in each of the samples, it might be difficult conclude that the assay described amplifies only CVA6.
13. Lines 132 and 150-151 suggest different master mixes were used for amplification for ONT and Illumina. If this is the case, please explain why the study was so designed. Also, kindly detail thermal cycler used, RT-PCR reaction conditions and volumes of master mix components per reaction and primer concentrations.
14. Lines 171-190: Please rephrase the bioinformatics section to ensure the sequential flow of the protocol is clear to all the readers. There is some circularity in the way the method is currently presented. Also, ensure the purpose of the different tools is accurately assigned. For example, multiple sequence alignment should not be the last thing. It should have happened before phylogenetic trees were inferred. Please ensure the sequential flow of the protocol is clear to all the readers. Also, since Guppy demultiplexes, why use Porechop (which is less accurate) for it. What tool was used for trimming, etc.

Results
15. Lines 202 - 203: This is part of library preparation. Why repeat it?
16. Line 215: Throughout the manuscript, please ensure real-time RT-PCR is clearly differentiated from just RT-PCR.
17. Figure 3: Please add schematic representation of genome architecture to panels A -I. This will help readers better understand what part of the genome the different contigs represent.
18. Line 236: Kindly consider changing "dada" to "data".
19. Lines 238-239: There is nothing highlighting 30% in figure 4A. Please confirm.
20. Line 242: The is no "500Mb" in figure 4B, Please confirm.
21. Lines 251-276: Please be clear and consistent. Is it GridION and PromethION or MinION and PromethION? Also, for a

balanced argument on output and cost kindly let the readers know the number of pores each equipment provides and initial cost of acquisition.
22. Lines 259 -260: How does one measure genomic integrity from Figure 5?
23. Figure 6: How did we go from 8 to 24 and back to 10 and why? Please be clear. Also what is the criteria for selecting the 50 sequences from GenBank.
24. Kindly add a table that shows BLAStn results of all the contigs alongside their pairwise identity with the top 2 hits in GenBank.
25. Sequences submitted to GenBank are PQ458981 - PQ458990. Why not submit the rest alongside the raw reads to SRA so that readers can independently verify the conclusions of the paper.
Discussion
26. Please cite the Tables and Figures in the discussion so that readers can easily make the connections and verify the claims.
27. Please ensure all the claims in the discussion are supported by the results.

Reviewer #2 (Comments for the Author):

Spectrum03246-25
In this article, the authors evaluate a novel assay for the rapid sequencing of coxsackievirus A6 direct from patient specimens using tiling PCR followed by nanopore sequencing. The authors demonstrate good coverage and strain resolution with this assay compared to Illumina sequencing. The authors further discuss potential quality metrics and comment on cost effectiveness of different flow cells.
This manuscript shows what appears to be a solid assay for detection and sequencing of coxsackievirus A6, but unclear language, confusing organization, and unsupported claims detract significantly from the core method described.
Major comments:
- Materials and methods section is poorly organized; important information is missing, some information is duplicated, and information that should be presented in the results section is included in the methods section. A non-exhaustive list of examples include redundant description of RNA extraction in sample collection and molecular identification section and cDNA synthesis section, description of overall design (lines 100-102) in the sample collection and molecular identification section, missing description of how samples were selected, missing explanation of how the different cDNA amplification primers were used or how gene-specific primers are defined, use of the term 'depleted' DNA with no discernible depletion step, the sudden introduction of '24' samples from the prior 8 samples (following dilution, presumably), a Figure 1 that is described in the text as showing the Nanopore library prep and sequencing workflow but really shows the overall workflow, sample types (5 throat 3 rectal) in the primer design section, etc.
- Paragraph 218-224 is difficult to follow and draws several conclusions that don't appear to be supported by data or if they are, the connections are not clear.
o The authors state that "coverage after dilution was 'relatively poor' (how is that defined?), which was affected by the concentration (Table 2)". However, all but two samples have coverage of at least 0.981 at the highest dilution; all but two undiluted samples have coverage of 0.981 with only two above this value. There are even two instances of the highest dilution samples having better coverage than their undiluted counterparts. Which concentration are the authors talking about? No concentrations are listed in Table 2. How is the second part of this sentence supported by the data?
o "The high Ct value at high concentration might be due to degradation" - which concentration are the authors referring to? Figure 2? It would be difficult for a reader to review Figure 2 and Table 2 side-by-side. The concentration data from Figure 2 could be integrated into Table 2, if this is indeed the case. Either way, looking at Figure 2, there seems to be no clear correlation between Ct value and library concentration (not a huge surprise, in my opinion), and certainly there is no association between high concentrations and high Ct values.
o "The increase in dilution concentration would not lead to sample degradation" - what does 'dilution concentration' mean? What data are the authors referring to when stating that an increase would not lead to sample degradation?
o "The capture efficiency reached up to 90%" - what does capture efficiency refer to? CVA6/Yield? What is the relevance of this number? A sample with only 24% CVA6/Yield has the highest observed genome coverage at 0.991
o "Based on the established CVA6 capture protocol and considering the actual Ct values of the samples, 150 Mb data will definitely enable the acquisition of the near-complete genome sequence of CVA6 from clinical samples" - this sentence makes a definitive conclusion, without providing any data to support that specific cut-off, and basing it on vague language that doesn't explain anything; Nothing in Table 2 correlates Ct values with Yield (is 150 Mb total yield or CVA6-specific yield? Not clear) or yield with coverage; some of this is presented later, but at this point, this is fully unsupported. It is not clear what 'based on the established CVA6 capture protocol' means or how it informs the conclusion drawn.
- "Data quality and amplification efficiency" section of Results
o "When the Ct value was greater than 28, the percentage of A6 data was below 30%" - One sample with Ct 29.11 had over 50% CVA6/Yield, one with a Ct of 27.39, had a percentage of 64.5%. How were the 30% (or Ct 28) thresholds chosen as significant? There is certainly a loose correlation between Ct and %CVA6, but the thresholds seem arbitrary.
o In Fig. 4A, it is not clear what is meant by Amplification Efficiency, or which data set goes with the linear regression. The label of "Data quality" in the figure legend is vague.
o "The genomic integrity performance of the genome in the amplified region of A6 reached 97% when the data volume of A6 was greater than 50 Mb (Figure 4)" - First of all Fig. 4B (panels aren't labeled) seems beset by rounding errors; visually, all but 2

samples reached 97%, yet many of those are actually at 96.5%. There only appears to be one sample at <96.5%, which is 95.5%, but multiple 96.5%. I can't figure out which samples were graphed at 96% and 95% - the single 95.5% can only account for one of those, depending on if you round up or down. The threshold of 50 Mb is once again arbitrary - the next two lower volume samples have some of the higher coverage percentages. Overall, the graph seems mostly flat with maybe a slight slope. The decision to use a line to connect the data points in a scatter plot is confusing.
o Thresholds for data volume by Ct value seem arbitrary and not supported by data. This section cites Fig. 4B, but Fig. 4B does not mention Ct values. There is no data to support a minimum data volume of 100-200 Mb for Ct 25-28, and certainly no data to support data volume of >500 Mb for Ct >28; none of the data presented has data volume >215 Mb.
- "Comparative analysis of amplification efficiency and cost using different flow cells" section
o Line 257 "evaluate the capture efficiency, minimum sequencing data volume" - not clear what this means. Does this refer to the minimum sequencing data volume needed, or the minimum data volume generated at each time point across the various samples?
o Was one sample evaluated? All 24? Only one graph shown for each flow cell, no error bars.
o Line 259f. how was 'genomic integrity' measured? No data shown in Fig 5, only data volume
o Authors state that lower cost per GB for PromethION cell makes it more cost effective. Is that scale actually necessary to sequence a reasonable daily volume of samples? Economies of scale only apply at scale.
- "Comparative analysis of CVA6 genomes obtained from Nanopore and Illumina sequencing"
o This section now has 10 samples instead of 8. These additional 2 samples are not described elsewhere. Why were they added/why are they missing from the rest of the analysis?
o The color coding in Fig. 6A could be clearer if the two data points (Illumina and Nanopore) for each sample were clearly identified by using the same color. Country/region is not necessarily required but could be retained by using different symbols. Similarly, Fig. 6B would be easier to read if the same sample had the same color and the different sequencers were identified by different symbols.
- Discussion
o L313ff. It is not clear to me that the PromethION has a significant advantage. MinION flow cells reached the 'recommended' data volume, even the 500 Mb, in under 2 hours. The authors suggest that this time difference is critical for informing public health response, but it is not clear in what scenario a difference of under 2 hours would be crucial in this context.
o The authors claim to have established clear, data driven guidelines for sequencing requirements based on viral load. Based on all of the points mentioned previously, I have to disagree. Viral loads were not established - a Ct value is not a viral load. There is no clear path from the raw data to the recommendations, and certainly no statistics to support these claims.
o Line 322, the authors describe % identity between sequences. Additionally, all 'same' samples from different platforms cluster more closely to each other than to different samples, further supporting that this assay provides sufficient accuracy for relatedness analyis, compared to Illumina.
o Line 328ff., the authors suggest that inconsistencies between Nanopore and Illumina may be due to amplicon drop-out or mapping ambiguities. While possible, the assay as a whole is what it is (and actually seems to perform well in this regard). This is more a limitation than something that can be explained away - and doesn't need to, since the overall performance appears to be just fine.
o As mentioned above, cost and speed comparison of MinION and PromethION seem mostly academic and not relevant for real-world application of this assay (L335f.)
o L 356ff. "In conclusion, 356 the integration of the wet-lab protocol with automated, user-friendly bioinformatic pipelines for real-time base calling, variant analysis, and phylogenetic tree construction would significantly augment its utility in frontline public health laboratories." This is not a conclusion and does not follow from any of the preceding points.
- Abstract methods section refers to 24 samples - it was 8 (or maybe 10?) samples, plus some dilutions.
- Line 97, Ct values are not quantitative without the appropriate standards and controls. Semi-quantitative at best.
- Figure 1 mentions only throat swabs; graphical abstract mentions throat swabs and feces (using a cup image), method section (under tiling primers design and amplicon) mentions throat and anal (rectal?) swabs
- Does the bioinformatics section apply to Illumina data or is Illumina analysis not described? If so, add at least a reference and a brief summary.
- Library construction under the Results section describes a method; results shown are pretty basic (DNA concentrations and gel images) - that's fine but doesn't add much and is not discussed anywhere.

Minor comments
- Line 58, describe 'atypical' HFMD
- Line 97, cite if using 'as previously reported'
- Line 99f., what does gradient dilution mean?
- Line 116, abbreviation "GSP" not previously defined
- Lines 106-119, the entire section is written in active voice
- Line 148, cDNA synthesis method has been defined earlier (twice)
- Line 157f., 'measured with a Qubit' is colloquial and imprecise
- Line 159f., description of the Bioanalyzer as 'a sophisticated instrument that provides comprehensive analysis of DNA libraries' does not tell the reader what it does exactly or how it was used.
- Line 202, what does 'linking the barcodes to the libraries' mean?
- Fig 3A is not clearly distinct from Fig 3B-I; consider moving this to a separate figure
- Which sample is shown in Fig 3A? Is there a more legible way to represent coverage over time, maybe a average+range graph

over time? Especially at the top end it becomes difficult to distinguish the curves by color.
- Fig 3C: Should this be AS180? AS189 is not mentioned anywhere else.
- Fig 3B-I: At which time point are these measured?
- Some typos (L124 annal swab, L236 dada, L221 Th)
- Table 2, showing the barcode number here is not necessary

Reviewer #3 (Comments for the Author):

Major comments:
1) L250-276: I don't see the value of comparing the same sequencing technology. In addition, the conclusions from this section, ie. PromethION has more output than MinION flow cells and is more cost-effective for bigger data volumes, are disclaimed by Oxford Nanopore technologies. This differences are there by design. In addition the cost-effectiveness conclusion does not take into account that the MinION flowcell is cheaper by unit, which is still the best choice for lower throughput needs. I also don't see any data on genome integrity in Figure 5. I would suggest to remove this whole section or pivot and compare nanopore sequencing to the Illumina sequencing.
2) I'm missing some discussion on why the region between 5000-6000 nucleotides consistently has a low coverage (Figure3), what could the reasons be and how could they be solved? Is it a region where the primers do not easily bind (eg. secondary structures) or is this because this region is more divergent from the strain the primers were designed on, etc.? Certainly because this does have an impact on the genome assembly as shown in Figure 6 with the most differences between Nanopore and Illumina in this region.
Minor comments:
L69-70: PromethION is not a portable sequencer, so it does not represent a significant advancement in portable sequencing technology. I think you mean nanopore technology in general or MinION.
L82: nt identical -> nucleotide identity
L83: sequencing -> sequencing protocol
L107-108: the first sentence seems redundant + the methods section should not be a protocol unless it is a protocol paper, I suggest to write out a detailed protocol in the supplementary data and keep the method description simple here and remove too detailed descriptions (eg. "Chill on ice. Store the cDNA synthesis reaction at -20{degree sign}C, or proceed directly to PCR.")
L170-190: Most of the used bioinformatic tools also have published papers, provided these as references instead of links where possible.
L202-203: I would suggest to remove this sentence as it belongs more in the method section
L210: 10-time -> 10-fold, 10-time might be interpreted as 10 dilutions
L218-219: the authors claim a poor coverage for high Ct samples but in the table they reference there is not much difference in the coverage between the dilutions. In additions, what does this coverage mean, depth/horizontal coverage? Please clarify and add a unit to the coverage column.
L219-221: I'm not sure what the authors try to show with the sentences about sample degradation. + where should I see that the capture efficiency in Table2?
L242-243: The authors claim that a data volume of 500Mb is enough to ensure genome-wide-coverage for samples with a Ct > 28, but the 500Mb is never shown in their data analyses?
L285: In the rest of the manuscript only 8 samples are mentioned?
L295: Is there something special about sample AS377 that it performs better than the other samples?
Figure 3A: Is this across a particular sample or are all samples pooled? Please clarify.
Figure 4: What do the blue and red dotted lines mean in this plot? What do the point shapes mean in panel A?
Figure 6: what does the scale in panel A represent?

Spectrum03246-25

In this article, the authors evaluate a novel assay for the rapid sequencing of coxsackievirus A6 direct from patient specimens using tiling PCR followed by nanopore sequencing. The authors demonstrate good coverage and strain resolution with this assay compared to Illumina sequencing. The authors further discuss potential quality metrics and comment on cost effectiveness of different flow cells.

This manuscript shows what appears to be a solid assay for detection and sequencing of coxsackievirus A6, but unclear language, confusing organization, and unsupported claims detract significantly from the core method described.

Major comments:

- Materials and methods section is poorly organized; important information is missing, some information is duplicated, and information that should be presented in the results section is included in the methods section. A non-exhaustive list of examples include redundant description of RNA extraction in sample collection and molecular identification section and cDNA synthesis section, description of overall design (lines 100-102) in the sample collection and molecular identification section, missing description of how samples were selected, missing explanation of how the different cDNA amplification primers were used or how gene-specific primers are defined, use of the term 'depleted' DNA with no discernible depletion step, the sudden introduction of '24' samples from the prior 8 samples (following dilution, presumably), a Figure 1 that is described in the text as showing the Nanopore library prep and sequencing workflow but really shows the overall workflow, sample types (5 throat 3 rectal) in the primer design section, etc.
- Paragraph 218-224 is difficult to follow and draws several conclusions that don't appear to be supported by data or if they are, the connections are not clear.
    - The authors state that "coverage after dilution was 'relatively poor' (how is that defined?), which was affected by the concentration (Table 2)". However, all but two samples have coverage of at least 0.981 at the highest dilution; all but two undiluted samples have coverage of 0.981 with only two above this value. There are even two instances of the highest dilution samples having better coverage than their undiluted counterparts. Which concentration are the authors talking about? No concentrations are listed in Table 2. How is the second part of this sentence supported by the data?
    - "The high Ct value at high concentration might be due to degradation" – which concentration are the authors referring to? Figure 2? It would be

difficult for a reader to review Figure 2 and Table 2 side-by-side. The concentration data from Figure 2 could be integrated into Table 2, if this is indeed the case. Either way, looking at Figure 2, there seems to be no clear correlation between Ct value and library concentration (not a huge surprise, in my opinion), and certainly there is no association between high concentrations and high Ct values.

  - "The increase in dilution concentration would not lead to sample degradation" – what does 'dilution concentration' mean? What data are the authors referring to when stating that an increase would not lead to sample degradation?
  - "The capture efficiency reached up to 90%" – what does capture efficiency refer to? CVA6/Yield? What is the relevance of this number? A sample with only 24% CVA6/Yield has the highest observed genome coverage at 0.991
  - "Based on the established CVA6 capture protocol and considering the actual Ct values of the samples, 150 Mb data will definitely enable the acquisition of the near-complete genome sequence of CVA6 from clinical samples" – this sentence makes a definitive conclusion, without providing any data to support that specific cut-off, and basing it on vague language that doesn't explain anything; Nothing in Table 2 correlates Ct values with Yield (is 150 Mb total yield or CVA6-specific yield? Not clear) or yield with coverage; some of this is presented later, but at this point, this is fully unsupported. It is not clear what 'based on the established CVA6 capture protocol' means or how it informs the conclusion drawn.

- "Data quality and amplification efficiency" section of Results
  - "When the Ct value was greater than 28, the percentage of A6 data was below 30%" – One sample with Ct 29.11 had over 50% CVA6/Yield, one with a Ct of 27.39, had a percentage of 64.5%. How were the 30% (or Ct 28) thresholds chosen as significant? There is certainly a loose correlation between Ct and %CVA6, but the thresholds seem arbitrary.
  - In Fig. 4A, it is not clear what is meant by Amplification Efficiency, or which data set goes with the linear regression. The label of "Data quality" in the figure legend is vague.
  - "The genomic integrity performance of the genome in the amplified region of A6 reached 97% when the data volume of A6 was greater than 50 Mb (Figure 4)" – First of all Fig. 4B (panels aren't labeled) seems beset by rounding errors; visually, all but 2 samples reached 97%, yet many of those are actually at 96.5%. There only appears to be one sample at <96.5%, which is 95.5%, but multiple 96.5%. I can't figure out which samples were graphed at 96% and 95% - the single 95.5% can only

account for one of those, depending on if you round up or down. The threshold of 50 Mb is once again arbitrary – the next two lower volume samples have some of the higher coverage percentages. Overall, the graph seems mostly flat with maybe a slight slope. The decision to use a line to connect the data points in a scatter plot is confusing.
    - Thresholds for data volume by Ct value seem arbitrary and not supported by data. This section cites Fig. 4B, but Fig. 4B does not mention Ct values. There is no data to support a minimum data volume of 100-200 Mb for Ct 25-28, and certainly no data to support data volume of >500 Mb for Ct >28; none of the data presented has data volume >215 Mb.
- "Comparative analysis of amplification efficiency and cost using different flow cells" section
    - Line 257 "evaluate the capture efficiency, minimum sequencing data volume" – not clear what this means. Does this refer to the minimum sequencing data volume needed, or the minimum data volume generated at each time point across the various samples?
    - Was one sample evaluated? All 24? Only one graph shown for each flow cell, no error bars.
    - Line 259f. how was 'genomic integrity' measured? No data shown in Fig 5, only data volume
    - Authors state that lower cost per GB for PromethION cell makes it more cost effective. Is that scale actually necessary to sequence a reasonable daily volume of samples? Economies of scale only apply at scale.
- "Comparative analysis of CVA6 genomes obtained from Nanopore and Illumina sequencing"
    - This section now has 10 samples instead of 8. These additional 2 samples are not described elsewhere. Why were they added/why are they missing from the rest of the analysis?
    - The color coding in Fig. 6A could be clearer if the two data points (Illumina and Nanopore) for each sample were clearly identified by using the same color. Country/region is not necessarily required but could be retained by using different symbols. Similarly, Fig. 6B would be easier to read if the same sample had the same color and the different sequencers were identified by different symbols.
- Discussion
    - L313ff. It is not clear to me that the PromethION has a significant advantage. MinION flow cells reached the 'recommended' data volume, even the 500 Mb, in under 2 hours. The authors suggest that this time difference is critical for informing public health response, but it is not clear

in what scenario a difference of under 2 hours would be crucial in this context.
    - The authors claim to have established clear, data driven guidelines for sequencing requirements based on viral load. Based on all of the points mentioned previously, I have to disagree. Viral loads were not established – a Ct value is not a viral load. There is no clear path from the raw data to the recommendations, and certainly no statistics to support these claims.
    - Line 322, the authors describe % identity between sequences. Additionally, all 'same' samples from different platforms cluster more closely to each other than to different samples, further supporting that this assay provides sufficient accuracy for relatedness analyis, compared to Illumina.
    - Line 328ff., the authors suggest that inconsistencies between Nanopore and Illumina may be due to amplicon drop-out or mapping ambiguities. While possible, the assay as a whole is what it is (and actually seems to perform well in this regard). This is more a limitation than something that can be explained away – and doesn't need to, since the overall performance appears to be just fine.
    - As mentioned above, cost and speed comparison of MinION and PromethION seem mostly academic and not relevant for real-world application of this assay (L335f.)
    - L 356ff. "In conclusion, 356 the integration of the wet-lab protocol with automated, user-friendly bioinformatic pipelines for real-time base calling, variant analysis, and phylogenetic tree construction would significantly augment its utility in frontline public health laboratories." This is not a conclusion and does not follow from any of the preceding points.
- Abstract methods section refers to 24 samples – it was 8 (or maybe 10?) samples, plus some dilutions.
- Line 97, Ct values are not quantitative without the appropriate standards and controls. Semi-quantitative at best.
- Figure 1 mentions only throat swabs; graphical abstract mentions throat swabs and feces (using a cup image), method section (under tiling primers design and amplicon) mentions throat and anal (rectal?) swabs
- Does the bioinformatics section apply to Illumina data or is Illumina analysis not described? If so, add at least a reference and a brief summary.
- Library construction under the Results section describes a method; results shown are pretty basic (DNA concentrations and gel images) – that's fine but doesn't add much and is not discussed anywhere.

Minor comments

- Line 58, describe ‘atypical’ HFMD
- Line 97, cite if using ‘as previously reported’
- Line 99f., what does gradient dilution mean?
- Line 116, abbreviation “GSP” not previously defined
- Lines 106-119, the entire section is written in active voice
- Line 148, cDNA synthesis method has been defined earlier (twice)
- Line 157f., ‘measured with a Qubit’ is colloquial and imprecise
- Line 159f., description of the Bioanalyzer as ‘a sophisticated instrument that provides comprehensive analysis of DNA libraries’ does not tell the reader what it does exactly or how it was used.
- Line 202, what does ‘linking the barcodes to the libraries’ mean?
- Fig 3A is not clearly distinct from Fig 3B-I; consider moving this to a separate figure
- Which sample is shown in Fig 3A? Is there a more legible way to represent coverage over time, maybe a average+range graph over time? Especially at the top end it becomes difficult to distinguish the curves by color.
- Fig 3C: Should this be AS180? AS189 is not mentioned anywhere else.
- Fig 3B-I: At which time point are these measured?
- Some typos (L124 annal swab, L236 dada, L221 Th)
- Table 2, showing the barcode number here is not necessary

Dear Dr. Xianfeng Zhou:

Thank you for submitting your work to Spectrum.

Your manuscript has been reviewed by three experts in the field, who raised several concerns, especially with some conclusions that are not supported by the data presented. The reviewers have provided substantial constructive feedback to improve the reading flow and the accuracy when describing the data. I encourage you to take into account these suggestions to modify your manuscript. We welcome your resubmission of the revised manuscript after addressing all the reviewers' comments and suggestions.

Response: Dear Dr. Phan, editor of *Microbiology Spectrum*, thank you so much for being in charge of the peer review, which definitely provides us valuable and constructive comments for further revision and high-quality improvement. After carefully abstracting each piece of opinion, we have addressed all of them point by point to the fullest extent possible. In this revised version, we thoroughly checked the gramma, tense, expression to improve the quality and readability with the support of English native speakers. We sincerely appreciate your effort and time in the article assessment, and hope you will give favorable consideration to this revised version and find the manuscript acceptable for publication in *Microbiology Spectrum*

Below you will find my comments, instructions from the Spectrum editorial office, and the reviewer comments.

In addition to the reviewers' comments, please make sure that you specify the concentration of each sequencing primer used in the reaction and describe the bioinformatic tools that were used to design the primers. All the sequencing reads generated must be deposited to ENA or SRA and make publicly available and describe in the "Data Availability" section.

There were concerns that the English language usage in the manuscript might make it difficult to properly evaluate the science. The ASM Journals webpage provides links to various language editing services (https://journals.asm.org/writing-your-paper#language-editing-services). You may consider using these services when revising your manuscript. The use of these services will have no direct bearing on the editorial decision. ASM has no affiliation with these companies.

Please return the manuscript within 60 days; if you cannot complete the modification within this time period, please contact me. If you do not wish to modify the manuscript and prefer to submit it to another journal, notify me immediately so that the manuscript may be formally withdrawn from consideration by Spectrum.

Response: we thank the editor for the assessment and specific comments on our work. We have submitted the sequencing reads to SRA and described the project in the "Data Availability" section (line 443-447). Based on the editor's concern on the English language usage, we have invited a native English speaker in our institute for proofreading of the manuscript, and we appreciated it in the "Acknowledgement". (line 454-456)

Reviewer #1 (Public repository details (Required)):
Raw reads should be submitted to the SRA alongside the remaining assembled genomes.

Response: The raw sequencing reads generated in this study have been deposited in the NCBI Sequence Read Archive (SRA) under BioProject accession number PRJNA1398593, with individual sample accessions ranging from SRX31713286 to SRX31713309. The assembled consensus genome sequences have been submitted to GenBank under accession numbers PQ458981 to PQ458990. This was stated in the "Data Availability" section. (line 443-447)

Comments for the Author:

General Comments

Introduction

1. Line 52: There are 15 EV species not four. Please see the link below for more information https://www.picornaviridae.com/ensavirinae/enterovirus/enterovirus.htm

Response: We appreciate that the reviewer's guide in updating the EV species. In the original manuscript, the four species (*Enterovirus A-D*) were not specified. We have updated the 15 EV species according to the reviewer's suggestion in with supportive citation as follows:

"The genus *Enterovirus* consists of 15 species: *Enterovirus alphacoxsackie, Enterovirus betacoxsackie, Enterovirus coxsackiepol, Enterovirus deconjuncti, Enterovirus eibovi, Enterovirus fitauri, Enterovirus geswini, Enterovirus hesimi, Enterovirus idromi, Enterovirus jesimi, Enterovirus krodeni, Enterovirus lesimi, Enterovirus alpharhino, Enterovirus betarhino and Enterovirus cerhino*. These were formerly named Enterovirus A-L, Rhinovirus A-C, respecively(9). The enterovirus genome is approximately 7,500 nucleotides (nt) in length and consists of a positive, single-stranded RNA structure. It contains two untranslated regions (5′ and 3′ -UTR) flanking a large open reading frame that encodes a polyprotein, which is subsequently cleaved into structural (1A-1D: VP4, VP2, VP3, VP1) and non-structural proteins (2A-2C,3A-3D) (Figure 1)(4).The complete VP1 sequence effectively distinguishes human enterovirus serotypes, with intraserotypic divergence generally below 25% nucleotide difference, making it a valuable molecular basis for typing and taxonomy. HFMD were mainly associated with serotypes from Enterovirus A (EV-A) and Enterovirus B (EV-B) that contain 25 and 63 serotypes, respectively(6)." (line 51-65)

2. It will benefit the readers if the authors provide more information on enterovirus classification and genome organization in the introduction section. That way, readers will better understand what is meant by 2A-3C region in the results and discussion sections. Also clearly mention the EV species to which CVA6 belongs.

Response: Thank the reviewer for this constructive suggestion. More information on EV classification was provided and a diagram of genome organization was included as Figure 1. And the EV species to which CVA6 belongs was specified according to the reviewer's suggestion (line72-73).

3. Line 54: Please clearly provide information on genetic criteria for typing EVs. This was not detailed anywhere in the manuscript. However, it is essential to confirm that the real time RT-PCR assay worked as expected.

Response: Complete VP1 sequence comparison was used for typing EVs as the complete VP1 sequence effectively distinguishes human enterovirus serotypes, with intraserotypic divergence generally below 25% nucleotide difference, making it a valuable molecular basis for typing and taxonomy (Oberste et al., 1999). In this study, typing was strictly based on the VP1 sequence with registered commercial kits that was detailed in methods (line 122-126).

4. Please read through the manuscript for slight grammar corrections especially with the use of tenses and articles

Response: We thank the reviewer for pointing out the grammar mistakes and the inappropriate use of tenses, the whole text was thoroughly checked, corrected and polished by an English native speaker (Acknowledgement).

Methods

5. Overall, the methods section requires careful rework to ensure the sequential flow of the protocol is clear to all the readers. Furthermore, in its current form, there is some circularity in the way the method is presented in addition to several important steps that are not detailed.

Response: we appreciate the reviewer's valuable comments on the methods section. In the revised version, we have substantially recomposed this section and set a protocol (steps) as supplementary data. Methods of real time RT-PCR, Primer design, library preparation, and bioinformatics were specified and detailed. (line 122-126, 146-155, 156-174, and 194-215)

6. Line 93: Please detail what type of samples were collected, what year they were collected and if any kind of processing was done prior to RNA extraction.

Response: We appreciate the reviewer's comment. Accordingly, we specified the first part of the methods, in which types of samples, processing was further detailed in line 117-119.

7. Lines 96-97: Please specify the kit used.

Response: we have provided the information for the kits used in this study in line 122-126.

8. Lines 99-100: Please detail the transition from 8 samples to 24 here, so that it is

very clear to the readers that the 24 samples are derived from the 8.

Response: we are grateful that the reviewer pointed out this issue. We have modified this part to detail the origin of the 24 samples analyzed in this study. And a new Figure 2 were provided to detail the transition from 8 samples diluted into 24.

9. Line 100 says 24 samples were sequenced while line 285 says 10. Which is the correct one? Please carefully rework the methods section for consistency.

Response: We thank the reviewer for pointing out the inconsistency of sample numbers and sorry for the confusing information. After establishing the nanopore sequencing for CVA6 using 8 representative clinical samples, additional samples were subjected to both nanopore sequencing and Illumina sequencing. We have carefully clarified the samples in the methods, results and conclusion for consistency in the revised manuscript. (Figure 2, Figure 6, Figure 7)

10. Lines 111 - 119: Multiple methods are detailed for different steps in the cDNA synthesis workflow. Please be precise. Which of them was used in this study. If both were used, then please detail which was used for what purpose.

Response: Again, sorry for the confusing information. Actually, cDNA synthesis workflow is consistent for the whole study. In the revised manuscript, cDNA synthesis was only described and detailed in the Methods to be precise. (line 133-144)

11. Line 123: This suggests that one genome was used for primer design for a virus documented to undergo recombination very often. Line 351 said a 'limited set' was used. Therefore, please harmonize for accuracy.

Response: we appreciate the reviewer's rigorous comments on the primer design and concern of the recombination. After reviewing the record, we conducted the primer design as follows. The "limited set" we used in the previous version was inaccurate, which was revised according the methods.

"Fifty complete CVA6 genomes from strains circulating in China between 2015 and 2023 were retrieved from the NCBI database, with genome lengths ranging from 7,358 to 7,476 nucleotides. The sequence alignment was performed using MAFFT v7.400, followed by initial primer design with PrimalScheme v3.2.3 (https://primalscheme.com/). Subsequently, each primer was manually reviewed and optimized to achieve >90% coverage. The primer pool ratios were optimized following sequencing to achieve balanced amplification, aiming for a maximum 5-fold difference in coverage depth across amplicons. The tiling primers were distributed into two pools for targeted sequence capture of CVA6, and the finalized primer ratios are presented in Supplementary Table S1." (line 145-155)

12. Co-infection is very common with EVs and there is no effort documented in this study detailing how the authors confirmed the 8 samples did not have another EV present, especially another member of EV-A species. This is essential because recombination is very common in the nonstructural genomic region (NSGR) of EVs. If indeed, one genome isolated before 2004 was used for primer design, the possibility of co-infection was not ruled out, genomes were assembled using template guided assembly (Minimap), and there is variation in success of the assays especially assays 6 and 7 in the tile (Figure 3), without showing via de novo assembly that CVA6 is the only EV amplified in each of the samples, it might be difficult conclude that the assay described amplifies only CVA6.

Response: we agree with the reviewer on co-infection of EVs. In the study design, the selected samples were identified by specific real time RT-PCR to exclude the co-infection of circulating serotypes (line 122-126). Moreover, the de-novo assembly was performed by flye (v2.9.6) (41) and raven (v1.8) (42), and then the contigs were blasted against with NCBI database (core_nt) to identified other members of *Enterovirus A* (line 203-205). The de-nova assembly data (Supplementary de-nova data) indicated no other EVs from the sequencing.

13. Lines 132 and 150-151 suggest different master mixes were used for amplification for ONT and Illumina. If this is the case, please explain why the study was so designed. Also, kindly detail thermal cycler used, RT-PCR reaction conditions and volumes of master mix components per reaction and primer concentrations.

Response: We thank the reviewer for this professional and valuable comment. In our laboratory, there are two sets of commercial kits for ONT and Illumina sequencing workflow. In the revised manuscript, we have added the detail of equipment in the methods and specific PCR condition in Supplementary Table S1-S3.

14. Lines 171-190: Please rephrase the bioinformatics section to ensure the sequential flow of the protocol is clear to all the readers. There is some circularity in the way the method is currently presented. Also, ensure the purpose of the different tools is accurately assigned. For example, multiple sequence alignment should not be the last thing. It should have happened before phylogenetic trees were inferred. Please ensure the sequential flow of the protocol is clear to all the readers. Also, since Guppy demultiplexes, why use Porechop (which is less accurate) for it. What tool was used for trimming, etc.

Response: It has been revised in the Bioinformatic analysis section of the text (line 194-215). Data splitting was performed using Guppy. Low-quality reads were filtered with fastp, and adapter sequences were trimmed using fastp. Quality control was assessed with FastQC. During actual data processing, multiple sequence alignment was conducted prior to phylogenetic tree construction. The sequence described in the text was reversed and has now been corrected.

Regarding the use of Porechop: adapter and barcode identification and splitting were performed using Guppy, as stated in the article. Porechop was primarily employed to remove chimeric sequences. The method includes a parameter, discard_middle, specifically designed for chimeric sequence removal, which has proven to be effective.

Results

15. Lines 202 - 203: This is part of library preparation. Why repeat it?

Response: We appreciate this important comment to make the manuscript flow better. Accordingly, this part was revised as “Sample preparation and serial dilution” section, and the library preparation description was combined into methods in line 227-233.

16. Line 215: Throughout the manuscript, please ensure real-time RT-PCR is clearly differentiated from just RT-PCR.

Response: This issue has been solved by thoroughly checking the description.

17. Figure 3: Please add schematic representation of genome architecture to panels A -I. This will help readers better understand what part of the genome the different contigs represent.

Response: thank you so much for this valuable suggestion. In the introduction part, we have added a schematic diagram of genome architecture, which will help readers better understand the specific part of the genome the different contigs represent. By the way, we polished the Figure to make it clearer (Figure 4)

18. Line 236: Kindly consider changing "dada" to "data".

Response: typo corrected in line 280

19. Lines 238-239: There is nothing highlighting 30% in figure 4A. Please confirm.

Response: 30% that mentioned in the text was marked in Figure 5A of the revised manuscript.

20. Line 242: The is no "500Mb" in figure 4B, Please confirm.

Response: It should be 50Mb, we have corrected this mistake in line 293.

21. Lines 251-276: Please be clear and consistent. Is it GridION and PromethION or MinION and PromethION? Also, for a balanced argument on output and cost kindly let the readers know the number of pores each equipment provides and initial cost of acquisition.

Response: Thank you for this important clarification. We apologize for the inconsistent terminology.

Correction: In our study, the sequencing platforms used for comparison were the MinION flow cell (run on a GridION sequencing device) and the PromethION flow cell (run on a PromethION 2 Solo device). To avoid confusion, we have revised the manuscript to consistently refer to them as MinION flow cells and PromethION flow cells.

Regarding throughput and cost, we have revised the discussion to provide a balanced perspective (line 412-419). The text now explains that while the PromethION flow cell (~3000 pores) offers higher total output due to its greater number of pores—a relevant factor for laboratories aiming to maximize sample multiplexing per run—the MinION flow cell (~500 pores) is capable of generating complete CVA6 genomes and may be the more practical choice for laboratories with lower throughput needs or existing MinION/GridION infrastructure. We have removed the direct cost-per-GB comparison to keep the focus on the technical suitability of the assay.

22. Lines 259 -260: How does one measure genomic integrity from Figure 5?

Response: This statement was not precise. We have corrected the whole paragraph and removed "integrity", which should be output. (line 280-298)

23. Figure 6: How did we go from 8 to 24 and back to 10 and why? Please be clear. Also what is the criteria for selecting the 50 sequences from GenBank.

Response: We thank the reviewer for pointing out the inconsistency of sample numbers and sorry for the confusing information. After establishing the nanopore sequencing for CVA6 using 8 representative clinical samples, additional samples were subjected to both nanopore sequencing and Illumina sequencing. We have carefully clarified the samples in the methods, results and conclusion for consistency in the revised manuscript. And a new Figure 2 were provided to detail the transition from 8 samples diluted into 24.

The 50 reference sequences included in the phylogenetic analysis were selected from GenBank based on two primary criteria to ensure a comprehensive and informative evolutionary context:

a. Spatiotemporal Distribution: We aimed to include strains collected from diverse geographic regions and across multiple years. This allows the tree to reflect both the global circulation and the temporal spread of CVA6.

b. Genotypic Representation: Sequences were chosen to represent the major known genotypes and sub-genotypes of CVA6 (particularly subgenotype D3 and its

subgroups), ensuring that the phylogenetic placement of our newly sequenced strains could be accurately assessed within the existing global diversity framework.

This curated set provides a robust background against which to evaluate the origins and relationships of the strains sequenced in our study. However, we mainly aim to evaluate the pairwise sequence identities within samples.

24. Kindly add a table that shows BLAStn results of all the contigs alongside their pairwise identity with the top 2 hits in GenBank.

Response: Thank you for the suggestion. We have included a pairwise identity heatmap (Figure 6A, 6B) that visually represents the sequence similarity between the consensus genomes generated in this study and the top matching reference strains from GenBank. This format effectively conveys the high degree of identity (99.37%-99.98%) and the clustering patterns among the studied strains and their closest database matches, which aligns with the core objective of assessing sequence accuracy and phylogenetic placement. We believe the heatmap provides a clear and efficient overview of these relationships. (line 348-350)

25. Sequences submitted to GenBank are PQ458981 - PQ458990. Why not submit the rest alongside the raw reads to SRA so that readers can independently verify the conclusions of the paper.

Response: we thank the reviewer's suggestion. Accordingly, the raw reads were submitted to SAR and the specific information of the project was added into the data availability as follows:

Data Availability

The genome sequences of CVA6 strains in this study were submitted to GenBank with accession no. PQ458981-PQ458990; All the nanopore sequencing reads generated were deposited to SRA with BioProject accession no. PRJNA1398593 (SRX31713286-SRX31713309)

Discussion

26. Please cite the Tables and Figures in the discussion so that readers can easily make the connections and verify the claims.

Response: In discussion, tables and figures were cited in appropriate place.

27. Please ensure all the claims in the discussion are supported by the results.
Response: we revised the discussion based on the revised results and analyses, and all the claims were supported by the results.

Reviewer #2 (Comments for the Author):

Spectrum03246-25
In this article, the authors evaluate a novel assay for the rapid sequencing of coxsackievirus A6 direct from patient specimens using tiling PCR followed by nanopore sequencing. The authors demonstrate good coverage and strain resolution with this assay compared to Illumina sequencing. The authors further discuss potential quality metrics and comment on cost effectiveness of different flow cells.
This manuscript shows what appears to be a solid assay for detection and sequencing of coxsackievirus A6, but unclear language, confusing organization, and unsupported claims detract significantly from the core method described.
Response: We thank the reviewer for the valuable comments and constructive suggestions, which definitely help us to improve the quality and clarity of the article. The shortcomings the reviewer mentioned were carefully addressed and the language, organization and claims were extensively addressed and improved according to the comments.

Major comments:
- Materials and methods section is poorly organized; important information is missing, some information is duplicated, and information that should be presented in the results section is included in the methods section. A non-exhaustive list of examples include redundant description of RNA extraction in sample collection and molecular identification section and cDNA synthesis section, description of overall design (lines 100-102) in the sample collection and molecular identification section, missing description of how samples were selected, missing explanation of how the different cDNA amplification primers were used or how gene-specific primers are defined, use of the term 'depleted' DNA with no discernible depletion step, the sudden introduction of '24' samples from the prior 8 samples (following dilution, presumably), a Figure 1 that is described in the text as showing the Nanopore library prep and sequencing workflow but really shows the overall workflow, sample types (5 throat 3 rectal) in the primer design section, etc.

Response: we sincerely appreciate the valuable comments. After reviewing the relevant parts mentioned, we carefully recomposed the contents according to the reviewer's suggestion. Regarding the poorly organized materials and methods section, the redundant, missing or unclear information was optimized or added.

1) redundant description of RNA extraction: in line 119-121, "200 µl of clinical samples were used to extract RNA using the QIAamp Viral RNA Mini Kit (Qiagen, CA) according to the manufacturer's instructions." was described as methods material. We used same protocol to extract RNA for nanopore and Illumina sequencing.
2) Sample collection and molecular identification: in line 117-119, the sample

collection and molecular identification were specified.

3) cDNA amplification primers: we used 50 µM oligo(dT) for cDNA synthesis. As the protocol of the kits contains three options of primers, we misused all of them in the method description in this study. We have corrected it in line 137-138.

4) Depleted DNA: The purification of the DNA was then further refined through the use of Agencourt AMPure XP beads (Beckman Coulter, Brea, CA, USA) as described in line 161-162. The elution of DNA is standard process according to the instruction. Therefore, "Eluted" is appropriate rather than "Depleted", and we have corrected it in line 163.

5) Origin of the 24 samples: this confusing information was detailed in line 227-233 with Figure 2 supporting it.

6) Figure 1 and workflow: the figure 1 shows overall workflow, which has been moved to supplementary data (Supplementary Fig. 1) and the specific steps of the workflow was provided.

7) Sample types in primer design section: the primer design section was recomposed in line 146-155, experimental samples were not described in this section. Besides, the table of primers was moved to supplementary table S1.

- Paragraph 218-224 is difficult to follow and draws several conclusions that don't appear to be supported by data or if they are, the connections are not clear.
o The authors state that "coverage after dilution was 'relatively poor' (how is that defined?), which was affected by the concentration (Table 2)". However, all but two samples have coverage of at least 0.981 at the highest dilution; all but two undiluted samples have coverage of 0.981 with only two above this value. There are even two instances of the highest dilution samples having better coverage than their undiluted counterparts. Which concentration are the authors talking about? No concentrations are listed in Table 2. How is the second part of this sentence supported by the data?

Response: we thank the reviewer's comment on the data description and confusing conclusions. After extensive discussion on the basis of the sequencing data, we have rewritten this section according to Table 1 and Figure 4 as follows:

"The 24 libraries, derived from serial dilutions of eight CVA6-positive samples, covered a viral load range of Ct 15.54–32.7 (Table 1). All libraries were sequenced on the PromethION flow cell using the established tiling amplicon workflow. The protocol demonstrated high efficiency and speed, achieving an average sequencing depth of >10× within five minutes of run initiation (Figure 4A). High genome coverage (≥95%) was consistently attained across all samples and dilution levels, with most libraries reaching ≥98.1% coverage (Table 1, Figure 4B–I). Notably, for the majority of samples (6/8), the highest dilution (10^{-2}) yielded coverage comparable to

or, in several cases, slightly exceeding that of the undiluted sample, confirming the robustness of the capture protocol across a wide range of template concentrations." (line 262-271)

o "The high Ct value at high concentration might be due to degradation" - which concentration are the authors referring to? Figure 2? It would be difficult for a reader to review Figure 2 and Table 2 side-by-side. The concentration data from Figure 2 could be integrated into Table 2, if this is indeed the case. Either way, looking at Figure 2, there seems to be no clear correlation between Ct value and library concentration (not a huge surprise, in my opinion), and certainly there is no association between high concentrations and high Ct values.

Response: our conclusion of the Ct value at high concentration might be due to degradation was lack of evidence. After thoroughly reviewed and discussed the results, we have modified this part according to the reviewer's comments. A new figure 2 was provided to support the Table 1 that contains core information of the nanopore sequencing. Besides, the library concentration for each sample was provided in Figure 3.

o "The increase in dilution concentration would not lead to sample degradation" - what does 'dilution concentration' mean? What data are the authors referring to when stating that an increase would not lead to sample degradation?

Response: Again, we thank the reviewer for the concern on this issue. The previous expression on this part was confusing and lack of evidence. We have significantly corrected this section to make the result clear, credible, and less likely to cause confusion with Table 1, Figure 2 and 3.

o "The capture efficiency reached up to 90%" - what does capture efficiency refer to? CVA6/Yield? What is the relevance of this number? A sample with only 24% CVA6/Yield has the highest observed genome coverage at 0.991

Response: We thank the reviewer for raising this important point, which allows us to clarify the terminology and interpretation of our data.

"Capture efficiency" in our study refers to the CVA6/Yield ratio – the proportion of sequencing data belonging to the target virus. We will clarify this term in the revision. The higher this ratio, the more efficient and cost-effective the sequencing is. However, complete genome coverage depends primarily on the absolute amount of target data obtained (Table 1), not just the ratio. For example, sample AS217_2021 (10^{-2}) has a CVA6/Yield of only 24.2% but achieved 0.991 coverage because its total output was high enough to yield 37.9 Mb of CVA6-specific data – sufficient for near-complete coverage (Fig. 5B). We have revised the text to state this relationship more clearly (line 280-298).

o "Based on the established CVA6 capture protocol and considering the actual Ct values of the samples, 150 Mb data will definitely enable the acquisition of the near-complete genome sequence of CVA6 from clinical samples" - this sentence makes a definitive conclusion, without providing any data to support that specific cut-off, and basing it on vague language that doesn't explain anything; Nothing in Table 2 correlates Ct values with Yield (is 150 Mb total yield or CVA6-specific yield? Not clear) or yield with coverage; some of this is presented later, but at this point, this is fully unsupported. It is not clear what 'based on the established CVA6 capture protocol' means or how it informs the conclusion drawn.

Response: Thank you for this critical observation. You are correct that the sentence is unsupported and its meaning unclear. We have removed it from the revised manuscript. The specific data volume guidelines are now presented only in the later, data-driven section (currently describing Figure 5). In that section, we clearly state: The recommendations are for total sequencing yield. The target is to obtain a CVA6-specific yield of >50 Mb, which we show is sufficient for ⩾95% coverage (Figure 5B) (line 291-298). The suggested total yields (e.g., ~125 Mb for Ct<25) are calculated by factoring in the expected target reads ratio for each Ct range (Figure 5A). This revision ensures our conclusions are directly tied to the presented data.

- "Data quality and amplification efficiency" section of Results
o "When the Ct value was greater than 28, the percentage of A6 data was below 30%"

- One sample with Ct 29.11 had over 50% CVA6/Yield, one with a Ct of 27.39, had a percentage of 64.5%. How were the 30% (or Ct 28) thresholds chosen as significant? There is certainly a loose correlation between Ct and %CVA6, but the thresholds seem arbitrary.

Response: Thank you for highlighting this important point. You are correct that individual data points (e.g., Ct 29.11 with ~52% CVA6/Yield) deviate from the general trend. The threshold of Ct > 28 and the ~30% target reads ratio were not intended as fixed, significant biological cut-offs, but as practical, conservative estimates for data volume planning based on the observed trendline in Figure 5A. This trend shows a clear negative correlation where higher Ct values generally correspond to lower enrichment efficiency.

We chose these reference points to provide simple, actionable guidance: for samples with the highest Ct values (lowest viral load), users should plan for a larger total sequencing volume to compensate for the expected lower target yield. We will clarify in the revised text that these are guidelines for sequencing depth based on the regression model, not absolute thresholds, and that individual sample performance may vary depending on factors like sample quality and background nucleic acid content. Accordingly, we have revised the text to state this relationship more clearly

(line 291-298).

o In Fig. 4A, it is not clear what is meant by Amplification Efficiency, or which data set goes with the linear regression. The label of "Data quality" in the figure legend is vague.

Response: Thank you for your comment. We agree the original labels and description were unclear. In the revised manuscript and figure, we have made the following adjustments for clarity:

In Figure 5A, the y-axis label has been clarified to indicate that it represents the target reads ratio (CVA6/Yield).

The plot illustrates the observed negative correlation between Ct value and target reads ratio. The fitted trendline is intended to visualize this overall relationship, not to represent a fixed prediction for individual samples, as the data points show natural variation.

We have removed the potentially misleading term "Amplification Efficiency" from the figure and its description. These modifications have been made in the revised manuscript (line 280-289) and figure to ensure the data presentation is accurate and unambiguous.

o "The genomic integrity performance of the genome in the amplified region of A6 reached 97% when the data volume of A6 was greater than 50 Mb (Figure 4)" - First of all Fig. 4B (panels aren't labeled) seems beset by rounding errors; visually, all but 2 samples reached 97%, yet many of those are actually at 96.5%. There only appears to be one sample at <96.5%, which is 95.5%, but multiple 96.5%. I can't figure out which samples were graphed at 96% and 95% - the single 95.5% can only account for one of those, depending on if you round up or down. The threshold of 50 Mb is once again arbitrary - the next two lower volume samples have some of the higher coverage percentages. Overall, the graph seems mostly flat with maybe a slight slope. The decision to use a line to connect the data points in a scatter plot is confusing.

o Thresholds for data volume by Ct value seem arbitrary and not supported by data. This section cites Fig. 4B, but Fig. 4B does not mention Ct values. There is no data to support a minimum data volume of 100-200 Mb for Ct 25-28, and certainly no data to support data volume of >500 Mb for Ct >28; none of the data presented has data volume >215 Mb.

Response: Thank you for the valuable critiques. We have revised the manuscript (line 291-298) to address all concerns.

Figure 5B & Coverage Thresholds: We agree the "97%" claim and 50 Mb threshold were imprecise and arbitrary. The revised text now accurately states that genome

coverage plateaus at ⩾96.5% with a CVA6-specific yield >~20 Mb. The unsupported 50 Mb cutoff and misleading connecting line have been removed.

Data Volume Recommendations: The original Ct-based recommendations (e.g., >500 Mb for Ct>28) were indeed unsupported. They have been removed and replaced with a single, conservative guideline: sequencing to a total yield of 200 Mb reliably provides the CVA6-specific data needed for high coverage. (line 291-298)

Terminology & Interpretation: We have clarified that the term "capture efficiency" refers to the CVA6/Yield ratio (Figure 5A). The manuscript now emphasizes that high absolute CVA6-specific yield is the key determinant of coverage, explaining why samples with lower enrichment ratios can still achieve high coverage.

All conclusions are now precisely supported by the data. We appreciate the reviewer's input.

- "Comparative analysis of amplification efficiency and cost using different flow cells" section
o Line 257 "evaluate the capture efficiency, minimum sequencing data volume" - not clear what this means. Does this refer to the minimum sequencing data volume needed, or the minimum data volume generated at each time point across the various samples?

Response: Thank you for pointing out the lack of clarity in our original phrasing. We have revised the text to precisely state our objectives and methodology.

In the revised manuscript, the relevant section now clearly indicates that we sequenced **the same full set of 24 CVA6 libraries (from 8 samples across three dilutions)** on both MinION and PromethION flow cells for a direct performance comparison. The goal of this comparison was to evaluate the **overall sequencing throughput** (total data output over time) of each platform for this specific assay.

The term **"minimum sequencing data volume"** was ambiguous and has been removed from the revised text. The focus of this comparison is solely on the **aggregate data generation capacity** (throughput) of the two flow cell types, not on establishing a per-sample minimum data requirement. Guidelines for the minimum data volume needed for genome completion are now presented separately and explicitly in the results section, based on the empirical analysis of all samples. (line 308-318)

o Was one sample evaluated? All 24? Only one graph shown for each flow cell, no error bars.

Response: Thank you for this important clarification. In the comparative analysis of

flow cell performance, **all 24 libraries (derived from the 8 original samples across three dilution levels) were evaluated simultaneously on both MinION and PromethION flow cells**. We have clarified this point in the revised figure legend and corresponding text, explicitly stating that the graph illustrates the **total cumulative yield from the 24-plex sequencing run** on each platform, demonstrating the difference in overall throughput between the flow cell types. (Supplementary Fig. S2)

o Line 259f. how was 'genomic integrity' measured? No data shown in Fig 5, only data volume

Response: Thank you for pointing out this discrepancy. We apologize for the unclear terminology. The term **"genomic integrity"** was used imprecisely in that sentence.

In the context of that specific comparison between flow cells, we were referring to the **ability to achieve high (>96.5%) genome coverage**. This metric was not displayed in Figure 5 (now Supplementary Fig. S2), which solely illustrated throughput (data volume over time).

o Authors state that lower cost per GB for PromethION cell makes it more cost effective. Is that scale actually necessary to sequence a reasonable daily volume of samples? Economies of scale only apply at scale.

Response: Thank you for your comment. We agree that the specific cost-per-GB comparison between flow cells is more relevant to large-scale genomic projects than to the practical implementation of our targeted assay for routine surveillance.

We have therefore **removed the detailed cost analysis and the statement regarding "notable cost benefit"** from the revised manuscript. The discussion now simply acknowledges that the **PromethION platform provides higher throughput**, which is a practical consideration for laboratories that need to sequence many samples in parallel to maximize run efficiency. The choice between platforms is framed as a matter of matching **throughput needs and available infrastructure**, rather than a direct cost comparison at scale. (line 412-420)

- "Comparative analysis of CVA6 genomes obtained from Nanopore and Illumina sequencing"
o This section now has 10 samples instead of 8. These additional 2 samples are not described elsewhere. Why were they added/why are they missing from the rest of the analysis?

Response: We thank the reviewer for pointing out the inconsistency of sample numbers and sorry for the confusing information. After establishing the nanopore sequencing for CVA6 using 8 representative clinical samples, additional samples were

also subjected to both nanopore sequencing and Illumina sequencing. In the revised manuscript, we have carefully clarified the samples (n=8) in the methods, results and conclusion for consistency in the revised manuscript.

o The color coding in Fig. 6A could be clearer if the two data points (Illumina and Nanopore) for each sample were clearly identified by using the same color. Country/region is not necessarily required but could be retained by using different symbols. Similarly, Fig. 6B would be easier to read if the same sample had the same color and the different sequencers were identified by different symbols.

Response: According to the reviewer's suggestion, we have modified this Figure (Figure 7) to make it clear to demonstrate the samples in this study and the reference strains.

- Discussion

o L313ff. It is not clear to me that the PromethION has a significant advantage. MinION flow cells reached the 'recommended' data volume, even the 500 Mb, in under 2 hours. The authors suggest that this time difference is critical for informing public health response, but it is not clear in what scenario a difference of under 2 hours would be crucial in this context.

Response: We appreciate the reviewer's astute observation regarding the practical relevance of the time difference between platforms. You are correct that in the context of a typical sequencing run, a difference of under two hours may not critically impact public health decision-making for most surveillance scenarios.

In the revised manuscript, we have adjusted our discussion to better reflect this (line 412-420). We now frame the throughput advantage of the PromethION not as a critical time-saving factor for rapid response, but rather as a practical consideration for laboratory workflow efficiency and scalability. The primary emphasis remains on the assay's overall speed (from sample to result within a single day) and accuracy, which are its key attributes for surveillance.

o The authors claim to have established clear, data driven guidelines for sequencing requirements based on viral load. Based on all of the points mentioned previously, I have to disagree. Viral loads were not established - a Ct value is not a viral load. There is no clear path from the raw data to the recommendations, and certainly no statistics to support these claims.

Response: Thank you for this important clarification. We fully agree that Ct values derived from standard qRT-PCR assays without absolute quantification standards are considered semi-quantitative, rather than fully quantitative, measures of viral load.

We have revised the manuscript accordingly. In the **Methods section** where viral load assessment is described, we now explicitly state that **"Ct values were used as a semi-quantitative measure of viral RNA load." (line 124-125)**

Furthermore, throughout the manuscript, we have been careful to describe the relationship between Ct values and other metrics (e.g., target reads ratio, required data volume) as correlations or trends observed within our experimental system, avoiding any implication of a strict, absolute quantitative relationship.

This adjustment ensures the terminology is accurate and aligns with standard practice in the field.

o Line 322, the authors describe % identity between sequences. Additionally, all 'same' samples from different platforms cluster more closely to each other than to different samples, further supporting that this assay provides sufficient accuracy for relatedness analyis, compared to Illumina.

Response: We thank the reviewer for their positive summary of our comparative genomic analysis. Indeed, our data show that consensus sequences derived from Nanopore and Illumina platforms for the same sample exhibit 99.37%–99.98% identity, confirming high accuracy at the consensus level.

Furthermore, as the reviewer astutely notes, the phylogenetic analysis (Figure 6A) demonstrates that replicate sequences from the same sample consistently cluster together, regardless of the sequencing platform used. This is a strong validation that the assay preserves the true phylogenetic signal and provides sufficient accuracy for robust genotyping and relatedness analysis, which is a primary goal of genomic surveillance. We have highlighted this key point in the revised discussion section (line 390-398).

o Line 328ff., the authors suggest that inconsistencies between Nanopore and Illumina may be due to amplicon drop-out or mapping ambiguities. While possible, the assay as a whole is what it is (and actually seems to perform well in this regard). This is more a limitation than something that can be explained away - and doesn't need to, since the overall performance appears to be just fine.

Response: Thank you for this constructive point. We agree that the observed sequence discrepancies are an inherent characteristic of the comparative performance of the two sequencing methods within our assay, and should be presented as such.

In the revised manuscript, we have reframed this discussion. The text now simply states that the minor inconsistencies observed in the 2A-3C region constitute a known limitation of the comparative analysis when using different platforms and bioinformatic pipelines, rather than attempting to attribute them to a specific technical

cause. We emphasize that the high overall consensus identity (99.37%–99.98%) and the perfect phylogenetic clustering of sample replicates demonstrate that the assay's performance is more than sufficient for accurate genotyping and epidemiological analysis (line 390-398).

o As mentioned above, cost and speed comparison of MinION and PromethION seem mostly academic and not relevant for real-world application of this assay (L335f.)

Response: Thank you for this perspective. We agree that for the specific application of targeted CVA6 sequencing from clinical samples, the performance difference between the flow cells is more relevant than a direct cost/speed comparison at scale.

In the revised manuscript, we have refocused the relevant section. We will retain the observation that PromethION flow cells provide higher overall throughput, which is a practical consideration for laboratories aiming to maximize sample multiplexing per run. However, we have removed the detailed cost-per-GB comparison and the statement about "notable cost benefit," as this is indeed more relevant to large-scale genomics than to the specific, targeted assay presented here. The conclusion will simply state that both platforms are capable of generating complete CVA6 genomes, with the choice between them depending on the user's throughput needs and available instrumentation. (line 309-318)

o L 356ff. "In conclusion, 356 the integration of the wet-lab protocol with automated, user-friendly bioinformatic pipelines for real-time base calling, variant analysis, and phylogenetic tree construction would significantly augment its utility in frontline public health laboratories." This is not a conclusion and does not follow from any of the preceding points.

Response: Thank you for pointing this out. We agree that this statement is speculative and does not logically follow as a conclusion from the data presented in the study. It has been removed from the revised manuscript. The conclusion will now focus solely on summarizing the key validated findings of our work: the sensitivity, accuracy, and practical applicability of the established Nanopore sequencing workflow for CVA6 genomic surveillance directly from clinical samples. (line 434-440)

- Abstract methods section refers to 24 samples - it was 8 (or maybe 10?) samples, plus some dilutions.

Response: We thank the reviewer for pointing out the inconsistency of sample numbers and sorry for the confusing information. After establishing the nanopore sequencing for CVA6 using 8 representative clinical samples, additional samples were subjected to both nanopore sequencing and Illumina sequencing. We have carefully clarified the samples in the methods, results and conclusion for consistency in the

revised manuscript. (Figure 1, Table 1, Figure 6, 7)

- Line 97, Ct values are not quantitative without the appropriate standards and controls. Semi-quantitative at best.

Response: Thank you for this important clarification. We fully agree that Ct values derived from standard qRT-PCR assays without absolute quantification standards are considered semi-quantitative, rather than fully quantitative, measures of viral load.

We have revised the manuscript accordingly. In the **Methods section** where viral load assessment is described, we now explicitly state that **"Ct values were used as a semi-quantitative measure of viral RNA load." (line 124-125)**

Furthermore, throughout the manuscript, we have been careful to describe the relationship between Ct values and other metrics (e.g., target reads ratio, required data volume) as correlations or trends observed within our experimental system, avoiding any implication of a strict, absolute quantitative relationship.

This adjustment ensures the terminology is accurate and aligns with standard practice in the field.

- Figure 1 mentions only throat swabs; graphical abstract mentions throat swabs and feces (using a cup image), method section (under tiling primers design and amplicon) mentions throat and anal (rectal?) swabs

Response: We thank the reviewer for pointing this out. In Supplementary figure S1 (previous Figure 1), we have revised this part to keep consistency with graphical abstract, method section. In this study, two main types of HFMD samples throat swabs and feces were used by scrutiny of information of the used clinical samples.

- Does the bioinformatics section apply to Illumina data or is Illumina analysis not described? If so, add at least a reference and a brief summary.

Response: We thank the reviewer for pointing this out. Bioinformatics of Illumina data was added in methods (line216-224)

- Library construction under the Results section describes a method; results shown are pretty basic (DNA concentrations and gel images) - that's fine but doesn't add much and is not discussed anywhere.

Response: After thoroughly reviewed and discussed the results, we have modified this part according to the reviewer’s comments. A new figure 2 was provided to support the Table 1 that contains core information of the nanopore sequencing.

Besides, the library concentration for each sample was provided in Figure 3.

Minor comments

- Line 58, describe 'atypical' HFMD

Response: we described atypical HFMD (aHFMD) by adding atypical presentations along with aHFMD in line 80-81.

- Line 97, cite if using 'as previously reported'

Response: reference (one of our former studies) was cited.

- Line 99f., what does gradient dilution mean?

Response: through the whole manuscript, serial dilution was used after revision and the description was detailed in results (line 227-233) and figure 2.

- Line 116, abbreviation "GSP" not previously defined

Response: cDNA amplification primers: we used 50 µM oligo(dT) for cDNA synthesis. As the protocol of the kits contains three options of primers including GSP, we misused all of them in the method description in this study. We have corrected it and GSP was removed as it was not used (line 137-138).

- Lines 106-119, the entire section is written in active voice

Response: This section was thoroughly revised and written in passive voice (line 133-144).

- Line 148, cDNA synthesis method has been defined earlier (twice)

Response: the repeated description was removed and cDNA synthesis was detailed in a section.

- Line 157f., 'measured with a Qubit' is colloquial and imprecise

Response: this part was revised as follows: "The DNA libraries were measured using Qubit DNA High Sensitivity (HS) kit in Qubit 4 fluorometer (Thermo Fisher Scientific, USA)" (line 162-163, line 186)

- Line 159f., description of the Bioanalyzer as 'a sophisticated instrument that provides comprehensive analysis of DNA libraries' does not tell the reader what it does exactly or how it was used.

Response: The description was revised and detailed as follows: "The obtained DNA libraries were then subjected to qualitative assessment using an Agilent 2100 Bioanalyzer (Agilent, USA). This instrument employs microfluidic electrophoresis to analyze the fragment size distribution and quality of DNA libraries, and was used here to verify that the library fragment sizes were as expected and to assess overall library

quality." (line 187-191).
- Line 202, what does 'linking the barcodes to the libraries' mean?

Response: this expression was revised to specify the function of barcodes as follows: **"**Prior to loading the libraries into the flow cells, they were linked to the barcoded probes to facilitate identification of each library." (line 255-256).

- Fig 3A is not clearly distinct from Fig 3B-I; consider moving this to a separate figure

Response: We thank the reviewer's suggestion. The previous version was unclear as the curve was too thin and the color was lacking distinctiveness. After optimization, these problems were solved and the new figure (Figure 4) is clear enough with a detailed legend.

- Which sample is shown in Fig 3A? Is there a more legible way to represent coverage over time, maybe a average+range graph over time? Especially at the top end it becomes difficult to distinguish the curves by color.

Response: We appreciate the reviewer's comment. Fig 4A (previous Fig 3A) shows the trends of the average depth data of genomic coverage for all samples in 16 h. This was described in figure legend (line 273-277). Besides, the curve line color was changed and bolded to distinguish each time point.
- Fig 3C: Should this be AS180? AS189 is not mentioned anywhere else.

Response: Thank the reviewer for raising this mistake. We have corrected it in Figure 4C.
- Fig 3B-I: At which time point are these measured?

Response: 16.25 hours
- Some typos (L124 annal swab, L236 dada, L221 Th)

Response: Typos were corrected and the text were proofread by a native English speaker.
- Table 2, showing the barcode number here is not necessary
Response: according to the reviewer's suggestion, the barcode number in table 1 was removed.

Reviewer #3 (Comments for the Author):

Major comments:
1) L250-276: I don't see the value of comparing the same sequencing technology. In addition, the conclusions from this section, ie. PromethION has more output than MinION flow cells and is more cost-effective for bigger data volumes, are disclaimed by Oxford Nanopore technologies. This differences are there by design. In addition

the cost-effectiveness conclusion does not take into account that the MinION flowcell is cheaper by unit, which is still the best choice for lower throughput needs. I also don't see any data on genome integrity in Figure 5. I would suggest to remove this whole section or pivot and compare nanopore sequencing to the Illumina sequencing.
Response: Thank you for these insightful and constructive comments. We agree that the original comparison within the Nanopore ecosystem was of limited value, and the cost-effectiveness conclusion was both overstated and did not account for different throughput needs.

We have significantly revised this section in the manuscript. The detailed, direct comparison of output and cost between MinION and PromethION flow cells has been partially removed and revised as you suggested (line 307-317). Instead, we have integrated a more balanced and practical discussion of platform choice into the main Discussion section, with a pivot towards the **overall value proposition of our Nanopore workflow in comparison to the standard (Illumina)**. (line 411-419)

The revised text now simply states that the assay was validated using both MinION (on a GridION device) and PromethION flow cells, and that both successfully generated complete genomes. The focus is then shifted to the core advantages of our Nanopore protocol as a whole: its **speed (same-day turnaround from sample to consensus)**, **accuracy (high concordance with Illumina)**, and **direct applicability to clinical specimens**. The choice of flow cell is framed not as a primary conclusion, but as a flexible implementation detail that laboratories can decide based on their existing infrastructure and desired run scale.

We believe this revision addresses your concerns by removing the unnecessary internal comparison, avoiding speculative cost claims, and refocusing the narrative on the validated performance and practical utility of the method compared to established techniques.

2) I'm missing some discussion on why the region between 5000-6000 nucleotides consistently has a low coverage (Figure3), what could the reasons be and how could they be solved? Is it a region where the primers do not easily bind (eg. secondary structures) or is this because this region is more divergent from the strain the primers were designed on, etc.? Certainly because this does have an impact on the genome assembly as shown in Figure 6 with the most differences between Nanopore and Illumina in this region.

Response: Thank you for this insightful observation regarding sample AS377_2023. Indeed, as noted in our results (Figure 3I), this sample exhibited more uniform and higher sequencing depth, particularly across the 5500–6000 nt region where other samples showed a coverage dip.

The specific biological or technical factors leading to its superior performance cannot be definitively determined from our current data. However, we can propose plausible explanations consistent with our overall findings:

The few observed nucleotide discrepancies were not randomly distributed but were overwhelmingly concentrated within the genomic region spanning approximately 3.5–6.0 kb, which encompasses parts of the non-structural 2A-3C genes (Figure 6B). This interval corresponds precisely to a zone of lower and more variable sequencing depth in most samples (Figure 4B-H) and is a well-documented recombination hotspot and region of heightened genetic diversity in enteroviruses, including CVA6(4, 15, 21, 50, 51). Therefore, the minor inconsistencies are more likely attributable to the combined challenges of amplifying and unambiguously mapping this highly variable region with a fixed primer set, rather than to a systematic error of the Nanopore platform. The exceptional performance of sample AS377 in this region—achieving 100% identity between platforms and uniform depth (Figure 4I)—supports this interpretation, indicating that under conditions of optimal primer match and coverage, perfect concordance is attainable. (line 398-410)

Minor comments:

L69-70: PromethION is not a portable sequencer, so it does not represent a significant advancement in portable sequencing technology. I think you mean nanopore technology in general or MinION.

Response: “Nanopore sequencing technology” was used rather than PromethION in line 90.

L82: nt identical -> nucleotide identity

Response: corrected in line 104.

L83: sequencing -> sequencing protocol

Response: corrected in line 105.

L107-108: the first sentence seems redundant + the methods section should not be a protocol unless it is a protocol paper, I suggest to write out a detailed protocol in the supplementary data and keep the method description simple here and remove too detailed descriptions (eg. "Chill on ice. Store the cDNA synthesis reaction at -20{degree sign}C, or proceed directly to PCR.")

Response: We thank the reviewer to raise this issue. A supplementary data including detailed protocol (supplementary Figure S1, Table S1-S3) was provided and too detailed descriptions were removed.

L170-190: Most of the used bioinformatic tools also have published papers, provided these as references instead of links where possible.

Response: we appreciate this important suggestion, and references for bioinformatic tools were cited and the links were removed.

L202-203: I would suggest to remove this sentence as it belongs more in the method section

Response: It was removed and this part was rewritten.

L210: 10-time -> 10-fold, 10-time might be interpreted as 10 dilutions

Response: It was corrected in line 231 and through the whole manuscript.

L218-219: the authors claim a poor coverage for high Ct samples but in the table they reference there is not much difference in the coverage between the dilutions. In additions, what does this coverage mean, depth/horizontal coverage? Please clarify and add a unit to the coverage column.

Response: Thank you for the valuable critiques. We have revised the manuscript to address all concerns as follows:

"The 24 libraries, derived from serial dilutions of eight CVA6-positive samples, covered a viral load range of Ct 15.54–32.7 (Table 1). All libraries were sequenced on the PromethION flow cell using the established tiling amplicon workflow. The protocol demonstrated high efficiency and speed, achieving an average sequencing depth of >10× within five minutes of run initiation (Figure 4A). High genome coverage (≥95%) was consistently attained across all samples and dilution levels, with most libraries reaching ≥98.1% coverage (Table 1, Figure 4B–I). Notably, for the majority of samples (6/8), the highest dilution (10^{-2}) yielded coverage comparable to or, in several cases, slightly exceeding that of the undiluted sample, confirming the robustness of the capture protocol across a wide range of template concentrations." (line 262-270)

As for the coverage, it means the horizontal coverage (genome coverage), which is added in the column.

L219-221: I'm not sure what the authors try to show with the sentences about sample degradation. + where should I see that the capture efficiency in Table2?

Response: We thank the reviewer for raising this important point, which allows us to clarify the terminology and interpretation of our data.

"Capture efficiency" in our study refers to the CVA6/Yield ratio – the proportion of sequencing data belonging to the target virus. We will clarify this term in the revision. The higher this ratio, the more efficient and cost-effective the sequencing is. However, complete genome coverage depends primarily on the absolute amount of target data obtained (Table 1), not just the ratio. For example, sample AS217_2021 (10^{-2}) has a CVA6/Yield of only 24.2% but achieved 0.991 coverage because its total output was high enough to yield 37.9 Mb of CVA6-specific data – sufficient for near-complete

coverage (Fig. 5B). We have revised the text to state this relationship more clearly (line 279-289).

Our conclusion of the Ct value at high concentration might be due to degradation was lack of evidence. After thoroughly reviewed and discussed the results, we have modified this part to avoid using vague language according to the reviewer's comments.

L242-243: The authors claim that a data volume of 500Mb is enough to ensure genome-wide-coverage for samples with a Ct > 28, but the 500Mb is never shown in their data analyses?

Response: It should be 50Mb, we have corrected this mistake in line 292.

L285: In the rest of the manuscript only 8 samples are mentioned?

Response: We thank the reviewer for pointing out the inconsistency of sample numbers and sorry for the confusing information. After establishing the nanopore sequencing for CVA6 using 8 representative clinical samples, additional samples were subjected to both nanopore sequencing and Illumina sequencing. We have carefully clarified the samples in the methods, results and conclusion for consistency in the revised manuscript.

L295: Is there something special about sample AS377 that it performs better than the other samples?

Response: As discussed**,** the superior performance of sample AS377 in this region may be attributed to its closer sequence homology to the primer set or more favorable local sequence context for amplification (line 407-409). Besides, the viral RNA (lower Ct value) in this sample may have been better preserved, or its specific genomic sequence in the variable 3C region (hotspot of recombination) may be less prone to forming stable secondary structures that hinder polymerase processivity during PCR—a common challenge in amplicon-based sequencing.

Figure 3A: Is this across a particular sample or are all samples pooled? Please clarify.

Response: We appreciate the reviewer's comment. Fig 4A (previous Fig 3A) shows the trends of the average depth data of genomic coverage for all samples in 16 h. This was described in figure legend (line 274-275). Besides, the curve line color was changed and bolded to distinguish each time point.

Figure 4: What do the blue and red dotted lines mean in this plot? What do the point

shapes mean in panel A?

Response: Thank you for your comment. We agree the original labels and description were unclear. In the revised manuscript and figure, we have made the following adjustments for clarity:

In Figure 5A, the y-axis label has been clarified to indicate that it represents the target reads ratio (CVA6/Yield). The plot illustrates the observed negative correlation between Ct value and target reads ratio. The fitted trendline is intended to visualize this overall relationship, not to represent a fixed prediction for individual samples, as the data points show natural variation.

We have removed the potentially misleading term "Amplification Efficiency" from the figure and its description. These modifications have been made in the revised manuscript (line 279-297) and figure to ensure the data presentation is accurate and unambiguous.

Figure 6: what does the scale in panel A represent?

Response: the scale bar which refers to phylogenetic distance has been detailed in the figure legend of Figure 6A and figure 7.

Re: Spectrum03246-25R1 (Targeted sequence capture of coxsackievirus A6 using Nanopore sequencing directly from clinical specimens)

Dear Dr. Hui Li:

Thank you for substantially revising your manuscript and providing point-by-point responses to the reviewers. Below you will find my comments, instructions from the Spectrum editorial office, and the reviewer comments.

While the reviewers noted significant improvements in the manuscript's clarity and flow, they identified outstanding concerns for further refinements in the Methods, Results, data volume conclusion, and the presentation of data in Figure 5. Additionally, per ASM Data Availability policy, all sequences deposited to GenBank must be made publicly accessible; please ensure these are released, as currently only the SRA reads are available.

Please return the manuscript within 60 days; if you cannot complete the modification within this time period, please contact me. If you do not wish to modify the manuscript and prefer to submit it to another journal, notify me immediately so that the manuscript may be formally withdrawn from consideration by Spectrum.

Revision Guidelines
To submit your modified manuscript, log into the submission site at https://spectrum.msubmit.net/cgi-bin/main.plex. Go to Author Tasks and click the appropriate manuscript title to begin. The information you entered when you first submitted the paper will be displayed; update this as necessary. Note the following requirements:

• Upload point-by-point responses to the issues raised by the reviewers in a file named "Response to Reviewers," NOT in your cover letter.
• Upload a compare copy of the manuscript (without figures) as a "Marked-Up Manuscript" file.
• Upload a clean .DOC/.DOCX version of the revised manuscript and remove the previous version.
• Each figure must be uploaded as a separate, editable, high-resolution file (TIFF or EPS preferred), and any multipanel figures must be assembled into one file.
• Any supplemental material intended for posting by ASM should be uploaded with their legends separate from the main manuscript. You can combine all supplemental material into one file (preferred) or split it into a maximum of 10 files with all associated legends included.

For complete guidelines on revision requirements, see our Submission and Review Process webpage. Submission of a paper that does not conform to guidelines may delay acceptance of your manuscript.

Data availability: ASM policy requires that data be available to the public upon online posting of the article, so please verify all links to sequence records, if present, and make sure that each number retrieves the full record of the data. If a new accession number is not linked or a link is broken, provide Spectrum production staff with the correct URL for the record. If the accession numbers for new data are not publicly accessible before the expected online posting of the article, publication may be delayed; please contact production staff (Spectrum@asmusa.org) immediately with the expected release date.

Publication Fees: For information on publication fees and which article types are subject to charges, visit our website. If your manuscript is accepted for publication and any fees apply, you will be contacted separately about payment during the production process; please follow the instructions in that e-mail. Arrangements for payment must be made before your article is published.

ASM Membership: Corresponding authors may join or renew ASM membership to obtain discounts on publication fees. Need to upgrade your membership level? Please contact Customer Service at Service@asmusa.org.

The ASM Journals program strives for constant improvement in our submission and publication process. Please tell us how we can improve your experience by taking this quick Author Survey.

Thank you for submitting your paper to Spectrum.

Sincerely,
My Phan
Editor
Microbiology Spectrum

Reviewer #1 (Comments for the Author):

1. As per recommended nomenclature, please use CVA6 and not CV-A6 throughout the manuscript. Kindly read the article

below for more specific guidance. "Simmonds et al., 2020. Recommendations for the nomenclature of enteroviruses and rhinoviruses. Arch Virol. 2020 Mar;165(3):793-797. doi: 10.1007/s00705-019-04520-6."

2. The authors still did not show irrefutable evidence that the samples did not contain other enteroviruses and especially members of the EV-A species.
3. Figure 4: Please add schematic representation of genome architecture to panels A -I. This will help readers better understand what part of the genome the different contigs represent.
4. Lines 77-80: Please cite the source of the enterovirus (EV) typing criteria described here.
5. Lines 148-149: What was used for cDNA; Virus specific primers or oligo(dT) or both? Please be as clear as possible to ensure readers can independently reproduce the method.
6. Lines 175-185: Two different ONT library prep kits and workflows were mentioned. Which was used and for what. Please be as clear as possible to ensure readers can independently reproduce the method.
7. Lines 205-226: The are quite a few red flags in this region

a. Demultiplexing was first done using Guppy. Why do it again with Porechop?
b. What does this mean: "The software for enterovirus reference genomes was obtained from the NCBI website (https://www.ncbi.nlm.nih.gov/) for analysis"? What software are you referring to?
c. You have already assembled the genome using Flye. How was the genome polished? What do you need Freebayes and Bcftools for after you already have your assembled genome from Flye and have contigs? Please be very clear about your methods section.
d. Lines 78-79 already mentioned the typing criteria [>75% similarity in the VP1 genomic region]. What do you mean by "optimal genome selection in lines 218-219. Shouldn't the contigs assembled using Flye for each sample be your reference for each sample should you want to estimate target to total reads ratio?
e. Mapping to a reference you see fit (or consider optimal) based on BLASTn hits can force the similarity found in figures 6 and 7.
f. No recombination analysis was carried out to rule out potential recombination events (Chimeras). Furthermore, no nucleotide substitution model testing was done and phylogenetic trees were not constructed to test P1 vs P2 or P1 vs P2-P3 to rule out recombination. The observation with AS289G2 vs AS289G3 in Figure 6C strongly suggest the need for recombination analysis.
g. Note that the supplementary data [see image below] shows evidence that suggest other EV-As like EV-A71 was present in some samples. Please carefully reexplore the data.

8. Table 1: For easier reading, if possible, kindly express the values in the columns CVA6/Yield and Genome coverage as percentages. This will make it easier for readers to connect the text and the table. For example, in lines 298-300, you mention 99.1% but in Table 1, the value is 0.991. Having both as percentages makes the connection easier for the reader. Also please site Table 1 on line 300.
9. Figure 6A: There are 18 sequences instead of 16 as mentioned in the text. There are two sequences in the tree that stand out because they are represented with accession numbers. The figure details mention they are the top 2 BLASTn hits. Why are they present in 6A and 6B but not in 6C. Do you mean all 16 sequences [with divergence approaching 6% according to figure 6B] have these same 2 sequences as their top 2 hits in GenBank?
10. Please mention the tool used for Figure 6B in the bioinformatics subsection of the methods section.
11. There is no table similar to Table 1 describing the statistics for the data generated for Illumina sequencing. Please add the data. The Illumina data also needs to be de-novo assembled and subjected to recombination analysis. Furthermore, if possible, kindly mention if the varying region of AS289G2 vs AS289G3 highlighted in Figure 6C was re-sequenced using the sanger technology. In addition, is that variation still present if both sequences [AS289G2 and AS289G3] were de-novo assembled?
12. Line 350: "2A-3C genomic region (approximately 3500-6000 nucleotides)"

a. Do you really mean (approximately 3500-6000 nucleotides) or (approximately nucleotides 3500-6000)?
b. The region with lower sequencing depth in Figure 4B-H is generally between nucleotides 5000 and 7500 not 3500-6000. Please re-examine the data.
c. As opposed to line 353, nucleotides 5000 to 7500 encode nonstructural proteins and these usually do not have higher sequence diversity. Usually it is the P1 region (which encodes structural proteins) that has higher sequence diversity.
d. Please add a schematic representation of genome architecture to panels A -I. This will help readers better understand what part of the genome the different contigs represent.

13. Please make adjustments throughout the discussion section based on the comments above.
14. Lines 436-439: PCR amplification should make it easier to detect minority variants. Please check literature on this and be more precise on what you mean.

Reviewer #2 (Comments for the Author):

The authors performed a significant rewrite of the manuscript, and I now find it to be much improved. It is well-structured and easy to read and follow. Figures are helpful, and conclusions are now mostly supported by the data. A few issues remain to be addressed.

I cannot arrive at the same conclusion as the authors as it relates to the data volume recommendations. Despite stating that it was removed as unsupported in the responses to reviewer comments, the authors retain the 50 Mb cutoff on line 297 and 303.

In fact, the authors state that a CVA6 data volume of >50 Mb reliably resulted in >95% genome coverage. In fact, every sample tested resulted in >95% genome coverage. For all the data supports, 13.5 Mb CVA6 reliably resulted in 95% genome coverage, and for all we know, the number may be lower than that. The yield recommendations are based on this arbitrary 50 Mb cut-off and therefore equally unsupported. The chosen brackets are also arbitrary as they could be set anywhere along the Ct vs reads ratio regression line (say for example Ct 22 - TRR ~60% - min yield 85 Mb) and having a lower recommendation for Ct >28 than the upper range for Ct 25-28 makes no sense. Further, the 'correlation' between CVA6 data volume and genomic coverage is minimal and unproven. No statistical tests were applied to the correlation, and visually, the slope of the regression line would be small with significant residuals on either side. Lastly, the discussion in 394-398 is based on this data, which in my view does not support the conclusion. The following changes should be made to eliminate these unsupported guidelines:
• Remove all cut-off (horizontal and vertical) lines in Fig. 5A and 5B
• Remove all language suggesting that a specific data volume results in a specific amount of genomic coverage (including complete or 95%) unless appropriately supported by statistical tests
• Remove all language based on unproven CVA6 data volume cut-offs. This includes recommendations of certain total sequencing yields based on Ct value in the abstract, results, and discussion sections
This ultimately results in the removal of the entirety of the data volume and read ratio section of the results (and affiliated discussion on line 394-398, as well as the abstract), yet the data itself is worth presenting. In my view, the following conclusions can be drawn from the data as presented:
• A lower Ct value results in a higher CVA6 data volume yield and a higher target reads ratio
• There may be (do NOT be definitive unless supported with statistical analysis) a correlation between CVA6 data volume and genomic coverage
• Together, these data suggest that a minimum amount of CVA6 data volume may be necessary to achieve complete coverage and that higher Ct value samples may have to be sequenced to higher total sequencing yield to achieve complete (or near complete) coverage
• It may be possible to establish minimum sequencing data volume guidance either independently or based on Ct, which may result in more efficient resource allocation

Finally, the data in Fig 5B appears to be incorrect. As stated in my previous comments, all genome coverage data is shown in complete percentage points, whereas actual data is resolved to 0.1%. This leads to an entirely inaccurate plot. As stated previously, there is one data point at 95% - this must be the lowest data point at 95.5%. However, there is also only 1 data point at 96%, but the 'next highest' data point in the table is 96.5%, of which there are 4 in the table. The lack of attention to detail in this figure, and the lack of correction based on prior comments, is concerning.

The continued use of the term 'viral load' when referring to Ct values remains inaccurate. A Ct value is not a viral load, not even a semi-quantitative one. A Ct value is a semi-quantitative value correlating with a viral load. A viral load is a specific term and while we can measure viral loads by calculating backwards from the Ct value, that does not make a Ct value a viral load. A bank account's interest yield is directly proportional to the account balance, yet it is not in fact the account balance, though it may be calculated from it. This may seem like a petty point, but the conflation of Ct values with viral loads continues to perpetuate false equivalency and misuse of Ct values in the infectious disease diagnostic field. Please remove all instances of calling a Ct value a viral load. Where needed for clarity, it would be appropriate to use phrases such as "lower Ct values, suggesting higher viral load" or similar.

Minor points:
• Line 126 still refers to anal swabs - this does not appear to be a sample type that was used
• Please remove the connecting line from the scatter plot in Fig. 5B.
• In the text describing Fig. 6A/B, please describe the purpose of the additional 2 isolates from GenBank - are those there as an outgroup? What is the reader supposed to learn from their inclusion?

Reviewer #3 (Comments for the Author):

The manuscript has improved a lot since first submission, I do have a few minor comments left:

L205: What is the name of the software for 'enterovirus reference genomes obtained from NCBI)'?
L220: add reference for iVar (https://link.springer.com/article/10.1186/s13059-018-1618-7)
L285-289: From Table 1 and Figure 5B it seems that all samples got a high coverage regardless of the CVA6 data volume, why the recommendation for >50Mb and not >10Mb as this is the lowest data volume that gave a high coverage?
L384-388: What would be the recommendations to get to these data volumes when you have a set of samples that you want to multiplex but they have different Ct values (some >28 and some <25)? Pool them into non-equimolar amounts?
Figure 5B: It is still not entirely clear to me why there is a blue dotted line on y=0.97, is this the minimum coverage chosen to represent high coverage and therefore you chose to visualize this?
Also in the figure description for Figure 5A and B, "The intersection points of the red dotted line and the blue dotted line lie on the fitted straight line." and "The intersection points of the red dotted line and the blue dotted line lie on the lowest genomic coverage point." do not actually describe what these lines represent.
For figure 5A, the red dotted lines indicate the Ct value cut offs and the blue dotted lines the chosen target reads ratio for each

Ct. But this is not clear from the description above, so please edit the description.
Figure 6 and 7: change "phylogenetic distance" to "nucleotide substitutions/site" as this is what the scale bar is actually representing

Reviewer Comments

Manuscript number: Spectrum03246-25-R1

Title: Targeted sequence capture of coxsackievirus A6 using Nanopore sequencing directly from clinical specimens

Summary and Recommendation

The authors have significantly improved the manuscript. However, some red flags remain [especially in the methods and results section] that need to be addressed before publication. **The study deserves to be published after these red flags have been resolved.**

General Comments

1. As per recommended nomenclature, please use CVA6 and not CV-A6 throughout the manuscript. Kindly read the article below for more specific guidance. "*Simmonds et al., 2020. Recommendations for the nomenclature of enteroviruses and rhinoviruses. Arch Virol. 2020 Mar;165(3):793-797. doi: 10.1007/s00705-019-04520-6.*"
2. The authors still did not show irrefutable evidence that the samples did not contain other enteroviruses and especially members of the EV-A species.
3. Figure 4: Please add schematic representation of genome architecture to panels A -I. This will help readers better understand what part of the genome the different contigs represent.
4. Lines 77-80: Please cite the source of the enterovirus (EV) typing criteria described here.
5. Lines 148-149: What was used for cDNA; Virus specific primers or oligo(dT) or both? Please be as clear as possible to ensure readers can independently reproduce the method.
6. Lines 175-185: Two different ONT library prep kits and workflows were mentioned. Which was used and for what. Please be as clear as possible to ensure readers can independently reproduce the method.
7. Lines 205-226: The are quite a few red flags in this region
    a. Demultiplexing was first done using Guppy. Why do it again with Porechop?
    b. What does this mean: "*The software for enterovirus reference genomes was obtained from the NCBI website (https://www.ncbi.nlm.nih.gov/) for analysis*"? What software are you referring to?
    c. You have already assembled the genome using Flye. How was the genome polished? What do you need Freebayes and Bcftools for after you already have your assembled genome from Flye and have contigs? Please be very clear about your methods section.
    d. Lines 78-79 already mentioned the typing criteria [>75% similarity in the VP1 genomic region]. What do you mean by "optimal genome selection in lines 218-219. Shouldn't the contigs assembled using Flye for each sample be your reference for each sample should you want to estimate target to total reads ratio?
    e. Mapping to a reference you see fit (or consider optimal) based on BLASTn hits can force the similarity found in figures 6 and 7.
    f. No recombination analysis was carried out to rule out potential recombination events (Chimeras). Furthermore, no nucleotide substitution model testing was done and phylogenetic trees were not constructed to test P1 vs P2 or P1 vs P2-P3 to rule

out recombination. The observation with AS289G2 vs AS289G3 in Figure 6C strongly suggest the need for recombination analysis.

g. Note that the supplementary data [see image below] shows evidence that suggest other EV-As like EV-A71 was present in some samples. Please carefully reexplore the data.

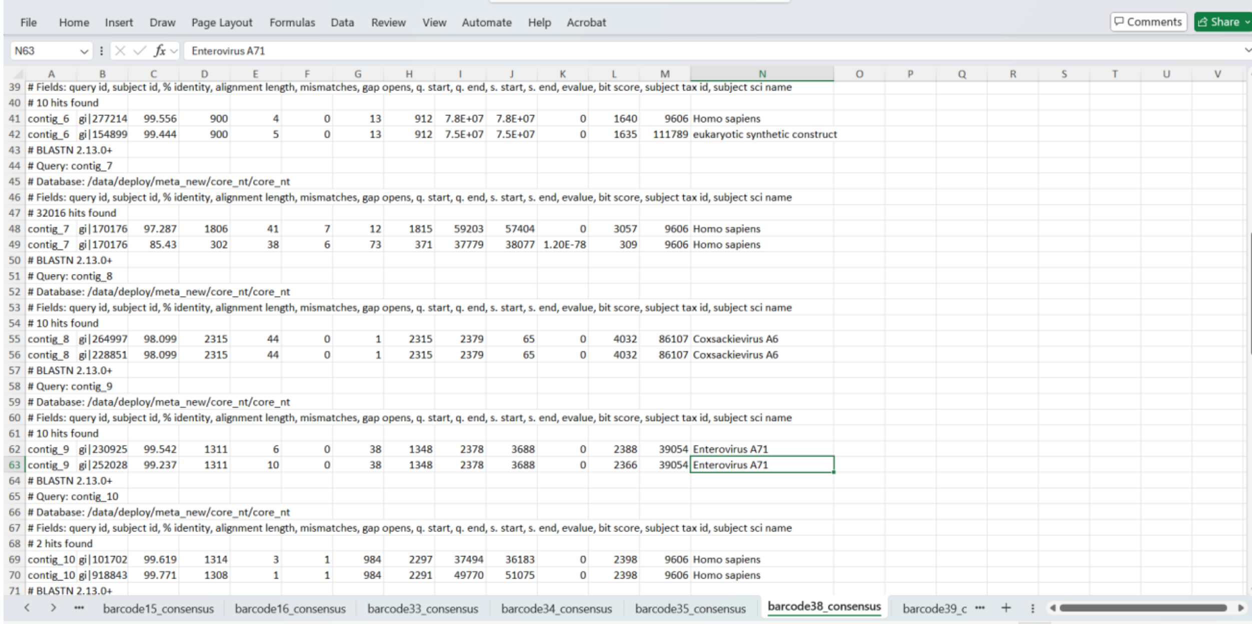

	A	B	C	D	E	F	G	H	I	J	K	L	M	N
39	# Fields: query id, subject id, % identity, alignment length, mismatches, gap opens, q. start, q. end, s. start, s. end, evalue, bit score, subject tax id, subject sci name													
40	# 10 hits found													
41	contig_6	gi|277214	99.556	900	4	0	13	912	7.8E+07	7.8E+07	0	1640	9606	Homo sapiens
42	contig_6	gi|154899	99.444	900	5	0	13	912	7.5E+07	7.5E+07	0	1635	111789	eukaryotic synthetic construct
43	# BLASTN 2.13.0+													
44	# Query: contig_7													
45	# Database: /data/deploy/meta_new/core_nt/core_nt													
46	# Fields: query id, subject id, % identity, alignment length, mismatches, gap opens, q. start, q. end, s. start, s. end, evalue, bit score, subject tax id, subject sci name													
47	# 32016 hits found													
48	contig_7	gi|170176	97.287	1806	41	7	12	1815	59203	57404	0	3057	9606	Homo sapiens
49	contig_7	gi|170176	85.43	302	38	6	73	371	37779	38077	1.20E-78	309	9606	Homo sapiens
50	# BLASTN 2.13.0+													
51	# Query: contig_8													
52	# Database: /data/deploy/meta_new/core_nt/core_nt													
53	# Fields: query id, subject id, % identity, alignment length, mismatches, gap opens, q. start, q. end, s. start, s. end, evalue, bit score, subject tax id, subject sci name													
54	# 10 hits found													
55	contig_8	gi|264997	98.099	2315	44	0	1	2315	2379	65	0	4032	86107	Coxsackievirus A6
56	contig_8	gi|228851	98.099	2315	44	0	1	2315	2379	65	0	4032	86107	Coxsackievirus A6
57	# BLASTN 2.13.0+													
58	# Query: contig_9													
59	# Database: /data/deploy/meta_new/core_nt/core_nt													
60	# Fields: query id, subject id, % identity, alignment length, mismatches, gap opens, q. start, q. end, s. start, s. end, evalue, bit score, subject tax id, subject sci name													
61	# 10 hits found													
62	contig_9	gi|230925	99.542	1311	6	0	38	1348	2378	3688	0	2388	39054	Enterovirus A71
63	contig_9	gi|252028	99.237	1311	10	0	38	1348	2378	3688	0	2366	39054	Enterovirus A71
64	# BLASTN 2.13.0+													
65	# Query: contig_10													
66	# Database: /data/deploy/meta_new/core_nt/core_nt													
67	# Fields: query id, subject id, % identity, alignment length, mismatches, gap opens, q. start, q. end, s. start, s. end, evalue, bit score, subject tax id, subject sci name													
68	# 2 hits found													
69	contig_10	gi|101702	99.619	1314	3	1	984	2297	37494	36183	0	2398	9606	Homo sapiens
70	contig_10	gi|918843	99.771	1308	1	1	984	2291	49770	51075	0	2398	9606	Homo sapiens
71	# BLASTN 2.13.0+													

8. **Table 1:** For easier reading, if possible, kindly express the values in the columns CVA6/Yield and Genome coverage as percentages. This will make it easier for readers to connect the text and the table. For example, in lines 298-300, you mention 99.1% but in Table 1, the value is 0.991. Having both as percentages makes the connection easier for the reader. Also please site Table 1 on line 300.
9. **Figure 6A:** There are 18 sequences instead of 16 as mentioned in the text. There are two sequences in the tree that stand out because they are represented with accession numbers. The figure details mention they are the top 2 BLASTn hits. Why are they present in 6A and 6B but not in 6C. Do you mean all 16 sequences [with divergence approaching 6% according to figure 6B] have these same 2 sequences as their top 2 hits in GenBank?
10. Please mention the tool used for Figure 6B in the bioinformatics subsection of the methods section.
11. There is no table similar to Table 1 describing the statistics for the data generated for Illumina sequencing. Please add the data. The Illumina data also needs to be *de-novo* assembled and subjected to recombination analysis. Furthermore, if possible, kindly mention if the varying region of AS289G2 vs AS289G3 highlighted in Figure 6C was re-sequenced using the sanger technology. In addition, is that variation still present if both sequences [AS289G2 and AS289G3] were *de-novo* assembled?
12. Line 350: "2A-3C genomic region (approximately 3500-6000 nucleotides)"
    a. Do you really mean (approximately 3500-6000 nucleotides) or (approximately nucleotides 3500-6000)?
    b. The region with lower sequencing depth in Figure 4B-H is generally between nucleotides 5000 and 7500 not 3500-6000. Please re-examine the data.

c. As opposed to line 353, nucleotides 5000 to 7500 encode nonstructural proteins and these usually do not have higher sequence diversity. Usually it is the P1 region (which encodes structural proteins) that has higher sequence diversity.
d. Please add a schematic representation of genome architecture to panels A -I. This will help readers better understand what part of the genome the different contigs represent.

13. Please make adjustments throughout the discussion section based on the comments above.
14. Lines 436-439: PCR amplification should make it easier to detect minority variants. Please check literature on this and be more precise on what you mean.

Spectrum03246-25 R1

The authors performed a significant rewrite of the manuscript, and I now find it to be much improved. It is well-structured and easy to read and follow. Figures are helpful, and conclusions are now mostly supported by the data. A few issues remain to be addressed.

I cannot arrive at the same conclusion as the authors as it relates to the data volume recommendations. Despite stating that it was removed as unsupported in the responses to reviewer comments, the authors retain the 50 Mb cutoff on line 297 and 303. In fact, the authors state that a CVA6 data volume of >50 Mb reliably resulted in >95% genome coverage. In fact, every sample tested resulted in >95% genome coverage. For all the data supports, 13.5 Mb CVA6 reliably resulted in 95% genome coverage, and for all we know, the number may be lower than that. The yield recommendations are based on this arbitrary 50 Mb cut-off and therefore equally unsupported. The chosen brackets are also arbitrary as they could be set anywhere along the Ct vs reads ratio regression line (say for example Ct 22 – TRR ~60% - min yield 85 Mb) and having a lower recommendation for Ct >28 than the upper range for Ct 25-28 makes no sense. Further, the 'correlation' between CVA6 data volume and genomic coverage is minimal and unproven. No statistical tests were applied to the correlation, and visually, the slope of the regression line would be small with significant residuals on either side. Lastly, the discussion in 394-398 is based on this data, which in my view does not support the conclusion. The following changes should be made to eliminate these unsupported guidelines:

- Remove all cut-off (horizontal and vertical) lines in Fig. 5A and 5B
- Remove all language suggesting that a specific data volume results in a specific amount of genomic coverage (including complete or 95%) unless appropriately supported by statistical tests
- Remove all language based on unproven CVA6 data volume cut-offs. This includes recommendations of certain total sequencing yields based on Ct value in the abstract, results, and discussion sections

This ultimately results in the removal of the entirety of the data volume and read ratio section of the results (and affiliated discussion on line 394-398, as well as the abstract), yet the data itself is worth presenting. In my view, the following conclusions can be drawn from the data as presented:

- A lower Ct value results in a higher CVA6 data volume yield and a higher target reads ratio
- There may be (do NOT be definitive unless supported with statistical analysis) a correlation between CVA6 data volume and genomic coverage

- Together, these data suggest that a minimum amount of CVA6 data volume may be necessary to achieve complete coverage and that higher Ct value samples may have to be sequenced to higher total sequencing yield to achieve complete (or near complete) coverage
- It may be possible to establish minimum sequencing data volume guidance either independently or based on Ct, which may result in more efficient resource allocation

Finally, the data in Fig 5B appears to be incorrect. As stated in my previous comments, all genome coverage data is shown in complete percentage points, whereas actual data is resolved to 0.1%. This leads to an entirely inaccurate plot. As stated previously, there is one data point at 95% - this must be the lowest data point at 95.5%. However, there is also only 1 data point at 96%, but the 'next highest' data point in the table is 96.5%, of which there are 4 in the table. The lack of attention to detail in this figure, and the lack of correction based on prior comments, is concerning.

The continued use of the term 'viral load' when referring to Ct values remains inaccurate. A Ct value is not a viral load, not even a semi-quantitative one. A Ct value is a semi-quantitative value correlating with a viral load. A viral load is a specific term and while we can measure viral loads by calculating backwards from the Ct value, that does not make a Ct value a viral load. A bank account's interest yield is directly proportional to the account balance, yet it is not in fact the account balance, though it may be calculated from it. This may seem like a petty point, but the conflation of Ct values with viral loads continues to perpetuate false equivalency and misuse of Ct values in the infectious disease diagnostic field. Please remove all instances of calling a Ct value a viral load. Where needed for clarity, it would be appropriate to use phrases such as "lower Ct values, suggesting higher viral load" or similar.

Minor points:

- Line 126 still refers to anal swabs – this does not appear to be a sample type that was used
- Please remove the connecting line from the scatter plot in Fig. 5B.
- In the text describing Fig. 6A/B, please describe the purpose of the additional 2 isolates from GenBank – are those there as an outgroup? What is the reader supposed to learn from their inclusion?

Point-by-point response

Re: Spectrum03246-25R1 (Targeted sequence capture of coxsackievirus A6 using Nanopore sequencing directly from clinical specimens)

Dear Dr. Hui Li:

Thank you for substantially revising your manuscript and providing point-by-point responses to the reviewers. Below you will find my comments, instructions from the Spectrum editorial office, and the reviewer comments.

While the reviewers noted significant improvements in the manuscript's clarity and flow, they identified outstanding concerns for further refinements in the Methods, Results, data volume conclusion, and the presentation of data in Figure 5. Additionally, per ASM Data Availability policy, all sequences deposited to GenBank must be made publicly accessible; please ensure these are released, as currently only the SRA reads are available.

Please return the manuscript within 60 days; if you cannot complete the modification within this time period, please contact me. If you do not wish to modify the manuscript and prefer to submit it to another journal, notify me immediately so that the manuscript may be formally withdrawn from consideration by Spectrum.

Response: Dear Dr. Phan, editor of *Microbiology Spectrum*, thank you so much for being in charge of the second-round peer review, which again provides us valuable and constructive comments for further quality improvement of this manuscript. After carefully abstracting concerns on Methods, Results, figures and data, we have addressed all of them point by point to the fullest extent possible. Moreover, raw data of sequencing and supplementary sanger sequencing (new data for review) for sample AS289 were provided. Besides, sequences deposited to GenBank have been released to public. We sincerely appreciate your effort and time in the article assessment, and hope you will give favorable consideration to this revised version and find the manuscript acceptable for publication in *Microbiology Spectrum.*

Reviewer #1 (Comments for the Author):

1. As per recommended nomenclature, please use CVA6 and not CV-A6 throughout the manuscript. Kindly read the article below for more specific guidance. "Simmonds et al., 2020. Recommendations for the nomenclature of enteroviruses and rhinoviruses. Arch Virol. 2020 Mar;165(3):793-797. doi: 10.1007/s00705-019-04520-6."

Response: we thank the reviewer for nomenclature correction. We have used CVA6 throughout the manuscript after checking. We appreciate the reference provided by the reviewer.

2. The authors still did not show irrefutable evidence that the samples did not contain other enteroviruses and especially members of the EV-A species.

Response: After assembly and re-analysis, we found that in some individual samples, the

sequencing data contained cases where CVA6 was inaccurately identified as EV-A. Additionally, other species of enterovirus A (such as EV-A71) were indeed detected, but these sequences did not cover the entire genome of EV-A71. Therefore, although other sequences of enterovirus A were identified, this does not mean that these viruses share a common, active infection with CVA6. Please refer to denovo_blast.xlsx and the response to your #7 comment below.

3. Figure 4: Please add schematic representation of genome architecture to panels A -I. This will help readers better understand what part of the genome the different contigs represent.

Response: Genome architecture was added to panels A-I of Figure 4.

4. Lines 77-80: Please cite the source of the enterovirus (EV) typing criteria described here.

Response: the reference (#10 in the manuscript) was cited for the EV typing criteria.

10. “Oberste MS, Maher K, Kilpatrick DR, Pallansch MA. Molecular evolution of the human enteroviruses: correlation of serotype with VP1 sequence and application to picornavirus classification. J Virol. 1999;73(3):1941-8.”

5. Lines 148-149: What was used for cDNA; Virus specific primers or oligo(dT) or both? Please be as clear as possible to ensure readers can independently reproduce the method.

Response: we appreciate the reviewer’s rigorous review on “Methods”. The previous version was not clear enough in specifying it. Accordingly, we have revised this part in to “1 µL of 50 µM oligo(dT)” to indicate only oligo(dT) was used for cDNA synthesis (line 147-148).

6. Lines 175-185: Two different ONT library prep kits and workflows were mentioned. Which was used and for what. Please be as clear as possible to ensure readers can independently reproduce the method.

Response: we appreciate the reviewer’s concern on the workflows. Accordingly, we have made explanation for it to make it as clear as possible. Please refer to line 185-189 for the updated version.

7. Lines 205-226: The are quite a few red flags in this region

a. Demultiplexing was first done using Guppy. Why do it again with Porechop?

Response: The main purpose of using Porechop is to remove the cross-linked sequences. This method has the parameter for removing the original cross-linked sequences - "discard_middle", and it has achieved certain results. This part has been carefully modified in line 209-211.

b. What does this mean: "The software for enterovirus reference genomes was obtained from the NCBI website (https://www.ncbi.nlm.nih.gov/) for analysis"? What software are you referring to?

Response: As suggested, the detail was provided as follows:

“The clean data were aligned against enterovirus reference genomes obtained from the NCBI website (https://www.ncbi.nlm.nih.gov/) using Minimap2 (v2.30) [45] for the genome coverage and sequencing depth comparison.” in line 219-222

c. You have already assembled the genome using Flye. How was the genome polished? What do you need Freebayes and Bcftools for after you already have your assembled genome from Flye and have contigs? Please be very clear about your methods section.

Response: we thank the reviewer for pointing out this issue. Accordingly, the description of the Flye software has been removed, as the previous assembly was intended to determine the presence

of other Enterovirus A. The description was modified as follows:
"The reference genome with highest genome coverage and sequencing depth of the aligned results was considered as optimal reference genome, and the complete genome sequence of CVA6 was subsequently assembled using Bcftools (V1.12) based on the optimal reference genome [47]" in line 223-225.

d. Lines 78-79 already mentioned the typing criteria [>75% similarity in the VP1 genomic region]. What do you mean by "optimal genome selection in lines 218-219. Shouldn't the contigs assembled using Flye for each sample be your reference for each sample should you want to estimate target to total reads ratio?

Response: this part was modified as follows: "The reference genome with highest genome coverage and sequencing depth of the aligned results was considered as optimal reference genome, and the complete genome sequence of CVA6 was subsequently assembled using Bcftools (V1.12) based on the optimal reference genome [46]. The sequence alignments were performed by using MUSCLE (v35.1.8) [48]" in line 223-226.

e. Mapping to a reference you see fit (or consider optimal) based on BLASTn hits can force the similarity found in figures 6 and 7.

Response: Blastn was only used to identify whether the assembled contigs belonged to other Enterovirus A, whereas the genomes in Figures 6 and 7 were obtained through reference-based assembly using MinImap2 and Bcftools, and thus show such high similarity.

f. No recombination analysis was carried out to rule out potential recombination events (Chimeras). Furthermore, no nucleotide substitution model testing was done and phylogenetic trees were not constructed to test P1 vs P2 or P1 vs P2-P3 to rule out recombination. The observation with AS289G2 vs AS289G3 in Figure 6C strongly suggest the need for recombination analysis.

Response: we thank the reviewer for the valuable comments regarding to AS289 genome. Recombination analysis using Simplot (v3.5.1) was conducted and no recombination signal detected. As suggested, phylogenetic trees were constructed using P1, P2, P3 and genome, respectively (supplementary figure S3). The sequence discrepancies between AS289 and the other 7 samples were predominantly concentrated in P3 regions as the phylogenetic trees indicated, highlighting the necessity of constant genomic surveillance of CVA6. The above content was added in the text (line 345-351)

g. Note that the supplementary data [see image below] shows evidence that suggest other EV-As like EV-A71 was present in some samples. Please carefully reexplore the data.

Response: We sincerely thank the reviewer for this careful observation and constructive suggestion, which has helped us improve the rigor of our manuscript. We have comprehensively re-explored the raw sequencing data, de novo assembled contigs, and BLAST annotation results as requested (***Supplementary Data 3 for reviewer #1***). We re-blast the contig568 of barcode35 (AS217_2021M), and the results are shown in the following table:

Select for downloading or viewing reports	Description	Scientific Name	Max Score	Total Score	Query Cover	E value	Per. Ident	Acc. Len	Accession
Select seq PP627061.1	Coxsackievirus A6 isolate AS009/NC/CHN/2021 polyprotein gene, complete cds	Coxsackievirus A6	1609	2729	48%	0	99.11%	7425	PP627061.1
Select seq PP627064.1	Coxsackievirus A6 isolate AS270/NC/CHN/2020 polyprotein gene, complete cds	Coxsackievirus A6	1604	2718	48%	0	99.00%	7425	PP627064.1
Select seq OR340524.1	Enterovirus A isolate HA/2020-9/CHN/CVA6/2020 capsid protein VP1 gene, partial cds	Enterovirus A	1592	2718	48%	0	99.21%	915	OR340524.1

Select seq PP627067.1	Coxsackievirus A6 isolate AS597/NC/CHN/2021 polyprotein gene, complete cds	Coxsackievirus A6	1592	2707	48%	0	98.78%	7430	PP627067.1
Select seq PP627066.1	Coxsackievirus A6 isolate AS497/NC/CHN/2020 polyprotein gene, complete cds	Coxsackievirus A6	1592	2707	48%	0	98.78%	7426	PP627066.1
Select seq LC813389.1	Coxsackievirus A6 Dy2020-105 VP1 gene for polyprotein, partial cds	Coxsackievirus A6	1587	2707	48%	0	99.10%	915	LC813389.1
Select seq OR507475.1	Coxsackievirus A6 isolate 2020HB31CA6 VP1 gene, partial cds	Coxsackievirus A6	1587	2713	48%	0	99.10%	915	OR507475.1
Select seq OL470912.1	Coxsackievirus A6 isolate CV-A6/HA20057/2020/Beijing/Tongzhou VP1 gene, partial cds	Coxsackievirus A6	1587	2707	48%	0	99.10%	915	OL470912.1
Select seq LC813392.1	Coxsackievirus A6 DY2020-113 VP1 gene for polyprotein, partial cds	Coxsackievirus A6	1581	2696	48%	0	98.98%	915	LC813392.1
Select seq LC813391.1	Coxsackievirus A6 DY2020-107 VP1 gene for polyprotein, partial cds	Coxsackievirus A6	1581	2702	48%	0	98.98%	915	LC813391.1
Select seq OR394970.1	Coxsackievirus A6 strain CVA6/19275/CC/CHN/2019, complete genome	Coxsackievirus A6	1581	2679	48%	0	98.55%	7425	OR394970.1
Select seq OR507510.1	Coxsackievirus A6 isolate 2021HB3CA6 VP1 gene, partial cds	Coxsackievirus A6	1581	2696	48%	0	98.98%	915	OR507510.1

From the results, contig568 of barcode35 is presumably the CVA6 sequence. Previously, this sequence was annotated as EV-A, which might have been mistakenly assigned to a sequence name with ambiguous classification. The results in the table have been corrected.

Some other samples, such as barcode38 (AS297_2022M), do have the contig9 sequence that belongs to EV-A71. However, no complete or near-complete genome of any enterovirus (including EV-A71) other than CVA6 was assembled in this study, and CVA6 was the only viral strain with high sequencing depth and full genome coverage across all samples. In AS297_2022M, the 1X genomic coverage of the EV-A71 (ON502191.1) strain was less than 70% (as shown in the table below), and a complete genome could not be obtained. This indicates that although the sample contains the sequence of EV-A71, it does not mean that the sample contains an active EV-A71. It cannot prove the existence of CVA6 co-infection with other active viruses. The same situation applies to contig 76 in sample barcode 41 (AS153_2020H).

Coverage depth	Sequence length	Ratio
>= 1X	4.5 K	68.06%
>= 10X	4.5 K	68.06%
>= 50X	4.2 K	63.08%
>= 100X	4.0 K	60.29%
>= 200X	2.5 K	38.42%

8. Table 1: For easier reading, if possible, kindly express the values in the columns CVA6/Yield and Genome coverage as percentages. This will make it easier for readers to connect the text and the table. For example, in lines 298-300, you mention 99.1% but in Table 1, the value is 0.991. Having both as percentages makes the connection easier for the reader. Also please site Table 1 on line 300.

Response: we thank the review for this suggestion. The values of CVA6/Yield and genome coverage were expressed as percentage. And the Table 1 was cited on line 309.

9. Figure 6A: There are 18 sequences instead of 16 as mentioned in the text. There are two sequences in the tree that stand out because they are represented with accession numbers. The figure details mention they are the top 2 BLASTn hits. Why are they present in 6A and 6B but not in 6C. Do you mean all 16 sequences [with divergence approaching 6% according to figure 6B] have these same 2 sequences as their top 2 hits in GenBank?

Response: we appreciate the reviewer's concern on this issue. In the first-round peer review, the reviewer asked us to kindly add a table that shows BLASTn results of all the contigs alongside their pairwise identity with the top 2 hits in GenBank. We might have misunderstood the meaning of the reviewer's suggestion, then only 2 CVA6 reference strains (top 2 hits for AS377) were selected. In the revised figure 6, no references were included for pairwise distance analysis, as representative CVA6 strains were included to build the phylogenetic tree in figure 7.

10. Please mention the tool used for Figure 6B in the bioinformatics subsection of the methods section.

Response: The method was added at the end of section "Bioinformatic analysis of Illumina sequencing data" (line 240-241).

11. There is no table similar to Table 1 describing the statistics for the data generated for Illumina sequencing. Please add the data. The Illumina data also needs to be de-novo assembled and subjected to recombination analysis. Furthermore, if possible, kindly mention if the varying region of AS289G2 vs AS289G3 highlighted in Figure 6C was re-sequenced using the sanger technology. In addition, is that variation still present if both sequences [AS289G2 and AS289G3] were de-novo assembled?

Response: according to the reviewer's suggestion, we added the statistics of Illumina sequencing in Supplementary Table S5. In this study, we used reference-based assembly as described in Methods (line 231-234), references were provided in Supplementary Table S5. In order to confirm the genome sequence of AS289, sanger sequencing was done for sample AS289. Using the assembled genome of AS289 as reference, Illumina sequencing raw data of AS289G2 was re-assembled. We found there is only two sites variation between G2 and G3 (Figure 6C). This result leads to a necessary modification of Figure 6B and 6C. We have provided all necessary raw data (***Supplementary Data 1 for reviewer #1***) and agarose gel electrophoresis of PCR products (primers refer to https://doi.org/10.1038/srep11700) of the AS289 sample for your review.

As suggested, de-novo assembly was conducted for both AS289G2 and AS289G3 data. De novo assembly of AS289G2 suggested that all contigs of enterovirus belong to CVA6 with supportive blastn results (Response Fig. 1, ***supplementary data 2 for reviewer #1***). As for AS289G3 de novo assembly, response has provided evidence in the reviewer's "**#7-g comment**" above. Based on de novo assembly of AS289G2 and AS289G3, that variation is not present.

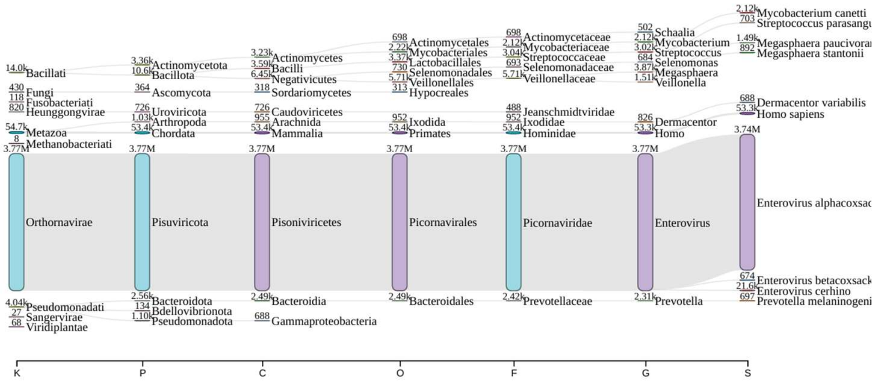

Response Fig. 1 Sankey diagram of species by de novo assembly of AS289G2

12. Line 350: "2A-3C genomic region (approximately 3500-6000 nucleotides)"

a. Do you really mean (approximately 3500-6000 nucleotides) or (approximately nucleotides 3500-6000)?

Response: Thanks, we have revised this part in line 361-366. Here, we meant approximately

nucleotides 3500-6000. After revision, we have carefully modified this part based on the corrected analysis on AS289 consensus sequences associated with Figure 6.

b. The region with lower sequencing depth in Figure 4B-H is generally between nucleotides 5000 and 7500 not 3500-6000. Please re-examine the data.

Response: this part was rewritten due to re-analysis of the updated sequences of AS289G2 in line 361-366.

c. As opposed to line 353, nucleotides 5000 to 7500 encode nonstructural proteins and these usually do not have higher sequence diversity. Usually it is the P1 region (which encodes structural proteins) that has higher sequence diversity.

Response: we agree with the reviewer on the sequence diversity. This part was rewritten due to re-analysis of the updated sequences of AS289G2 in line 361-366. And adjustments were made throughout the discussion in line 431-434.

d. Please add a schematic representation of genome architecture to panels A -I. This will help readers better understand what part of the genome the different contigs represent.

Response: Schematics of genome architecture has been added for panels A-I.

13. Please make adjustments throughout the discussion section based on the comments above.

Response: adjustments were made in the discussion in line 431-434 as follows:

"In this study, we observed relatively low sequencing depth between nucleotides 5000 and 7500 (the 3A-3D region) (Figure 4), a genomic region where recombination events occur frequently [16]. This finding highlights the need for primer optimization for this specific region in future studies."

14. Lines 436-439: PCR amplification should make it easier to detect minority variants. Please check literature on this and be more precise on what you mean.

Response: As suggested, we modified this part as follows: "Second, although PCR amplification is an indispensable step to achieve adequate analytical sensitivity for viral target detection in clinical specimens (such as anal swabs with high background nucleic acid and low viral load), it introduces inherent technical limitations that fundamentally compromise the reliable detection and quantitative characterization of low-frequency minority variants in viral quasispecies [57, 58]." In line 431-443

Reviewer #2 (Comments for the Author):

The authors performed a significant rewrite of the manuscript, and I now find it to be much improved. It is well-structured and easy to read and follow. Figures are helpful, and conclusions are now mostly supported by the data. A few issues remain to be addressed.

Response: we thank the reviewer for the valuable comments and constructive suggestion. In the revised manuscript, we carefully take all the comments into consideration and revised them point by point as follows.

I cannot arrive at the same conclusion as the authors as it relates to the data volume recommendations. Despite stating that it was removed as unsupported in the responses to reviewer comments, the authors retain the 50 Mb cutoff on line 297 and 303. In fact, the authors state that a CVA6 data volume of >50 Mb reliably resulted in >95% genome coverage. In fact, every sample

tested resulted in >95% genome coverage. For all the data supports, 13.5 Mb CVA6 reliably resulted in 95% genome coverage, and for all we know, the number may be lower than that. The yield recommendations are based on this arbitrary 50 Mb cut-off and therefore equally unsupported. The chosen brackets are also arbitrary as they could be set anywhere along the Ct vs reads ratio regression line (say for example Ct 22 - TRR ~60% - min yield 85 Mb) and having a lower recommendation for Ct >28 than the upper range for Ct 25-28 makes no sense. Further, the 'correlation' between CVA6 data volume and genomic coverage is minimal and unproven. No statistical tests were applied to the correlation, and visually, the slope of the regression line would be small with significant residuals on either side. Lastly, the discussion in 394-398 is based on this data, which in my view does not support the conclusion. The following changes should be made to eliminate these unsupported guidelines:

Response: we thank the reviewer for valuable suggestions, particularly on data volume recommendations. According to the reviewer's comments, we carefully made revision to avoid inaccurate data demonstration or confusing expression.

• Remove all cut-off (horizontal and vertical) lines in Fig. 5A and 5B

Response: the Fig 5 was carefully modified and cut-off removed as recommended by the reviewer.

• Remove all language suggesting that a specific data volume results in a specific amount of genomic coverage (including complete or 95%) unless appropriately supported by statistical tests

Response: according to the reviewer's comments, we have carefully modified the expression of sequencing data to avoid connection without statistical tests. We have reconstructed the section "Data volume and target reads ratio " to remove all unsupported expression as follows:

"The relationship between Ct value, CVA6 data volume, target reads ration, and genomic coverage was systematically analyzed. As shown in Figure 5A, A lower Ct value, suggesting higher viral load, results in a higher CVA6 data volume yield and a higher target reads ratio. Despite this variation in enrichment efficiency, achieving complete genomic coverage depended primarily on obtaining a sufficient absolute yield of CVA6-specific reads. As shown in Figure 5B, the genome coverage reached to 95.5%-100% with a wide range of CVA6 data volume (13.5-214.8 Mb), which indicated there may be a correlation between CVA6 data volume and genomic coverage. Together, these data suggest that a minimum amount of CVA6 data volume may be necessary to achieve complete genomic coverage and that higher Ct value samples may have to be sequenced to higher total sequencing yield to achieve complete (or near complete) coverage. Thus, it may be possible to establish minimum sequencing data volume guidance either independently or based on Ct, which may result in more efficient resource allocation." In line 303-316

• Remove all language based on unproven CVA6 data volume cut-offs. This includes recommendations of certain total sequencing yields based on Ct value in the abstract, results, and discussion sections

This ultimately results in the removal of the entirety of the data volume and read ratio section of the results (and affiliated discussion on line 394-398, as well as the abstract), yet the data itself is worth presenting. In my view, the following conclusions can be drawn from the data as presented:

• A lower Ct value results in a higher CVA6 data volume yield and a higher target reads ratio

• There may be (do NOT be definitive unless supported with statistical analysis) a correlation between CVA6 data volume and genomic coverage

• Together, these data suggest that a minimum amount of CVA6 data volume may be necessary to achieve complete coverage and that higher Ct value samples may have to be sequenced to higher total sequencing yield to achieve complete (or near complete) coverage

• It may be possible to establish minimum sequencing data volume guidance either independently or based on Ct, which may result in more efficient resource allocation

Response: we thank the reviewer for valuable and constructive suggestions on data volume section. We have carefully checked the CVA6 data volume cut-offs and recommendations, then removed all language based on unproven CVA6 data volume cut-offs. Based on the reviewer's suggestion, we have drawn several conclusions and recomposed this section in line 303-316.

Finally, the data in Fig 5B appears to be incorrect. As stated in my previous comments, all genome coverage data is shown in complete percentage points, whereas actual data is resolved to 0.1%. This leads to an entirely inaccurate plot. As stated previously, there is one data point at 95% - this must be the lowest data point at 95.5%. However, there is also only 1 data point at 96%, but the 'next highest' data point in the table is 96.5%, of which there are 4 in the table. The lack of attention to detail in this figure, and the lack of correction based on prior comments, is concerning.

Response: we thank the reviewer for data scrutiny, data in Fig 5B were carefully checked and corrected in accordance with data in table 1.

The continued use of the term 'viral load' when referring to Ct values remains inaccurate. A Ct value is not a viral load, not even a semi-quantitative one. A Ct value is a semi-quantitative value correlating with a viral load. A viral load is a specific term and while we can measure viral loads by calculating backwards from the Ct value, that does not make a Ct value a viral load. A bank account's interest yield is directly proportional to the account balance, yet it is not in fact the account balance, though it may be calculated from it. This may seem like a petty point, but the conflation of Ct values with viral loads continues to perpetuate false equivalency and misuse of Ct values in the infectious disease diagnostic field. Please remove all instances of calling a Ct value a viral load. Where needed for clarity, it would be appropriate to use phrases such as "lower Ct values, suggesting higher viral load" or similar.

Response: we really thank the reviewer for pointing out the incorrect use of term "viral load" as in the manuscript. As suggested by the reviewer, we have changed the expression to avoid the misuse. By careful checking each section, we have used phrase "lower or higher Ct values" highlighted in the revised text.

Minor points:

• Line 126 still refers to anal swabs - this does not appear to be a sample type that was used

Response: anal was deleted in line 125 as the sample selected in this study didn't contain anal swabs, even though anal swabs were one of the sample types in surveillance system.

• Please remove the connecting line from the scatter plot in Fig. 5B.

Response: we thank the reviewer for data scrutiny, data in Fig 5B were carefully checked and corrected in accordance with data in table 1, connecting line was removed.

• In the text describing Fig. 6A/B, please describe the purpose of the additional 2 isolates from GenBank - are those there as an outgroup? What is the reader supposed to learn from their

inclusion?

Response: we appreciate the reviewer's concern on the reference strains from GenBank. As a number of CVA6 reference strains were used for phylogenetic analysis in Figure 7. In Figure 6 (R1), only sequences obtained in this study were used to build the phylogenetic tree and for subsequent similarity analysis.

Reviewer #3 (Comments for the Author):

The manuscript has improved a lot since first submission, I do have a few minor comments left:

Response: we appreciate that the reviewer recognized most revisions we have made in the last round response and thank for the reviewer's rigor scrutiny. According to the comments we have addressed the concern point by point.

L205: What is the name of the software for 'enterovirus reference genomes obtained from NCBI)'?

Response: The original sentence was obscured. The section including this sentence has been rewritten in line 222-228.

L220: add reference for iVar (https://link.springer.com/article/10.1186/s13059-018-1618-7)

Response: this is a useful paper for this study to cite. The reference the reviewer recommended was cited to iVar in methods (#52 reference) in line 235.

L285-289: From Table 1 and Figure 5B it seems that all samples got a high coverage regardless of the CVA6 data volume, why the recommendation for >50Mb and not >10Mb as this is the lowest data volume that gave a high coverage?

Response: according to the reviewer's comments, we have carefully modified the expression of sequencing data to avoid connection without statistical tests. We have reconstructed the section "Data volume and target reads ratio " to remove all unsupported expression as follows:

"The relationship between Ct value, CVA6 data volume, target reads ration, and genomic coverage was systematically analyzed. As shown in Figure 5A, A lower Ct value, suggesting higher viral load, results in a higher CVA6 data volume yield and a higher target reads ratio. Despite this variation in enrichment efficiency, achieving complete genomic coverage depended primarily on obtaining a sufficient absolute yield of CVA6-specific reads. As shown in Figure 5B, the genome coverage reached to 95.5%-100% with a wide range of CVA6 data volume (13.5-214.8 Mb), which indicated there may be a correlation between CVA6 data volume and genomic coverage. Together, these data suggest that a minimum amount of CVA6 data volume may be necessary to achieve complete genomic coverage and that higher Ct value samples may have to be sequenced to higher total sequencing yield to achieve complete (or near complete) coverage. Thus, it may be possible to establish minimum sequencing data volume guidance either independently or based on Ct, which may result in more efficient resource allocation." In line 303-316

L384-388: What would be the recommendations to get to these data volumes when you have a set of samples that you want to multiplex but they have different Ct values (some >28 and some <25)? Pool them into non-equimolar amounts?

Response: For samples with a Ct value difference of ≥3 cycles (corresponding to an at least 8-fold

difference in target nucleic acid concentration), we strongly recommend a non-equimolar differential pooling strategy as the core approach, supplemented by optimized library preparation and target enrichment workflows, rather than standard equimolar pooling. This is the only reliable method to avoid excessive data from high-titer (low Ct) samples and insufficient valid data from low-titer (high Ct, >28) samples.

Figure 5B: It is still not entirely clear to me why there is a blue dotted line on y=0.97, is this the minimum coverage chosen to represent high coverage and therefore you chose to visualize this? Also in the figure description for Figure 5A and B, "The intersection points of the red dotted line and the blue dotted line lie on the fitted straight line." and "The intersection points of the red dotted line and the blue dotted line lie on the lowest genomic coverage point." do not actually describe what these lines represent.

For figure 5A, the red dotted lines indicate the Ct value cut offs and the blue dotted lines the chosen target reads ratio for each Ct. But this is not clear from the description above, so please edit the description.

Response: We appreciate the reviewer’s rigor scrutiny. Indeed, the figure and legend were indeed obscured. Therefore, we modified the figures and description in line 319-322. Accordingly, the section “Data volume and target reads ratio” (line 303-316) was rewritten.

Figure 6 and 7: change "phylogenetic distance" to "nucleotide substitutions/site" as this is what the scale bar is actually representing

Response: the description was modified accordingly in figure legends.

Re: Spectrum03246-25R2 (Targeted sequence capture of coxsackievirus A6 using Nanopore sequencing directly from clinical specimens)

Dear Dr. Hui Li:

Thank you for submitting the revised manuscript and responses to the reviewers' comments. As noted in the reviewer comments, please make sure that you describe the detection of other EV-A members in addition to CVA6 and modify the statements on lines 397-398 to reflect the observation accordingly and correctly.

Below you will find the reviewer comments and instructions from the Spectrum editorial office.

Please return the manuscript within 60 days; if you cannot complete the modification within this time period, please contact me. If you do not wish to modify the manuscript and prefer to submit it to another journal, notify me immediately so that the manuscript may be formally withdrawn from consideration by Spectrum.

Revision Guidelines
To submit your modified manuscript, log into the submission site at https://spectrum.msubmit.net/cgi-bin/main.plex. Go to Author Tasks and click the appropriate manuscript title to begin. The information you entered when you first submitted the paper will be displayed; update this as necessary. Note the following requirements:

• Upload point-by-point responses to the issues raised by the reviewers in a file named "Response to Reviewers," NOT in your cover letter.
• Upload a compare copy of the manuscript (without figures) as a "Marked-Up Manuscript" file.
• Upload a clean .DOC/.DOCX version of the revised manuscript and remove the previous version.
• Each figure must be uploaded as a separate, editable, high-resolution file (TIFF or EPS preferred), and any multipanel figures must be assembled into one file.
• Any supplemental material intended for posting by ASM should be uploaded with their legends separate from the main manuscript. You can combine all supplemental material into one file (preferred) or split it into a maximum of 10 files with all associated legends included.

For complete guidelines on revision requirements, see our Submission and Review Process webpage. Submission of a paper that does not conform to guidelines may delay acceptance of your manuscript.

Data availability: ASM policy requires that data be available to the public upon online posting of the article, so please verify all links to sequence records, if present, and make sure that each number retrieves the full record of the data. If a new accession number is not linked or a link is broken, provide Spectrum production staff with the correct URL for the record. If the accession numbers for new data are not publicly accessible before the expected online posting of the article, publication may be delayed; please contact production staff (Spectrum@asmusa.org) immediately with the expected release date.

Publication Fees: For information on publication fees and which article types are subject to charges, visit our website. If your manuscript is accepted for publication and any fees apply, you will be contacted separately about payment during the production process; please follow the instructions in that e-mail. Arrangements for payment must be made before your article is published.

ASM Membership: Corresponding authors may join or renew ASM membership to obtain discounts on publication fees. Need to upgrade your membership level? Please contact Customer Service at Service@asmusa.org.

The ASM Journals program strives for constant improvement in our submission and publication process. Please tell us how we can improve your experience by taking this quick Author Survey.

Thank you for submitting your paper to Spectrum.

Sincerely,
My Phan
Editor
Microbiology Spectrum

Reviewer #1 (Comments for the Author):

1] In your response to reviewers, you indicated that some samples may have contained other EV-A members alongside CVA6. It would be advisable to acknowledge this in the manuscript and inform readers that the assay may be somewhat promiscuous, with strong potential to be expanded to amplify other EV-A members.

2] Please read through the manuscript and make minor grammatical corrections
3] Ensure all sequences (ONT and Illumina) have been submitted to GenBank and SRA.

Reviewer #2 (Comments for the Author):

My comments have been addressed, and the conclusions are now generally supported by the data. A couple of final comments:
- Lines 308-310, the fact that genome coverage reached 95.5%-100% with a wide range of CVA6 data volumes does not in fact indicate there may be correlation - quite the opposite. Instead consider: Although genome coverage reached 95.5%-100% with a wide range of CVA6 data volumes, most of the highest coverage levels were observed at higher CVA6 data volumes, suggesting that there may be a correlation between CVA6 data volumes and genomic coverage.
- Fig 5 legend: "The intersection points of the data point and the blue dotted line represented the lowest genomic coverage in this study" this is awkward. I don't think the blue line adds anything, but if retained, "The blue dotted line represented the lowest genomic coverage in this study" would be sufficient
- Line 397-398, the authors still describe a correlation between Ct values and genomic coverage. This is not supported by the data, as stated in my previous comments. What is observed is a correlation between Ct values and CVA6 data volume, and a potential trend toward higher genome coverage with higher CVA6 data volumes.

Reviewer #3 (Comments for the Author):

In rewriting the section "Data volume and target reads ratio" (line 303-316), it appears the authors have made their descriptions/conclusions too vague and applicable to almost any sequencing experiment:
- " which indicated there may be a correlation between CVA6 data volume and genomic coverage": increasing sequencing depth inherently improves the likelihood of capturing low-abundance targets and resolving difficult-to-sequence regions, rather than representing a finding specific to this dataset.
- "these data suggest that a minimum amount of CVA6 data volume may be necessary to achieve complete genomic coverage": this is somewhat self-evident, as sufficient sequencing depth is inherently required to recover a complete genome.
- "it may be possible to establish minimum sequencing data volume guidance either independently or based on Ct": this lacks any actionable guidance, I would rather say that in your experiments a CVA6 data volume of 10Mb was already enough to get to a high genome coverage.
In addition, I would remove the first two generic statements.

Spectrum03246-25 R2

My comments have been addressed, and the conclusions are now generally supported by the data. A couple of final comments:

- Lines 308-310, the fact that genome coverage reached 95.5%-100% with a wide range of CVA6 data volumes does not in fact indicate there may be correlation – quite the opposite. Instead consider: Although genome coverage reached 95.5%-100% with a wide range of CVA6 data volumes, most of the highest coverage levels were observed at higher CVA6 data volumes, suggesting that there may be a correlation between CVA6 data volumes and genomic coverage.
- Fig 5 legend: “The intersection points of the data point and the blue dotted line represented the lowest genomic coverage in this study” this is awkward. I don’t think the blue line adds anything, but if retained, “The blue dotted line represented the lowest genomic coverage in this study” would be sufficient
- Line 397-398, the authors still describe a correlation between Ct values and genomic coverage. This is not supported by the data, as stated in my previous comments. What is observed is a correlation between Ct values and CVA6 data volume, and a potential trend toward higher genome coverage with higher CVA6 data volumes.

Point-by-point response

Subject: Spectrum03246-25R2 Decision Letter

Re: Spectrum03246-25R2 (Targeted sequence capture of coxsackievirus A6 using Nanopore sequencing directly from clinical specimens)

Dear Dr. Hui Li:

Thank you for submitting the revised manuscript and responses to the reviewers' comments. As noted in the reviewer comments, please make sure that you describe the detection of other EV-A members in addition to CVA6 and modify the statements on lines 397-398 to reflect the observation accordingly and correctly.

Below you will find the reviewer comments and instructions from the Spectrum editorial office.

Please return the manuscript within 60 days; if you cannot complete the modification within this time period, please contact me. If you do not wish to modify the manuscript and prefer to submit it to another journal, notify me immediately so that the manuscript may be formally withdrawn from consideration by Spectrum.

Response: Dear Dr. Phan, thank you very much for your letter and for giving us the privilege of revising our manuscript. We greatly appreciate the time and effort you and the reviewers have dedicated to our work through two rigorous rounds of peer review.

We have carefully read all the instructions and the reviewers' comments, and followed the revision guidelines provided by the editorial office, including preparing a point-by-point response, a marked-up manuscript, and a clean revised version.

Thank you again for your continued support and for the opportunity to publish in Microbiology Spectrum.

Reviewer #1 (Comments for the Author):

1] In your response to reviewers, you indicated that some samples may have contained other EV-A members alongside CVA6. It would be advisable to acknowledge this in the manuscript and inform readers that the assay may be somewhat promiscuous, with strong potential to be expanded to amplify other EV-A members.

Response: Thank you for this valuable suggestion. We agree that acknowledging the potential presence of other EV-A members in some clinical samples is important, and we also recognize that the primer design may exhibit some degree of cross-reactivity within the EV-A species. This observation points to a promising opportunity to adapt the current assay for broader enterovirus detection and genotyping. To address this comment, we have revised the Discussion section to include the following paragraph:

"In this study, we focused exclusively on CVA6-positive clinical samples. However, our primer design was based on conserved regions among multiple CVA6 strains, and sequence alignment suggests that several primer binding sites may also be conserved across other members of the Enterovirus A species. During library preparation, we occasionally detected low-abundance reads mapping to other EV-A serotypes such as EV-A71 (data not shown), indicating co-infection or suboptimal amplification of related viruses. While the current protocol was not formally validated for these targets, this incidental cross-reactivity suggests that the assay may be moderately promiscuous within EV-A. With minor primer adjustments or dedicated validation, the same tiling amplicon approach could be expanded to enable simultaneous or broad-spectrum genomic surveillance of multiple EV-A serotypes directly from clinical specimens." in line 449-459.

We believe this addition acknowledges the reviewer's point and appropriately highlights the potential for future expansion of the method.

2] Please read through the manuscript and make minor grammatical corrections
Response: we thank the reviewer's suggestion. Grammar was thoroughly checked and corrections were made accordingly.

3] Ensure all sequences (ONT and Illumina) have been submitted to GenBank and SRA.
Response: Sequences in this study including raw data were submitted to GenBank and SRA as stated in Data Availability.

Reviewer #2 (Comments for the Author):

My comments have been addressed, and the conclusions are now generally supported by the data. A couple of final comments:
- Lines 308-310, the fact that genome coverage reached 95.5%-100% with a wide range of CVA6 data volumes does not in fact indicate there may be correlation - quite the opposite. Instead consider: Although genome coverage reached 95.5%-100% with a wide range of CVA6 data volumes, most of the highest coverage levels were observed at higher CVA6 data volumes, suggesting that there may be a correlation between CVA6 data volumes and genomic coverage.
Response: we appreciate the reviewer's constructive suggestions, and consider the revision accordingly as follows: "Although genome coverage reached 95.5%-100% with a wide range of CVA6 data volumes (Figure 5B), most of the highest coverage levels were observed at higher CVA6 data volumes, suggesting a trend between CVA6 data volumes and genomic coverage." in line 310-312.

- Fig 5 legend: "The intersection points of the data point and the blue dotted line represented the lowest genomic coverage in this study" this is awkward. I don't think the blue line adds anything, but if retained, "The blue dotted line represented the lowest genomic coverage in this study" would be sufficient
Response: we thank the reviewer for pointing out the confusing information of the legend. We have corrected it as suggested by the reviewer as follows: "The blue dotted line represented the lowest genomic coverage in this study" in line 322.

- Line 397-398, the authors still describe a correlation between Ct values and genomic coverage. This is not supported by the data, as stated in my previous comments. What is observed is a correlation between Ct values and CVA6 data volume, and a potential trend toward higher genome coverage with higher CVA6 data volumes.

Response: the description of the correlation between Ct values and genomic coverage is inappropriate. Then this part was revised as follows:

"A correlation was observed between Ct values and the absolute yield of CVA6-specific data (Figure 5). Furthermore, a trend toward higher genome coverage was noted with increasing CVA6 data volumes. It may be possible to determine minimum sequencing data volume requirements by using Ct values alone or in combination with high-throughput sequencing metrics." in line 398-402.

Reviewer #3 (Comments for the Author):

In rewriting the section "Data volume and target reads ratio" (line 303-316), it appears the authors have made their descriptions/conclusions too vague and applicable to almost any sequencing experiment:

- " which indicated there may be a correlation between CVA6 data volume and genomic coverage": increasing sequencing depth inherently improves the likelihood of capturing low-abundance targets and resolving difficult-to-sequence regions, rather than representing a finding specific to this dataset.
- "these data suggest that a minimum amount of CVA6 data volume may be necessary to achieve complete genomic coverage": this is somewhat self-evident, as sufficient sequencing depth is inherently required to recover a complete genome.
- "it may be possible to establish minimum sequencing data volume guidance either independently or based on Ct": this lacks any actionable guidance, I would rather say that in your experiments a CVA6 data volume of 10Mb was already enough to get to a high genome coverage.

In addition, I would remove the first two generic statements.

Response: we sincerely appreciate the reviewer's constructive comments and suggestions. We rewrote this section to avoid generic statements and inaccurate descriptions/conclusions as follows: "The relationship between Ct value, CVA6 data volume, target reads ratio, and genomic coverage was analyzed. As shown in Figure 5A, a lower Ct value, suggesting higher viral load, was associated with a trend of higher CVA6 data volume yield and a higher target reads ratio. Despite this variation in enrichment efficiency, achieving complete genomic coverage depended primarily on obtaining a sufficient absolute yield of CVA6-specific reads. Although genome coverage reached 95.5%-100% with a wide range of CVA6 data volumes (Figure 5B), most of the highest coverage levels were observed at higher CVA6 data volumes, suggesting a trend between CVA6 data volumes and genomic coverage. In this dataset, a CVA6-specific data volume of approximately 10 Mb was already sufficient to achieve high genome coverage (>95%) for most samples (Figure 5B). These empirical observations from our experiments indicate that the capture protocol is highly efficient, and that even relatively modest CVA6 yields can produce near-complete genomes." in line 305-317.

Re: Spectrum03246-25R3 (Targeted sequence capture of coxsackievirus A6 using Nanopore sequencing directly from clinical specimens)

Dear Dr. Hui Li:

Your manuscript has been accepted for publication at Microbiology Spectrum. I am forwarding it to the ASM production staff for publication. Your paper will first be checked to make sure all elements meet the technical requirements. ASM staff will contact you if anything needs to be revised before copyediting and production can begin. Otherwise, you will be notified when your proofs are ready to be viewed.

Data Availability: ASM policy requires that data be available to the public upon online posting of the article, so please verify all links to sequence records, if present, and make sure that each number retrieves the full record of the data. If a new accession number is not linked or a link is broken, provide production staff with the correct URL for the record. If the accession numbers for new data are not publicly accessible before the expected online posting of the article, publication may be delayed; please contact ASM production staff immediately with the expected release date.

Publication Fees: For information on publication fees and which article types have charges, please visit our website. We have partnered with Copyright Clearance Center (CCC) to collect author charges. If fees apply to your paper, you will receive a message from no-reply@copyright.com with further instructions. For questions related to paying charges through RightsLink, please contact CCC at ASM_Support@copyright.com or toll free at +1-877-622-5543. CCC makes every attempt to respond to all emails within 24 hours.

ASM Membership: Corresponding authors may join or renew ASM membership to obtain discounts on publication fees. Need to upgrade your membership level? Please contact Customer Service at Service@asmusa.org.

Embargo Policy: A press release may be issued as soon as the manuscript is posted on the Spectrum Latest Articles webpage. The corresponding author will receive an email with the subject line "ASM Journals Author Services Notification" when the article is available online.

The ASM Journals program strives for constant improvement in our submission and publication process. Please tell us how we can improve your experience by taking this quick Author Survey.

Thank you for submitting your paper to Spectrum.

Sincerely,
My Phan
Editor
Microbiology Spectrum
